# Three-state molecular switch based on terarylene photo- or redox-induced reversible isomerisation†

**DOI:** 10.1039/d5sc02845k

**Published:** 2025-06-10

**Authors:** Nicolò Baggi, Anne Léaustic, Jean-Noël Rebilly, François Mavré, Eric Rivière, Christian Herrero, François Maurel, Pei Yu

**Affiliations:** a Université Paris-Saclay, CNRS, Institut de Chimie Moléculaire et des Matériaux d’Orsay 19 Avenue des Sciences Orsay 91400 France nicolo.baggi@upc.edu pei.yu@universite-paris-saclay.fr; b Université de Paris Cité, ITODYS, CNRS Paris F-75013 France

## Abstract

Multi-addressable molecular photoswitches whose isomerisation can be triggered not only by light, but also by other stimuli are appealing for the development of novel smart materials as well as for broadening the areas for their potential application. Diarylethenes (DAEs) are among the most studied switches for this purpose, since tailored functionalisation can make them responsive not only to UV or visible light, but also to other inputs, such as an electrochemical one. In this work, we synthesised five terarylene-based switches and investigated their photochemical and redox properties. Unlike their DAEs analogues, whose cyclisation upon an oxidation-reduction sequence is well-established, our systems undergo a similar oxidative ring-closing of the neutral open form to a doubly charged closed form while the subsequent reduction leads to ring-opening to the neutral open form. Moreover, the neutral closed form can also be re-opened by a catalytic amount of oxidant. With the support of theoretical modelling and cyclic voltammetry simulations, a general mechanism is proposed to rationalise this original bidirectional dual-responsive behaviour.

## Abstract 2

Terarylene-based photoswitches designed for photo- and redox-responsive behaviour undergo a three-state bidirectional isomerisation between their neutral open form and two different closed form isomers depending on the chosen stimulus.

## Graphical abstract

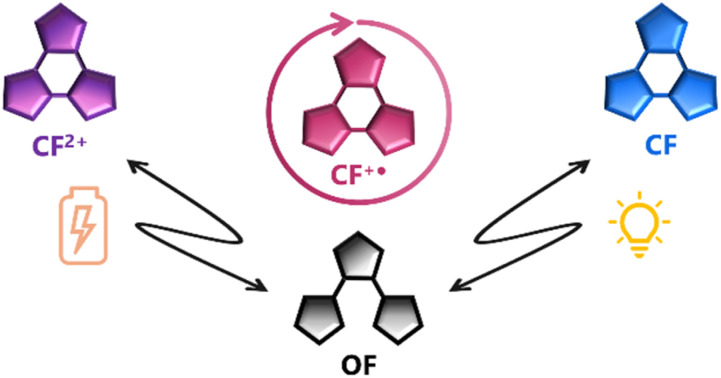

## Introduction

**Fig. 1:**
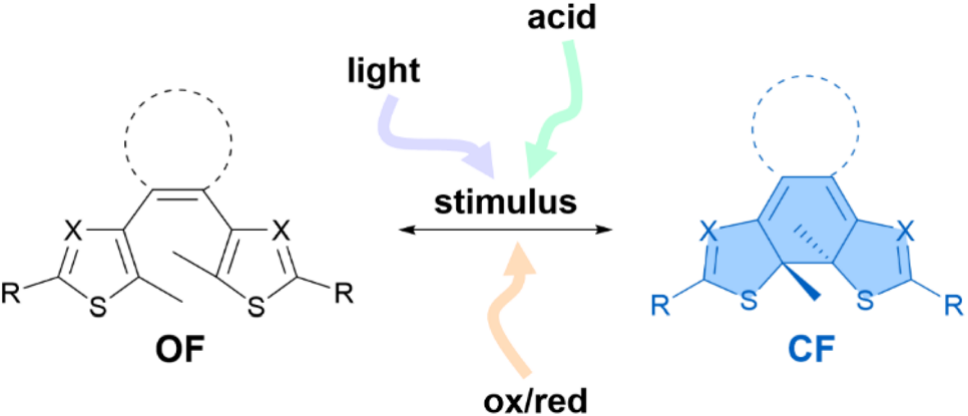
Schematic representation of the multi-responsive isomerisation of diarylethenes between the open form (OF, black) and the closed form (CF, blue) showing the possible stimuli inducing the isomerisation described in the literature. X = CH, N.

Molecular photochromic systems capable of responding to multiple stimuli to interconvert between at least two states are appealing for the development of smart materials.^1–9^ Among different photoswitches investigated for this purpose, diarylethenes (DAEs) are popular thanks to their excellent modulable properties in both isomeric states: the generally colourless open form (OF) and the more conjugated, coloured closed form (CF).^10–12^ Moreover, they can be designed to respond to other external inputs besides light, thus leading to a multi-responsive behaviour (Fig. 1).^13^ For example, several groups have focused on the acid-induced isomerisation of diarylethenes.^14–18^

Another appealing stimulus is the electrochemical one and redox-responsive dithienylethenes (DTEs) undergoing oxidative^19–23^ or reductive^24–27^ ring-closing or oxidative ring-opening reactions have been reported.^28,29^ However, this input is generally not viable to achieve bidirectional isomerisation in solution, except for a few cases.^30–32^ Focusing on the oxidative cyclisation, thiophenes are generally needed to achieve such redox-active behaviour and their replacement with other electron-poor heterocycles such as thiazoles hinders it.

Only one example in the literature has demonstrated its occurrence in thiazole-containing systems, thanks to the presence of very strong electron-donating substituents.^33^

The investigation towards dual photo- and redox-induced isomerisation has been also performed on terarylenes,^34^ a sub-class of diarylethenes with good photochromic properties (*e.g.* photon-quantitative cyclisation quantum yields),^35,36^ in which a third (hetero)aryl moiety is used in place of the standard central ethene bridging unit (*e.g.* cyclopentene, perfluorocyclopentene, *etc.*). Oxidative cycloreversion of terphenylthiazoles has been extensively investigated by Kawai's group, showing that the OF could be regained much more effectively through the electrochemical route than through the photochemical one, thanks to an oxidative cascade propagation started by the ring-opening of a closed form radical species.^37,38^ Reductive cyclisation of terarylenes bearing *N*-methylpyridinium groups has also been reported.^39,40^ Moreover, bidirectional electrochemical isomerisation has been achieved with terarylenes designed to combine reductive ring-closing and oxidative ring-opening reactions.^39^ Nonetheless, no oxidative cyclisation has been described for this sub-class of derivatives, to our knowledge.

**Fig. 2:**
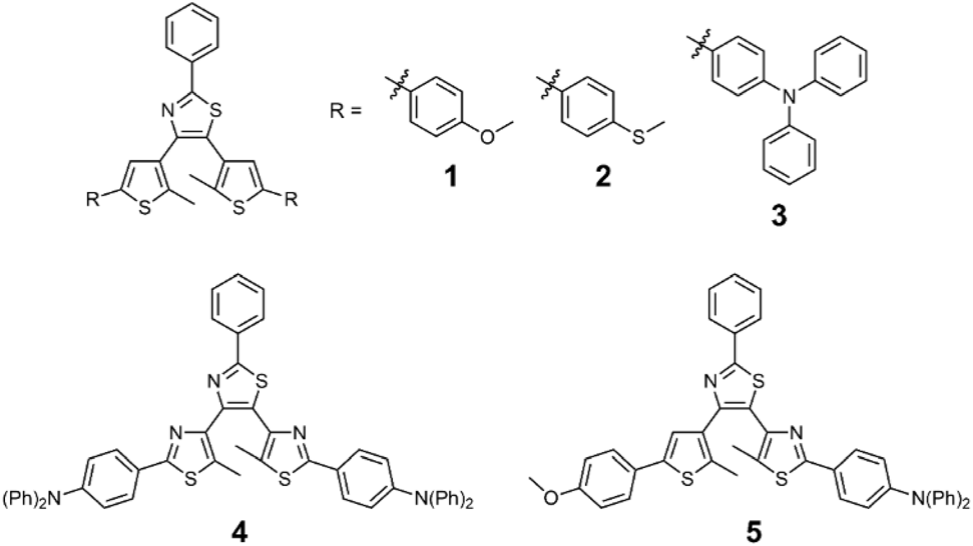
Investigated terarylenes for dual photo- and redox-responsive behaviour.

Inspired by the DTEs characterised by this behaviour described in the literature, we designed terarylenes 1, 2 and 3 depicted in Fig. 2 to develop dual photo- and redox-responsive terarylenes. They bear a central phenylthiazole bridge and two electron-rich thienyl-based arms. Additionally, we prepared terphenylthiazole 4 and the mixed system 5 to investigate if any oxidative cyclisation could be observed in absence or partial replacement of the thiophene rings. Different electron-donating groups were selected and their impact on the electrochemical properties of the switches was evaluated through cyclic voltammetry (CV) and spectroelectrochemistry. While the cyclic voltammograms of the open form isomers of 1, 2 and 3 suggest that an oxidation–reduction sequence affords the neutral closed forms as in the dithienylethene analogues, a different outcome is observed during the bulk electrolysis, with the recovery of the starting neutral open form, thus implying the occurrence of a reversible redox-induced isomerisation between this species and the doubly charged closed form. Moreover, a catalytic and oxidative ring-opening of their neutral closed forms is also achieved. Such unprecedented behaviour is analysed and the rationale behind explained with the support of stopped-flow and EPR measurements, theoretical modelling, and CV simulations. Finally, the observed redox responses of terphenylthiazole 4 and the mixed system 5 clearly indicate that the presence of at least one thiophene-based arm is required to observe the targeted oxidative cyclisation by cyclic voltammetry in these systems.

## Results and discussion

### Synthetic procedures

**Scheme 1:**
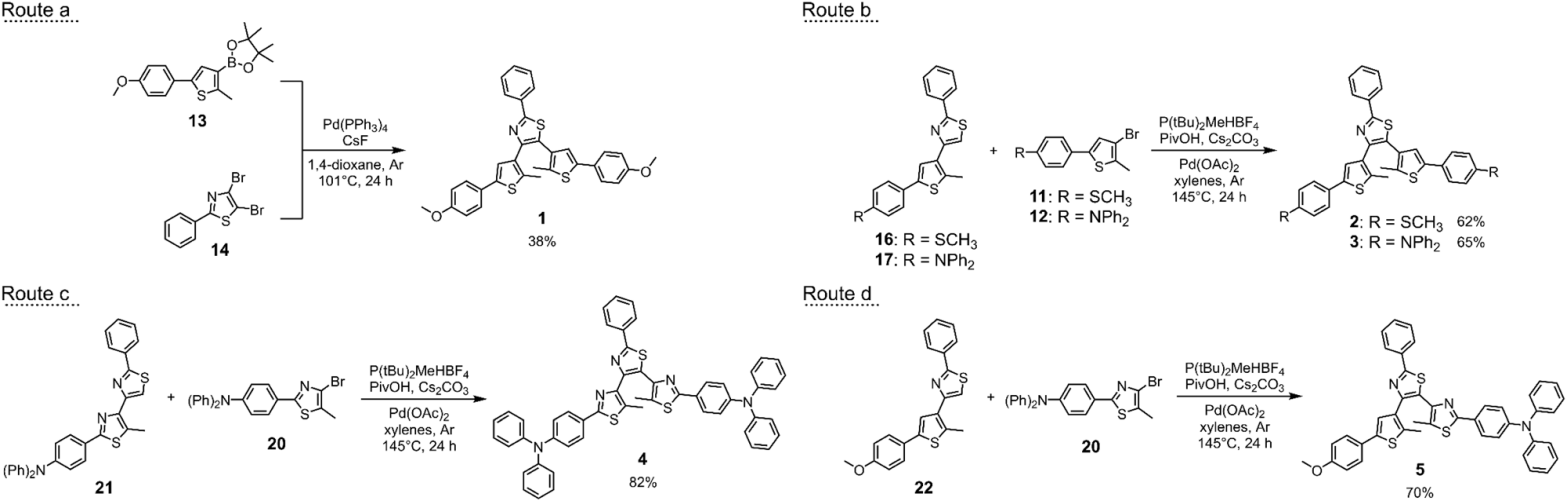
Last steps of the synthetic routes (a, b, c and d) of switches 1–5.

Switches 1, 2 and 3 were obtained by synthesising the thienyl-based arms upon reacting 3,5-dibromo-2-methyl-thiophene (6, Scheme S1†) with the needed boronic acids *via* Suzuki–Miyaura coupling reactions. These intermediates were then used in two different routes (Scheme 1, routes a and b) to afford the target photochromes. Route a involves the synthesis of the boronic acid pinacol ester (13) which is then reacted with 2-phenyl-4,5-di-bromo-thiazole (14) in a double cross-coupling reaction. On the other hand, route b envisages the C–H arylation of intermediates 16 and 17 with the brominated intermediates 11 and 12. Overall, even if both routes are effective for the synthesis of these derivatives, route b is recommendable since it allows to obtain the photochromes in higher yields and it offers higher flexibility as the intermediates 16 and 17 can be potentially used in the synthesis of dissymmetric derivatives, bearing differently functionalised arms.

In the case of photoswitches 4 and 5 (Scheme 1, routes c and d), 2-(4-(diphenylamino)phenyl)-4-bromo-5-methyl-thiazole (20) was used in the direct arylation of intermediates 21 and 22 to afford the desired terphenylthiazole 4 and the dissymmetric terarylene 5. Full details of the synthetic procedures (Scheme S1 and S2) are provided in the ESI,† with the characterisations of all new products.

### Photochromic properties

The photochromic behaviour of 1 was investigated in acetonitrile at room temperature using steady-state absorption spectroscopy, while 2, 3 and 5 were studied in acetonitrile + 2% v/v dichloromethane to guarantee complete solubility. Dichloromethane was used for terphenylthiazole 4 for solubility reasons.

**Fig. 3:**
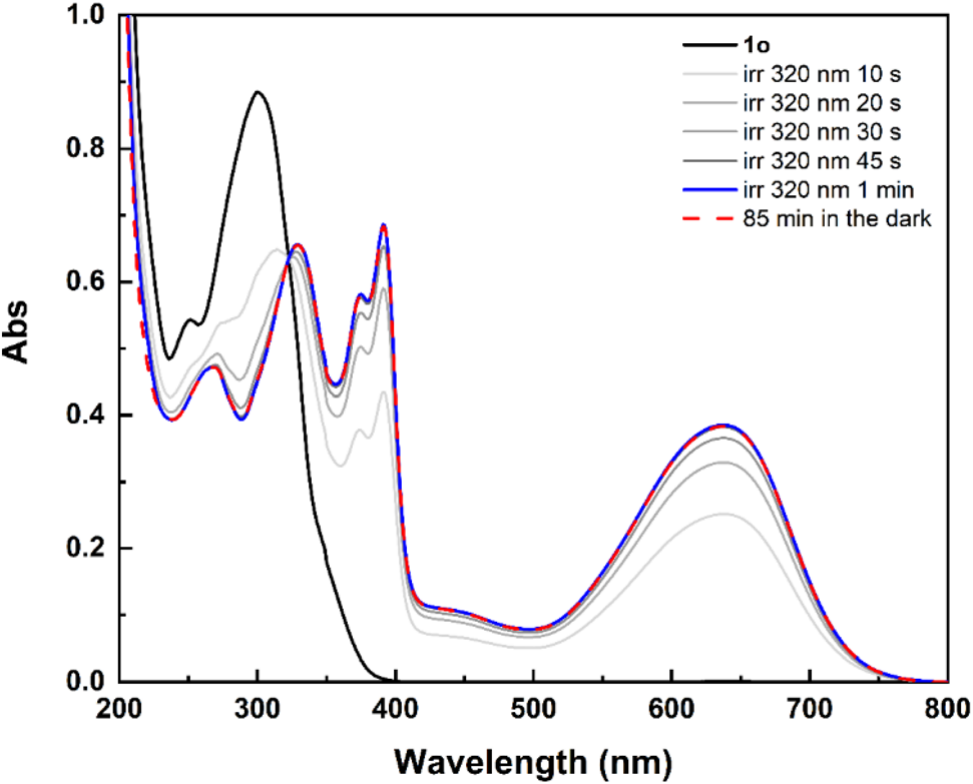
Spectral evolution of 1 (1.84 × 10^−5^ M) in CH_3_CN under UV light irradiation (320 nm) from the open form (1o, black solid line) to the photo-stationary state (blue solid line, *α*_1c_ = 94%). The spectrum recorded after having kept the irradiated solution in the dark at room temperature for 85 min is provided with a red dashed line. Optical path of the cuvette: 1 cm.

The solutions were not degassed before irradiation. The spectral evolution of terarylene 1 in pure acetonitrile under UV irradiation at 320 nm is provided in Fig. 3, where the open form (1o) spectrum is indicated with a black solid line and the achieved photostationary state (*α*_1c_ = 94%) with a blue solid line. A red dashed line is used to show the spectrum recorded after having kept the irradiated solution in the dark at room temperature for 85 minutes and suggests that closed isomer 1c is thermally stable under those conditions.

The open form of terarylene 1 is characterised by an intense absorption band in the UV region, with *λ*_max_ = 300 nm. Under irradiation at 320 nm, a rapid conversion to the closed form is observed, as confirmed by the growth of a broad absorption band peaking at 636 nm and a sharp band at 400 nm, that is accompanied by a shoulder. This reaction is reversible and 1o can be restored upon light irradiation at 600 nm (Fig. S1†).

The spectra for the other photochromes 2–5 are provided in Fig. S2–S5† and show that the OFs absorb in the UV region too, with *λ*_max_ between 300 and 375 nm, and their respective CFs are characterised by broad absorption bands peaking between 600 and 700 nm after UV light irradiation. As in the case of 1c, terarylenes 2c–5c are thermally stable and can be irradiated in the visible to induce the cycloreversion to the corresponding open form isomers.

Moreover, for switches 4 and 5 bearing a 2-(4-(diphenylamino)phenyl)-4-bromo-5-methyl-thiazole arm (20) which is strongly fluorescent (emission spectrum in Fig. S6†) with a quantum yield of 67% in dichloromethane (*λ*_max_ = 462 nm), an emission band at 445 nm was observed for both compounds (emission spectra in Fig. S7 and S8†). The fluorescence quantum yields in CH_2_Cl_2_ for 4 and 5 are 7% and 11%, respectively.

### Electrochemical properties

The electrochemical properties of the five photochromes were investigated by cyclic voltammetry (CV) at room temperature in acetonitrile or dichloromethane/tetrabutylammonium hexafluorophosphate (TBAPF_6_) 0.1 M with a scan rate (*ν*) of 100 mV s^−1^. Dichloromethane was used for 2–5 since no total dissolution could be achieved in acetonitrile.

**Fig. 4:**
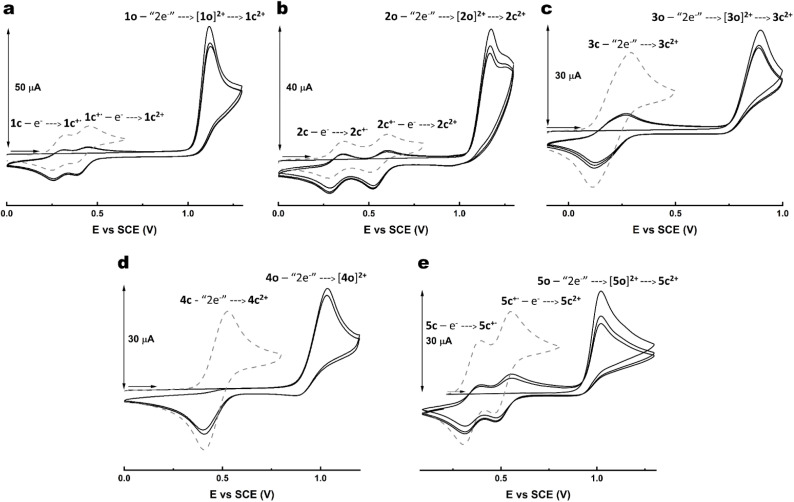
CVs of (a) 1 (1 mM) in CH_3_CN/TBAPF_6_ 0.1 M, (b) 2 (1 mM) in CH_2_Cl_2_/TBAPF_6_ 0.1 M, (c) 3 (1 mM) in CH_2_Cl_2_/TBAPF_6_ 0.1 M, (d) 4 (1 mM) in CH_2_Cl_2_/TBAPF_6_ 0.1 M and (e) 5 (1 mM) in CH_2_Cl_2_/TBAPF_6_ 0.1 M. The CVs of the open form isomers (*i.e.*1o, 2o, 3o, 4o and 5o) are provided with black solid lines. The waves that were observed after partial conversion to the closed form isomers (*i.e.*1c, 2c, 3c, 4c and 5c) by irradiation at 365 nm are shown with grey dashed lines. *ν* = 100 mV s^−1^.

For all the five switches, an irreversible two-electron oxidation wave was detected for the open forms (1o–5o), as it can be observed in Fig. 4. This oxidation occurred at lower potential for 3o when compared to 1o and 2o, as expected from the stronger electron-donating diphenylamino group (Fig. 4c).

Focusing on the cyclic voltammogram of 1, it can be observed that the neutral open form isomer (1o) is irreversibly oxidised at 1.12 V *vs.* SCE. On the back scan, two one-electron cathodic waves appear at *E*_1/2_ = 0.28 V and 0.42 V *vs.* SCE, where *E*_1/2_ is the average potential between anodic and cathodic peak potentials. By comparing these features with those observed in the CV recorded for the photogenerated 1c after irradiation of 1o at 365 nm (Fig. 4a, grey dashed line), it can be concluded that such waves correspond to the two consecutive single-electron reductions of the doubly oxidised closed form (1c^2+^), produced by the oxidation of 1o during the forward scan, leading eventually to the neutral closed form species (1c). This behaviour suggests that redox-active 1 undergoes a relatively fast chemical ring-closing reaction following the electrochemical process, as previously proposed by Feringa and co-workers for similarly functionalised dithienylethenes.^21^

Whether the ring closure occurs only at the radical state (through an ECE mechanism) or at the dicationic state (through an EEC mechanism) is discussed later.

A similar electrochemical behaviour was observed for 2 and 3 (Fig. 4b and c). However, the redox waves of the closed form isomers appeared to be more separated in the case of 2, while almost completely merged for 3. The larger the separation, the more stable the radical species (or the less thermodynamically favourable its dismutation) is. This might be in part explained by the use of dichloromethane as solvent.^21^

A different behaviour was observed for 4o (Fig. 4d). After a two-electron oxidation at around 1 V *vs.* SCE, the back scan shows an irreversible reduction wave at 0.40 V *vs.* SCE which cannot be unequivocally ascribed to the reduction of the dicationic closed form 4c^2+^ since the photogenerated 4c also displays a two-electron redox process at the same potential, but in a reversible manner. Moreover, it is worth noting that this behaviour can be repeated over 25 cycles with no current loss (Fig. S9†). A possible explanation is the impossibility to electrochemically induce the isomerisation at the dicationic state for 4, the electrochemical response corresponding thus only to the reversible, but slow two-electron process. Additionally, multiple-cycle CVs were recorded for 4 after irradiation and showed that 4c could be oxidised to 4c^2+^ and back reversibly (Fig. S10†) while no irreversible wave at 0.40 V *vs.* SCE was detected.

On the contrary, the behaviour of the open form isomer of 5 is similar to 1–3 since it can be irreversibly oxidised at 1.02 V *vs.* SCE leading to two one-electron cathodic waves at *E*_1/2_ = 0.35 V and 0.51 V *vs.* SCE on the back scan, suggesting the reduction of 5c^2+^ to 5c, as confirmed by the CV on the solution irradiated at 365 nm, where the same two redox waves are observed (Fig. 4e). These results prove that the presence of at least one electron-rich thienyl-based arm is necessary for the oxidative cyclisation to occur in the investigated switches. Additionally, as this derivative and its precursor are fluorescent, a potential application in electrofluorochromism can be envisioned (Fig. S11† for a proof-of-concept experiment on precursor 20).^41–45^

The main electrochemical characteristics are summarised in Table 1.

**Table 1:** Redox potentials *vs.* saturated calomel electrode (SCE) in CH_3_CN or CH_2_Cl_2_/TBAPF_6_ 0.1 M of the five investigated switches. For reversible processes: *E*_1/2_ = average potential between anodic and cathodic peak potentials; Δ*E* = anodic to cathodic peak potential difference. For irreversible processes: *E*_p_ = anodic peak potential

	OF		CF	
	*E*_1/2_ or *E*_p_ [V]	Δ*E* [mV]	*E*_1/2_ or *E*_p_ [V]	Δ*E* [mV]
1 a	1.12 (irr)	—	0.28	60
			0.42	60
2 b	1.18 (irr)	—	0.32	80
			0.56	80
3 b	0.90 (irr)	—	0.20	180
4 b	1.03 (—)	—	0.47	120
5 b	1.02 (irr)	—	0.35	90
			0.51	90

a Solvent: acetonitrile.

b Solvent: dichloromethane.

**Fig. 5:**
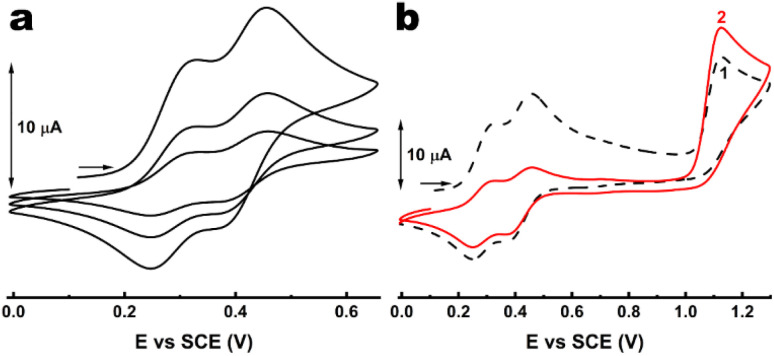
CVs of 1 in CH_3_CN/TBAPF_6_ 0.1 M after partial photoisomerisation through irradiation at 365 nm showing (a) the loss of the closed form isomer in the diffusion layer over three oxidation–reduction cycles and (b) the increase of the open form isomer at expense of the closed form in the diffusion layer when two cycles are recorded up to the potential of the oxidation of 1o. *ν* = 100 mV s^−1^.

Interestingly, unlike their dithienylethene counterparts,^21,22^ our thiophene-containing systems showed an atypical behaviour upon recording multiple-scan CVs with the irradiated solutions. The case of terarylene 1 is depicted in Fig. 5, while the voltammograms for 2, 3 and 5 are provided in Fig. S12–S14.† A loss of the closed form isomer at the vicinity of the electrode surface is found over multiple oxidation–reduction cycles in the potential window of the redox processes of 1c since the peak current is decreasing at each iteration (Fig. 5a).

Besides, when the CV is recorded over the whole potential window (Fig. 5b), an increase of the peak current corresponding to 1o oxidation is observed, meaning its local concentration also increases. The two above observations suggest the occurrence of an electro-activated cycloreversion from 1c to 1o close to the electrode. This behaviour, if confirmed, would be the first experimental observation of coexisting oxidative ring-closing and ring-opening reactions.

This unanticipated feature was further investigated by recording CVs for 1o and 1c at different scan rates and the obtained voltammograms are presented in Fig. S15.† Concerning 1o (Fig. S15,† left), the two-electron oxidation remained unambiguously irreversible while increasing the scan rate from 25 mV s^−1^ to 4 V s^−1^ (limit of our equipment), suggesting a relatively large rate constant for the chemical step leading to ring closure. In the case of the DTE counterpart of 1, Feringa and co-workers reported that the irreversible signature was observed up to 1000 V s^−1^, corresponding to a ring-closure kinetic constant greater than 10^4^ s^−1^. While it would be reasonable to assume comparable rates for our investigated thienyl-based terarylenes, the lower limit for ring closure would rather be 10^2^ s^−1^ if ring closure occurs at the dicationic state (EEC mechanism, *vide infra* and in the ESI†).

In contrast, a significant impact could be observed on the two waves related to the closed form isomer at faster scan rates, suggesting an improved reversibility of the corresponding electrochemical processes. This indicates that at higher scan rates (*i.e.* short measurement times), the above mentioned electro-activated cycloreversion from 1c to 1o barely has time to occur. This is confirmed with CVs after partial conversion to the photogenerated closed form (Fig. S15,† right), where the disappearance of the CF species in the diffusion layer was significant at a slow scan rate such as 25 mV s^−1^, while it was almost prevented at *ν* ≥ 1 V s^−1^.

### Spectroelectrochemical studies

**Fig. 6:**
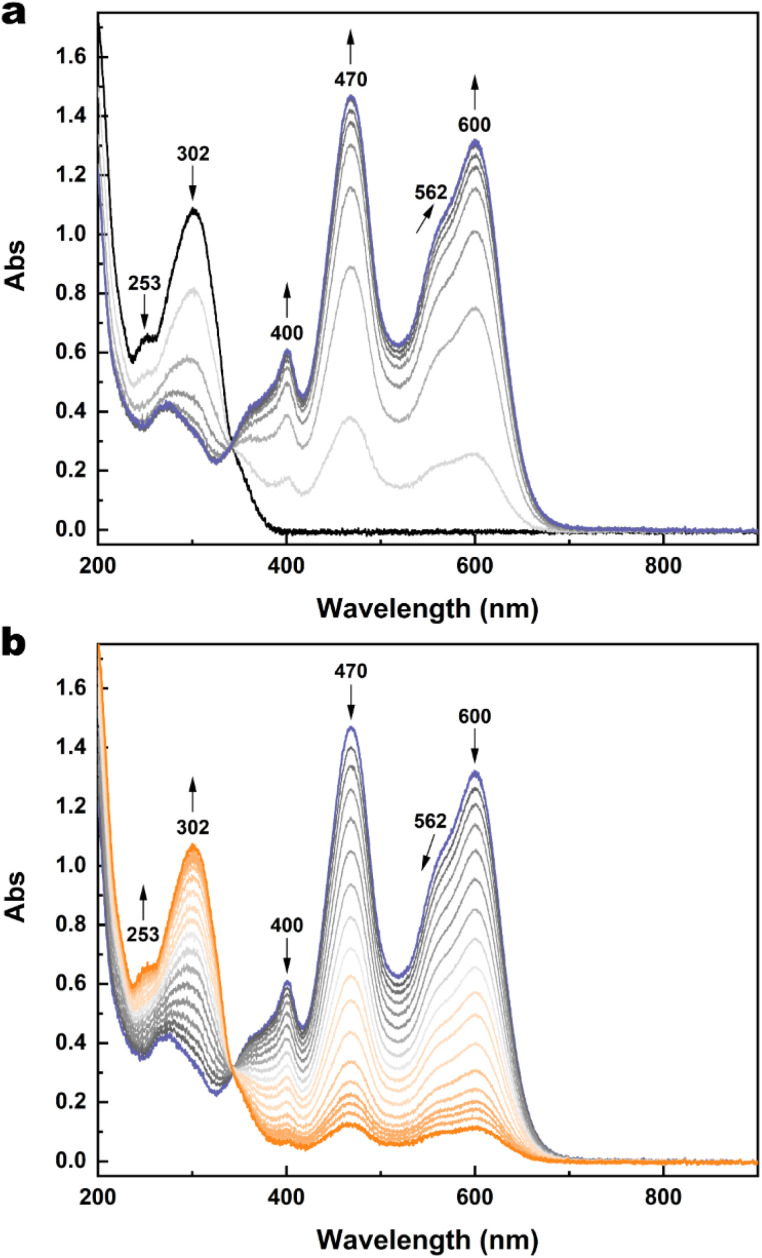
(a) Spectral evolution of 1o (2.27 × 10^−4^ M) in CH_3_CN/TBAPF_6_ 0.1 M under oxidation at 1.30 V at RT. (b) Spectral evolution of 1c^2+^ (2.27 × 10^−4^ M) in CH_3_CN/TBAPF_6_ 0.1 M under reduction at 0.50 V at RT. Optical path of the cuvette: 1 mm. For both (a) and (b), 1 spectrum/minute was recorded, but in the case of (b), a selection of spectra is provided for the sake of clarity.

To better elucidate the redox-triggered processes of 1 and possibly identifying the transient species involved in its isomerisation under an electrochemical stimulus, spectroelectrochemical experiments at room temperature were carried out in acetonitrile/TBAPF_6_ 0.1 M. A solution of 1o was initially electrolysed at 1.30 V *vs.* SCE. The induced spectral evolution was followed by UV-vis spectroscopy (Fig. 6a).

Because of the oxidation, the bands at 253 nm and 302 nm decreased while three new bands grew at 400 nm, 470 nm, and 600 nm and remained quite stable after the end of the oxidation. These new spectral features are attributed to the dicationic species 1c^2+^. The formation of this species after the oxidation is so fast that neither the monocationic radicals (either in the open or the closed form) nor 1o^2+^ are observed as intermediate species during the oxidation.

Then, a second electrolysis was carried out at 0.50 V, that is the potential at which the wave for the one-electron reduction 1c^2+^ → 1c^+^˙ starts, thus attempting to obtain an optical signature of the radical species. Instead, a return to the open form is observed (Fig. 6b).

Similar spectral evolutions were observed also for 2, 3 and 5 and the corresponding spectroelectrochemical experiments are provided in Fig. S17–S26.† This result suggests a possible ring-opening reaction at the radical stage. If happening, 1o^+^˙ generated from cycloreversion would be rapidly reduced to 1o while maintaining the electrode potential at 0.50 V during the electrolysis, since it is electrochemically generated from 1o at much higher potential (at least 1.12 V). In addition, a full return to 1o can be achieved by further lowering the potential to 0.20 V (Fig. S16†), indicating that 1o can be switched to 1c^2+^ and then reobtained through an oxidation–reduction sequence.

### Possible mechanism ruling the bidirectional dual-responsive isomerisation

Terarylene 1 was chosen to investigate the underlying mechanism of such bidirectional behaviour. Additionally, given their qualitatively similar redox behaviour, the presented mechanism should be valid also for 2, 3 and 5.

Since the photo-generated neutral 1c is thermally stable at room temperature, the ring-opening reaction might occur either at the dicationic redox state,^46,47^ or at the radical one.

First, the thermal stability of the dicationic species 1c^2+^ was qualitatively evaluated by oxidising a solution of 1o in acetonitrile with two equivalents of tris(4-bromophenyl)ammoniumyl hexachloroantimonate (also known as “magic blue”, 
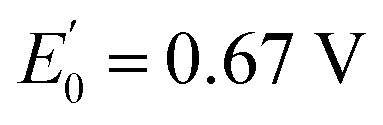
*vs.* Fc in acetonitrile).^48^ The obtained spectrum is shown in Fig. S27.† The solution was kept in the dark at room temperature for 1 h and, as only a slight spectral variation was detected, 1c^2+^ can be considered relatively stable. Consequently, we postulate that the thermal ring-opening is more probably occurring at the radical redox state (see also the “Theoretical modelling” section below), according to the following chemical equilibrium (eqn (1)):

1 1c^+^˙ ⇆ 1o^+^˙

Stopped-flow experiments were carried out to investigate this cycloreversion at the radical state.

**Fig. 7:**
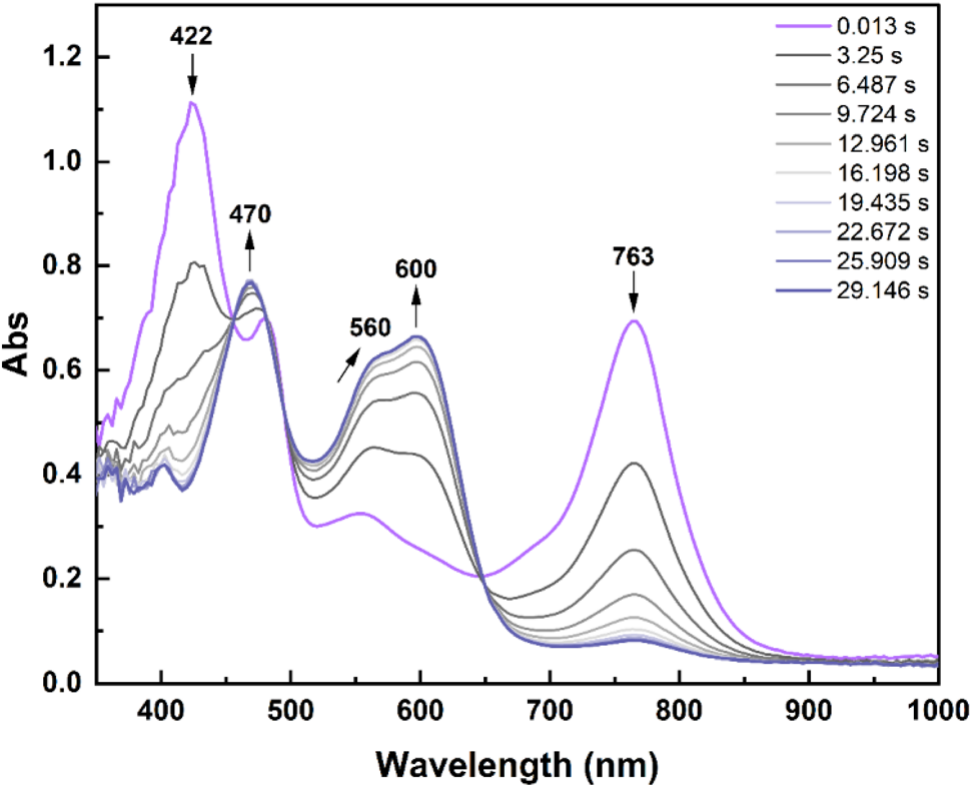
Stopped-flow absorption spectral evolution upon reduction of a 1c^2+^ solution (2.27 × 10^−5^ M) with a Me_10_Fc solution (2.27 × 10^−5^ M) in CH_3_CN. Mixing time: 10 ms. Optical path: 1 cm.

Upon reducing a solution of 1c^2+^ (previously prepared by electrolysing a solution of 1o at 1.2 V *vs.* SCE, details in the “Stopped-flow measurements and chemical red/ox experiments” section of the ESI, Fig. S28†) with 1 eq of decamethylferrocene (Me_10_Fc, 
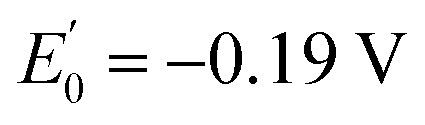
*vs.* SCE in acetonitrile),^48^ no bands of the dicationic species could be observed after the mixing time (10 ms), but a band at 422 nm and one at 763 nm were detected (Fig. 7) and attributed to 1c^+^˙.

The disappearance of the radical bands was accompanied by a concurrent growth of the bands of 1c^2+^ with isosbestic points at 453 nm, 497 nm and 651 nm. This is ascribed to the cross-dismutation between the newly formed 1o^+^˙and a neighbour 1c^+^˙ molecule, as in eqn (2), which leads to a recovery of 50% of the initial concentration of 1c^2+^.

2 1o^+^˙ + 1c^+^˙ → 1o + 1c^2+^

By monitoring the disappearance of the band at 763 nm over time (*ca.* 30 s), an *apparent* kinetic constant of 0.2 s^−1^ was determined for the cycloreversion of 1c^+^˙, through a monoexponential fit (Fig. S29b†). It is worth noting that this value is an overestimation since 1o^+^˙, once generated, is consumed quickly according to eqn (2) given the large oxidation potential difference between the two species, further displacing eqn (1). Such an order of magnitude for the kinetic constant is consistent with what was determined by Kawai and co-workers in their investigation of the ring-opening reaction of radical closed-ring terarylenes.^37^

The same spectral evolution was observed when such chemical reduction conditions were applied in stationary UV-vis experiments, confirming the formation of a 1 : 1 1o/1c^2+^ solution after the reduction of 1c^2+^ with 1 eq of Me_10_Fc (Fig. S30†).

**Fig. 8:**
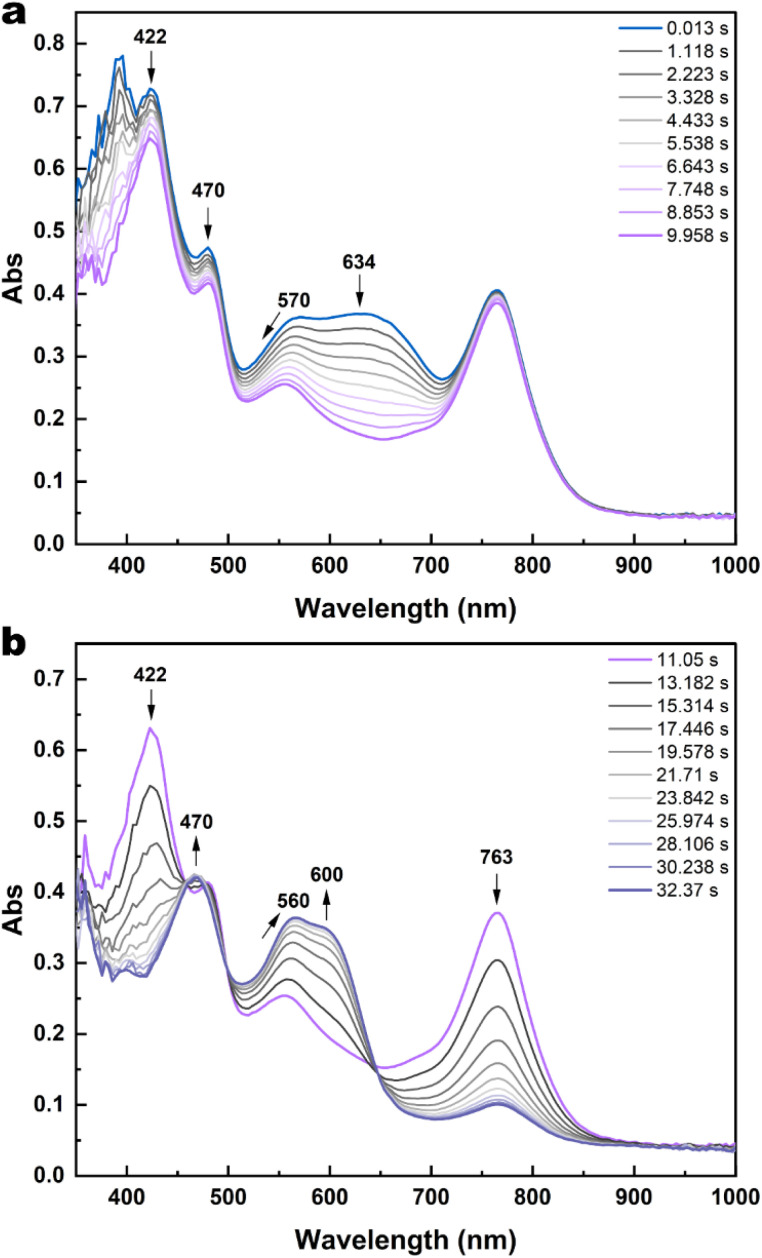
Stopped-flow absorption spectral evolution upon reduction of a 1c^2+^ solution (2.27 × 10^−5^ M) with a Me_10_Fc solution (4 × 10^−5^ M) in CH_3_CN (a) during the first 10 s and (b) between 11 s and 32 s. Mixing time: 10 ms. Optical path: 1 cm.

Next, stopped-flow monitoring of the reduction of 1c^2+^ with 1.8 eq of decamethylferrocene was performed. Upon using such a larger amount of chemical reductant, the formation of 1c in addition to 1c^+^˙ could be detected after the mixing time (Fig. 8a). The presence of the neutral closed form isomer allowed the occurrence of the cascade mechanism proposed by Kawai's group^37^ (eqn (3)), where 1c^+^˙ acts as a “catalyst” of the ring-opening of 1c towards 1o. This is suggested by the decrease of the band of 1c between 600 nm and 700 nm, while the band of 1c^+^˙ at 763 nm remained stationary until the total consumption of the neutral closed form (*ca.* 10 s, Fig. 8b) before disappearing with an *apparent* kinetic constant of 0.18 s^−1^ (Fig. S31b†).

3 1o^+^˙ + 1c → 1o + 1c^+^˙

The occurrence of this bimolecular reaction was further confirmed by stationary UV-vis spectroscopy (Fig. S32†). A fast return to 1o was observed upon addition of a catalytic amount (0.10 eq) of ferrocenium tetrafluoroborate (freshly prepared solution in acetonitrile, under Ar; 
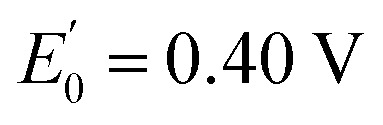
*vs.* SCE in acetonitrile)^48^ to a non-degassed solution of 1 at the photostationary state (*α*_1c_ = 94%) in acetonitrile at room temperature. One spectrum every 0.4 min was recorded after the addition of such sub-stoichiometric amount of oxidant and ≥90% of 1c was reopened to 1o in 2 min *ca.* (orange dashed-dotted line in Fig. S31†).

Two radical species being involved, the disproportionation of 1c^+^˙ (eqn (4)) and 1o^+^˙ (eqn (5), where 1o^2+^ would then spontaneously cyclise to afford 1c^2+^) should be also considered to give a more complete overview of all the possible reactions that could occur.^47^

4 2 1c^+^˙ ⇆ 1c + 1c^2+^

5 2 1o^+^˙ ⇆ 1o + 1o^2+^

Note that the disproportionation of 1c^+^˙ (eqn (4)) is not thermodynamically favourable as the electrochemical behaviour of 1c shows two successive single-electron processes (as discussed above), but the reverse reaction (*i.e.* comproportionation) is, and this has an impact on the CV response (see “Cyclic voltammetry simulation” section in the ESI†).

The dismutation of 1o^+^˙ (eqn (5)) is likely an unfavourable competitive reaction of the cross-dismutation (eqn (2)) or the oxidative cascade ring-opening of 1c (eqn (3)) in most experimental conditions described here and therefore it will not be explicitly considered in the next sections since 1o^+^˙ is poorly accumulated, unlike 1c^+^˙, thus making this reaction statistically less probable.

**Fig. 9:**
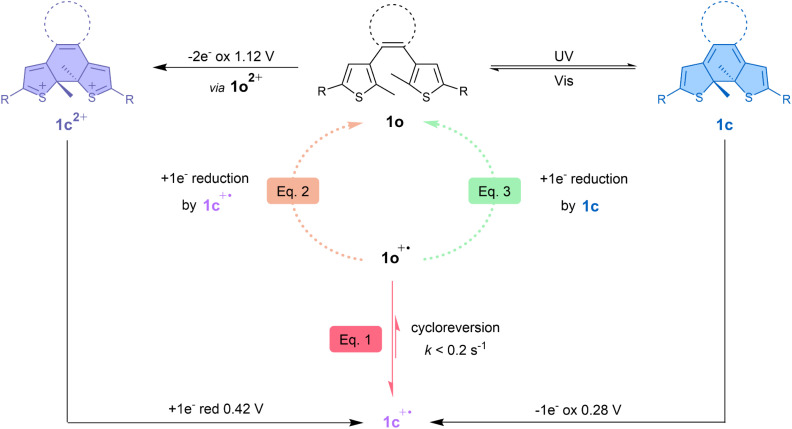
Mechanism summarising the bidirectional photochemical and electrochemical reactions of terarylene 1 involving its open form (1o), closed form (1c) and dicationic closed form (1c^2+^). The cycloreversion at the radical state (eqn (1)) is indicated with red arrows. The bimolecular reaction between 1o^+^˙ and 1c^+^˙ (eqn (2)) is graphically shown with an orange dotted arrow and the cascade reaction proposed by Kawai's group^37^ between 1o^+^˙ and 1c (eqn (3)) is depicted with a light green dotted arrow. R = *p*-OMe-Ph.

Based on the different results obtained, the overall bidirectional dual-responsive behaviour of 1o is summarised in Fig. 9 and highlights the versatile nature of such a molecular switch which has reversible access to two different closed form isomers depending on the chosen stimulus. The neutral open form can reversibly isomerise towards the neutral closed form (1c) upon light irradiation and switch to the dicationic closed form (1c^2+^) upon oxidation-induced cyclisation. The generation of the intermediate radical species 1c^+^˙ allows to restore 1o through cycloreversion at the radical state, meaning that 1o and 1c^2+^ can thus be interconverted by reversible isomerisation through an oxidation–reduction sequence and that 1o can also be restored upon one-electron oxidation of 1c.

To further support the proposed mechanism, coupled electrolysis-EPR spectroscopy of 1o (2.5 mM) in CH_2_Cl_2_/TBAPF_6_ 0.24 M was carried out with an *in house* three-electrode setup placed inside an EPR tube (details in the ESI† and its “Coupled electrolysis-EPR spectroscopy” section). Once determined the potentials to use through cyclic voltammetry (Fig. S33†), EPR spectra were recorded before electrolysis (*i.e.* for 1o), during an oxidation at 1.3 V (*i.e.* for 1c^2+^) and during a reduction at 0.5 V (Fig. S34†). While no signal was observed for 1o and 1c^2+^, a non-persistent organic radical (*g* = 2.0037) was detected on the first scan during the electrolysis at 0.5 V, thus providing evidence of the generation of a radical species (*i.e.*1c^+^˙). The fact that the radical could not be accumulated is in agreement with the occurrence of the ring-opening reaction at the radical state (eqn (1)) and the ensuing consumption of 1o^+^˙ during the experiment.

Having experimentally determined the *apparent* kinetic constant for the ring-opening of 1c^+^˙, cyclic voltammetry simulations as well as simulations of the time traces of the stopped-flow experiments were carried out to support the proposed mechanism (details in the ESI,† “Cyclic voltammetry simulations” and “Time traces simulations” sections, respectively) and estimate the equilibrium constant of such cycloreversion at the radical state (eqn (1)) and the rate constants of the bimolecular reactions (eqn (2) and eqn (3)).

The disproportionation of 1c^+^˙ (eqn (4)) was included in both the time traces and CV models. As mentioned above, the thermodynamic equilibrium constant for this reaction is imposed by the Δ*E* between the two consecutive one-electron transfers involving the closed form and it is equal to 0.0043. The rate constants for disproportionation and comproportionation (eqn (4)) were estimated by cyclic voltammetry simulations to be at least greater than 10^3^ s^−1^ M^−1^ and 2.32 × 10^5^ s^−1^ M^−1^, respectively, while keeping their ratio fixed to 0.0043. Note that only a lower possible value of the rate constants may be estimated since above a certain value the contribution to the CV simulation is negligible, probably due to the fact that the reaction becomes too fast so that the process is limited by the diffusion rate of 1c and 1c^2+^. These values were used in the time traces simulations (*vide infra* and the corresponding sections in the ESI†).

The simulations of the CVs of 1 at *ν* = 100 mV s^−1^ were performed with DigiElch8 (“Cyclic voltammetry simulations” section in the ESI†). Concerning the simulation of the cyclic voltammogram of 1o (Table S1†), the formation of 1c^2+^ was ascribed to two concerted one-electron oxidations of 1o leading to spontaneous ring-closing reaction at the dicationic state with a kinetic constant *k* of at least 100 s^−1^ in accordance with the literature.^21^ Attempts to model this electrochemical reaction through an ECE mechanism (*i.e.* one-electron oxidation of 1o to 1o^+^˙ which spontaneously cyclises to 1c^+^˙ and then is further oxidised to 1c^2+^) showed that improved reversibility should have been observed at faster scan rates (which is not the case experimentally), thus supporting the proposition of two simultaneous single-electron transfers (EEC mechanism).^33^ Regarding the ring-opening of 1c^+^˙ leading to 1o^+^˙, a kinetic constant (*k*_b_) equal to 0.1 s^−1^ was used. For the bimolecular reactions between 1o^+^˙ and either 1c^+^˙ (eqn (2), *vide supra*) or 1c (eqn (3), *vide supra*), irreversibility was assumed and rate constants of at least 5 × 10^6^ s^−1^ M^−1^ and 9 × 10^6^ s^−1^ M^−1^, respectively, were considered. A larger constant for the reaction between 1o^+^˙ and 1c (eqn (3)) compared to that for the reaction between 1o^+^˙ and 1c^+^˙ (eqn (2)) is coherent with the neutral closed form species 1c being a better reductant than the closed form radical 1c^+^˙.

The simulated CV is depicted in Fig. S36,† where *K*_eq_ for 1o^+^˙ ⇆ 1c^+^˙ is set to 20, and the inclusion of the disproportionation of 1c^+^˙ (eqn (4), *vide supra*, and light orange row in Tables S1 and S2†) is needed to obtain a satisfying fit. This is even more relevant in the case of the modelling of the CVs for 1c (Table S2 and Fig. S37†) since there is coexistence of 1c and 1c^2+^ during the experiment. Indeed, once 1c^2+^ diffuses towards the solution after being formed upon oxidation at the vicinity of the electrode, it can react with 1c diffusing from the bulk of the solution towards the electrode. This comproportionation reaction can lead to an accumulation of 1c^+^˙ far from the electrode, which then reopens into 1o^+^˙, itself being reduced either by 1c (most probably) or by another 1c^+^˙ (cross-dismutation reaction) eventually affording 1o, thus contributing significantly to the electrochemically-induced cycloreversion.

A further refinement of the modelling for 1o by including a parasitic process consuming 1c^2+^ due to degradation of redox-active DAEs under the electrochemical conditions^21,33^ is discussed in the ESI.†

Regarding the CVs of the photo-generated 1c at *ν* = 100 mV s^−1^ (Fig. S37†), the disappearance of the waves over multiple cycles could be modelled with the above-mentioned values, further confirming the validity of the rate constants for the processes involved and the overall mechanism.

Finally, CVs were also modelled for different scan rates and the experimental trends were reproduced (Fig. S38 and S39†).

The simulation (Fig. S40†) of the time trace for the decay of the band at 763 nm obtained upon reduction of 1c^2+^ with 1 eq of Me_10_Fc (Fig. S29†) was carried out using Kintek Explorer.^49,50^ It was assumed that the ring-opening at the radical state (eqn (1)), the cross-dismutation between 1o^+^˙ and 1c^+^˙ (eqn (2)), the oxidative ring-opening of 1c (eqn (3)) and the disproportionation of 1c^+^˙ (eqn (4)) were involved. The shape of the trace was best reproduced with *k* = 0.122 s^−1^ for 1c^+^˙→1o^+^˙, *k* = 2.19 s^−1^ for 1o^+^˙→1c^+^˙, *k* = 5.68 × 10^6^ s^−1^ M^−1^ for 1o^+^˙ + 1c^+^˙ → 1o + 1c^2+^, and 8.09 × 10^7^ s^−1^ M^−1^ for 1o^+^˙ + 1c → 1o + 1c^+^˙ which are values in good accordance with the CV simulations.

Next, the time traces for the evolution of the bands at 600 nm and 763 nm observed when 1c^2+^ was reduced with 1.8 eq of Me_10_Fc (Fig. S31†) were simulated (Fig. S41 and S42†). Satisfyingly, the same rate constants led to an acceptable match between simulated and experimental time traces at such wavelengths, further validating their estimation.

### Theoretical modelling

To better rationalise and reinforce the proposed mechanism, DFT calculations were carried out on the five investigated terarylenes (computational details provided in the ESI†). The geometries of the open and closed forms of 1–5 were fully optimised at the ωB97XD/6-311G(d,p) level of calculations in acetonitrile in the different redox states 0, +1 and +2. Unless otherwise stated, the antiparallel open form is considered since that is the conformer that can undergo ring closure reaction upon irradiation according to the Woodward–Hoffmann rules.^51^ The relative energies of the CFs with respect to OFs are presented in Table 2.

**Table 2:** Relative energies (in kJ mol^−1^) of the closed form isomers of 1–5 in neutral, radical and dicationic states at the ωB97X-D/6-311G(d,p) level of calculations. The values are calculated with respect to the energies of the antiparallel open form isomers in such redox states (0 kJ mol^−1^ for the sake of comparison)

Redox state	1	2	3	4	5
**+0**					
Δ*E*_CF–OF_	78.8	80.8	78.9	70.5	73.3

**+1**					
Δ*E*_CF–OF_	−22.1	−15.1	5.7	−29.6	0.5

**+2**					
Δ*E*_CF–OF_	−100.2^a^	−170.7^a^	−130.7	−127.5	−140.3

a The antiparallel geometries of 1 and 2 in the dicationic open form state couldn't be obtained since the optimisation converged to the corresponding dicationic closed forms. The relative energies of the closed forms were then calculated from the parallel conformation of the open forms.

As expected, all the five antiparallel open forms in neutral state are more stable than the corresponding closed forms, and the energy differences between the two isomers are not significantly affected by the different electron-donating nature of the substituents in this redox state.

The computed energy difference is in the range of 70.5 kJ mol^−1^ for 4 to 80.8 kJ mol^−1^ for compound 2. This is well in line with the aromaticity change of thienyl or thiazolyl arms from OF and CF isomers as previously proposed by Nakamura.^52^ The destabilisation due to the loss of aromaticity is calculated to be between 60 and 65 kJ mol^−1^ (Table S3†). In stark contrast, the relative stability of the two forms is dramatically reversed in the dicationic redox state, with the CF being stabilised by more than 100 kJ mol^−1^ for all the members of the series.

An intermediate situation is found in the radical redox state, with much smaller energy differences between the two isomers. These computational results are in good agreement with the observed experimental data, *i.e.* the two-electron oxidation-induced cyclisation of 1o–3o and 5o, the thermal stability of the corresponding CF^2+^, and are also in favour of a ring-opening mechanism of the CF at the radical redox state rather than at the dicationic one.

Additionally, the same calculations were also carried out on the DTE analogue of 1 investigated by Feringa and co-workers (“Additional calculation data” section in the ESI†). As expected, the neutral closed form isomer of the DTE is more stable than 1c (Table S4,† 37.3 kJ mol^−1^ for DTEc*vs.* 78.8 kJ mol^−1^) and the dicationic closed form isomers have similar relative stabilities (Table S4,† −109.6 kJ mol^−1^ for DTEc^2+^*vs.* −100.2 kJ mol^−1^), in accordance with the oxidative cyclisation that both systems show. However, in the case of the radical state, 1c^+^˙ is considerably less stable than its DTE radical counterpart (Table S4,† −22.1 kJ mol^−1^*vs.* −46.9 kJ mol^−1^ for DTEc^+^˙), suggesting that the ring-opening is more favourable in our systems and thus supporting the proposed mechanism.

**Fig. 10:**
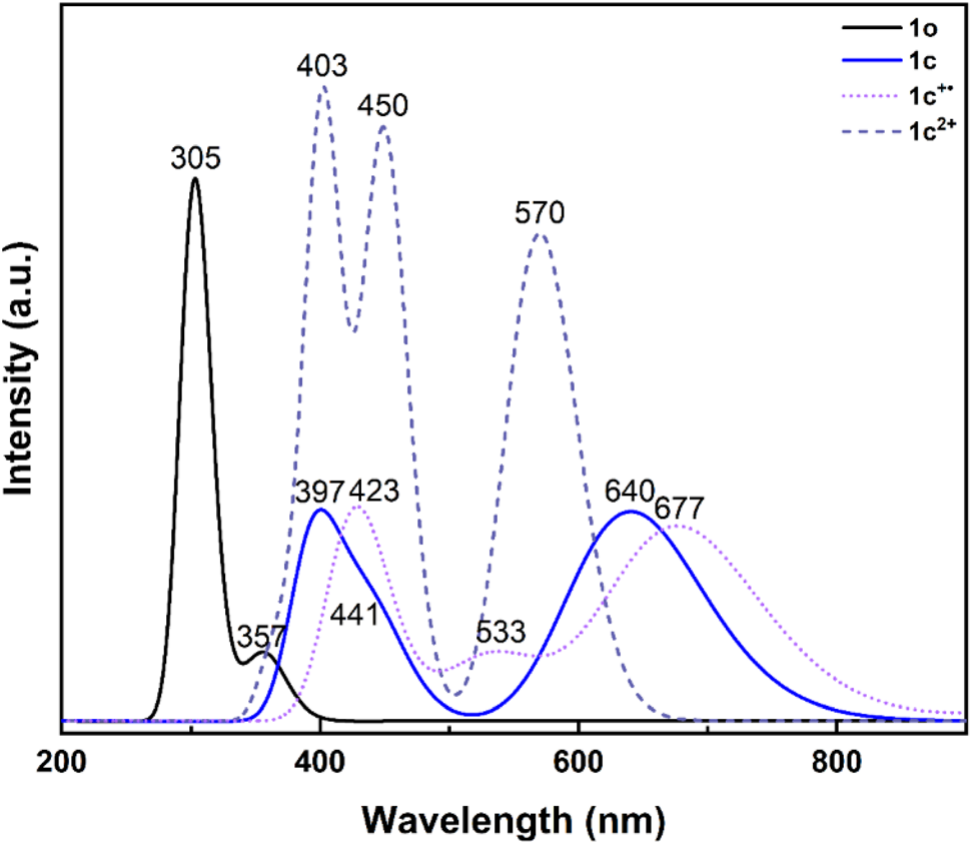
Calculated absorption spectra of 1o (black solid line), 1c (blue solid line), 1c^+^˙ (light violet dotted line) and 1c^2+^ (purple dashed line).

TD-DFT calculations were also performed to simulate the optical properties of 1o, 1c, 1c^+^˙ and 1c^2+^ (Fig. 10). The calculated spectra for the neutral open form and closed form of 1 are in very good agreement with the experimental findings. For 1o, a weak transition at 357 nm (S_0_ → S_1_) that appears as a shoulder in the experimental spectrum and two strong transitions at 305 nm (S_0_ → S_3_) and 301 nm (S_0_ → S_4_) were predicted. Interestingly, these two transitions promote an electron towards the LUMO and LUMO+1 orbitals, respectively. Careful analyses indicate that these virtual orbitals exhibit a significant density on the two reactive carbon atoms and a bonding character for the to-be-formed C–C bond (Fig. S43†).

The LUMO+1 being strongly bonding between the reactive carbons can be labelled as “photochromic” since it triggers the ring closure reaction.^53,54^ The analysis of the HOMO−1 orbital of 1o is also interesting since it can be anticipated that this orbital becomes the HOMO for 1o^2+^. HOMO−1 shows a strong bonding character between the reactive carbon atoms and exhibits “thermochromic” topology for the dicationic state. Indeed, this can explain a spontaneous ring closure of the open form in the dicationic state and why no antiparallel form could be optimised. Similar behaviour for HOMO−1 is observed for 2o^2+^ while less strong bonding character is observed for 3o^2+^, 4o^2+^ and 5o^2+^ (Fig. S44†).

As for 1c, the calculations predict two strong transitions at 640 nm (S_0_ → S_1_) and 397 nm (S_0_ → S_3_), which compare well with the 636 nm and 400 nm experimental bands. As observed experimentally, a shoulder is also calculated at 441 nm (S_0_ → S_2_).

In the calculated UV-vis spectrum of 1c^+^˙, the energy of the lowest-energy visible band is overestimated (677 nm *vs.* 763 nm, absolute error of 0.21 eV, S_0_ → S_2_), but overall, compares well with the experimental spectrum obtained during the stopped-flow measurements by also predicting a transition at 423 nm (S_0_ → S_10_, *λ*_exp_: 423 nm) and one at 533 nm (S_0_ → S_4_, *λ*_exp_: 557 nm), shoring up the attribution of such spectrum to the formation of 1c^+^˙.

Last, in the case of 1c^2+^, the theoretical spectrum is dominated by three electronic transitions at 570 nm, 450 nm, and 403 nm in good agreement with the experimental values (*λ*_exp_: 600, 470 nm and 400 nm), thus supporting the formation of such species upon two-electron oxidation of 1o.

## Conclusions

In summary, three terarylenes bearing electron-rich thienyl-based arms, one terphenylthiazole and one mixed dissymmetric system have been synthesised and their photo- and redox-active behaviours investigated. These terarylenes (except for terphenylthiazole-based 4) were found to display both oxidative ring-closing of their open forms and oxidative catalytic ring-opening of their closed forms, besides the expected photoswitching. These redox-active processes have been reported for dithienylethenes, but not their co-occurrence within the same molecule. Moreover, despite sharing similar CV features with their known DTE analogues, oxidation–reduction electrolysis sequences on 1o, 2o, 3o and 5o do not afford the corresponding neutral closed forms. A return to the neutral open forms is observed instead. In other words, this family of switches can be reversibly operated between two different closed form isomers depending on the chosen stimulus. With the support of CV, stopped-flow and EPR experiments as well as by DFT modelling, kinetics and cyclic voltammetry simulations, the observed bidirectional photo- and redox-induced multi-state isomerisation is rationalised. This unprecedented behaviour is ascribed to the combination of the features compatible with the largely thermodynamically-driven oxidative cyclisation like in analogous DTEs (*i.e.* electron-rich thiophene), and a comparatively less stabilised closed form radical giving rise to an equilibrium with its corresponding open form counterpart. Albeit thermodynamically unfavoured, the ring-opening at the radical state is strongly accelerated by the thermodynamically favourable and fast consumption of the open form radical in bimolecular redox reactions either (1) with a neighbouring closed form radical or (2) with a neutral closed form molecule. Such rich redox-switching behaviour, along with easier structural modifications show the potential of terarylenes for the development of advanced photo- and redox-responsive molecular switches.

## Author contributions

N. B. and P. Y. performed the synthesis of the investigated switches. N. B. carried out the UV-vis spectroscopy and electrochemistry experiments. A. L. assisted N. B. in the spectroelectrochemical measurements. J.-N. R. carried out the stopped flow experiments and the time traces simulations. F. Mav. supported N. B. in conducting the cyclic voltammetry simulations. E. R. and N. B. realised the electrofluorochromism experiment. C. H. carried out the coupled electrolysis-EPR experiment. F. Mau. performed all the theoretical modelling. N. B., A. L., J.-N. R., F. Mav. and P. Y. discussed together to rationalise the redox-active behaviour of the investigated switches. A. L. and P. Y. supervised N. B. during the whole project. N. B., A. L. and P. Y. designed the project. P. Y. and F. Mau. were responsible for funding acquisition. N. B. prepared the original draft. All the authors contributed to the review and editing of the original draft.

## Conflicts of interest

There are no conflicts to declare.

## Supplementary Material

SC-016-D5SC02845K-s001

## Data Availability

The data supporting this article have been included as part of the ESI.†

## References

[cit1] Andréasson J., Pischel U., Straight S. D., Moore T. A., Moore A. L., Gust D. (2011). J. Am. Chem. Soc..

[cit2] Pu S., Tong Z., Liu G., Wang R. (2013). J. Mater. Chem. C.

[cit3] Roldan D., Cobo S., Lafolet F., Vilà N., Bochot C., Bucher C., Saint-Aman E., Boggio-Pasqua M., Garavelli M., Royal G. (2015). Chem.–Eur. J..

[cit4] Zhao F., Grubert L., Hecht S., Bléger D. (2017). Chem. Commun..

[cit5] Heindl A. H., Becker J., Wegner H. A. (2019). Chem. Sci..

[cit6] Nie H., Self J. L., Kuenstler A. S., Hayward R. C., Read de Alaniz J. (2019). Adv. Opt. Mater..

[cit7] Stricker F., Sanchez D. M., Raucci U., Dolinski N. D., Zayas M. S., Meisner J., Hawker C. J., Martínez T. J., de Alaniz J. R. (2022). Nat. Chem..

[cit8] Zitzmann M., Hampel F., Dube H. (2023). Chem. Sci..

[cit9] Lv Y., Ye H., You L. (2024). Chem. Sci..

[cit10] Irie M. (2000). Chem. Rev..

[cit11] Irie M., Fukaminato T., Matsuda K., Kobatake S. (2014). Chem. Rev..

[cit12] Irie M. , Diarylethene Molecular Photoswitches: Concepts and Functionalities, Wiley-VCH, Weinheim, 2021

[cit13] Ai Q., Lan K., Li L., Liu Z., Hu X. (2024). Adv. Sci..

[cit14] Deng X., Liebeskind L. S. (2001). J. Am. Chem. Soc..

[cit15] Kobatake S., Terakawa Y. (2007). Chem. Commun..

[cit16] Nakashima T., Miyamura K., Sakai T., Kawai T. (2009). Chem.–Eur. J..

[cit17] Kutsunugi Y., Coudret C., Micheau J. C., Kawai T. (2012). Dyes Pigm..

[cit18] Chocron L., Baggi N., Ribeiro E., Goetz V., Yu P., Nakatani K., Métivier R. (2024). Chem. Sci..

[cit19] Peters A., Branda N. R. (2003). Chem. Commun..

[cit20] Guirado G., Coudret C., Hliwa M., Launay J. P. (2005). J. Phys. Chem. B.

[cit21] Browne W. R., De Jong J. J. D., Kudernac T., Walko M., Lucas L. N., Uchida K., Van Esch J. H., Feringa B. L. (2005). Chem.–Eur. J..

[cit22] Browne W. R., De Jong J. J. D., Kudernac T., Walko M., Lucas L. N., Uchida K., Van Esch J. H., Feringa B. L. (2005). Chem.–Eur. J..

[cit23] He B., Wenger O. S. (2011). J. Am. Chem. Soc..

[cit24] Gorodetsky B., Samachetty H. D., Donkers R. L., Workentin M. S., Branda N. R. (2004). Angew. Chem., Int. Ed..

[cit25] Léaustic A., Anxolabéhère-Mallart E., Maurel F., Midelton S., Guillot R., Métivier R., Nakatani K., Yu P. (2011). Chem.–Eur. J..

[cit26] Kleinwächter M., Teichmann E., Grubert L., Herder M., Hecht S. (2018). Beilstein J. Org. Chem..

[cit27] Sicard L., Lafolet F., Boggio-Pasqua M., Bucher C., Royal G., Cobo S. (2022). ChemPhysChem.

[cit28] Peters A., Branda N. R. (2003). J. Am. Chem. Soc..

[cit29] Moriyama Y., Matsuda K., Tanifuji N., Irie S., Irie M. (2005). Org. Lett..

[cit30] Gorodetsky B., Branda N. R. (2007). Adv. Funct. Mater..

[cit31] Gallardo I., Guirado G., Prats G., Takeshita M. (2009). Phys. Chem. Chem. Phys..

[cit32] Gallardo I., Guirado G., Moreno M., Prats G., Takeshita M. (2012). Chem.–Eur. J..

[cit33] Herder M., Utecht M., Manicke N., Grubert L., Pätzel M., Saalfrank P., Hecht S. (2013). Chem. Sci..

[cit34] Nakashima T., Atsumi K., Kawai S., Nakagawa T., Hasegawa Y., Kawai T. (2007). Eur. J. Org Chem..

[cit35] Fukumoto S., Nakashima T., Kawai T. (2011). Angew. Chem., Int. Ed..

[cit36] Li R., Nakashima T., Galangau O., Iijima S., Kanazawa R., Kawai T. (2015). Chem.–Asian J..

[cit37] Nakashima T., Kajiki Y., Fukumoto S., Taguchi M., Nagao S., Hirota S., Kawai T. (2012). J. Am. Chem. Soc..

[cit38] Calupitan J. P., Nakashima T., Hashimoto Y., Kawai T. (2016). Chem.–Eur. J..

[cit39] Baggi N., Léaustic A., Groni S., Anxolabéhère-Mallart E., Guillot R., Métivier R., Maurel F., Yu P. (2021). Chem.–Eur. J..

[cit40] Chatir E., Boggio-Pasqua M., Loiseau F., Philouze C., Royal G., Cobo S. (2022). Chem.–Eur. J..

[cit41] Audebert P., Miomandre F. (2013). Chem. Sci..

[cit42] Quinton C., Alain-Rizzo V., Dumas-Verdes C., Miomandre F., Clavier G., Audebert P. (2014). RSC Adv..

[cit43] Lin H.-T., Huang C.-L., Liou G.-S. (2019). ACS Appl. Mater. Interfaces.

[cit44] Sk B., Sarkar M., Singh K., Sengupta A., Patra A. (2021). Chem. Commun..

[cit45] Corrente G. A., González D. A., Aktas E., Capodilupo A. L., Ruighi F., Accorsi G., Imbardelli D., Rodriguez-Seco C., Martinez-Ferrero E., Palomares E., Beneduci A. (2023). J. Mater. Chem. C.

[cit46] Areephong J., Browne W. R., Katsonis N., Feringa B. L. (2006). Chem. Commun..

[cit47] Logtenberg H., Browne W. R. (2013). Org. Biomol. Chem..

[cit48] Connelly N. G., Geiger W. E. (1996). Chem. Rev..

[cit49] Johnson K. A., Simpson Z. B., Blom T. (2009). Anal. Biochem..

[cit50] Johnson K. A., Simpson Z. B., Blom T. (2009). Anal. Biochem..

[cit51] Woodward R. B., Hoffmann R. (1969). Angew. Chem., Int. Ed..

[cit52] Nakamura S., Irie M. (1988). J. Org. Chem..

[cit53] Perrier A., Maurel F., Aubard J. (2007). J. Photochem. Photobiol., A.

[cit54] Laurent A. D., André J.-M., Perpète E. A., Jacquemin D. (2007). J. Photochem. Photobiol., A.

