# Supplementary material for: Three-state molecular switch based on terarylene photo- or redox-induced reversible isomerisation
Source: Chem Sci. 2025 Jun 10;16(27):12499–509. doi: 10.1039/d5sc02845k (PMC12151078; doi:10.1039/d5sc02845k)
Supplement: SC-016-D5SC02845K-s001 [file SC-016-D5SC02845K-s001.pdf]

Supplementary Information (SI) for Chemical Science.
This journal is © The Royal Society of Chemistry 2025

Table of contents

General and methods - 2 -

Synthetic procedures - 5 -

Additional spectroscopic data - 15 -

Additional electrochemical data - 19 -

Additional spectroelectrochemical data - 23 -

Stopped-flow measurements and chemical red/ox experiments - 28 -

Coupled electrolysis-EPR spectroscopy - 32 -

Cyclic voltammetry simulations - 33 -

Time traces simulations - 40 -

Additional calculation data - 45 -

^{1}H- and ^{13}C-NMR spectra - 58 -

References - 69 -

General and methods:

Air-sensitive syntheses were performed under argon using Schlenk techniques. Anhydrous solvents were dried using standard methods, when necessary. Chemicals and solvents were used as received unless otherwise stated. Thin layer chromatography (TLC) was performed on silica gel 60 F_{254} while column chromatography was carried out on silica gel 60 (0.063-0.2 mm).

UV-vis spectroscopy: UV-vis spectra were recorded on a Varian Cary 5000 spectrometer. Cyclization and cycloreversion of the investigated photochromes were induced by light irradiation with a lamp (Oriel Hg(Xe) 200 W) equipped with narrow-band interference filters of appropriate wavelengths.

Fluorescence spectroscopy: Fluorescence spectra were recorded on a Fluoromax 4 spectrofluorometer from Jobin-Yvon/Horiba Co. Fluorescence quantum yields were determined by using 9,10-diphenylanthracene in cyclohexane as standard reference (Φ_F = 0.90).

Electrochemical measurements: Cyclic voltammetry measurements were carried out with an EGG PAR (model 273A) electrochemical workstation or a PGSTAT302N potentiostat (Metrohm Autolab). Measurements were performed at 20°C with a glassy carbon electrode (3 mm diameter) as working electrode, a saturated KCl calomel electrode as reference and a platinum counter electrode. Dry acetonitrile and/or dichloromethane were used to prepare the solutions of the investigated photochromes (10 mL, 1 mM). Tetrabutylammonium hexafluorophosphate ($TBAPF_6$, 0.1M) was used as electrolyte. The solutions were introduced in an argon-purged heart-shaped cell and protected with aluminum foil. Then, they were irradiated with a 365 nm LED to photochemically generate the closed form isomers.

Electrofluorochromism measurements: The electrofluochromism experiment was performed by using a three-electrode setup in a thin cell (optical path length = 1 mm), a PGSTAT204 potentiostat (Metrohm Autolab) and an Avantes AvaSpec-ULS4096CL-RS-EVO UV-Vis spectrophotometer. The working electrode was a 3 cm × 0.7 cm × 0.3 mm Pt grid with a Teflon-covered wire to avoid electrolysis elsewhere than in the quartz cell. A saturated KCl calomel electrode was used as reference and a platinum grid as counter. They were separated from the solution with fritted bridges. The cell was filled under argon with a dry dichloromethane solution of **20**. $TBAPF_6$ (0.1 M) was used as electrolyte.

Spectroelectrochemical measurements: The spectroelectrochemical analyses were performed by using a three-electrode setup in a thin cell (optical path length = 1 mm) placed in a UV-vis Varian Cary 60 spectrophotometer and a PGSTAT204 potentiostat (Metrohm Autolab). The working electrode was a 3 cm × 0.7 cm × 0.3 mm Pt grid with a Teflon-covered wire to avoid electrolysis elsewhere than in the quartz cell. A saturated KCl calomel electrode was used as reference and a platinum grid as counter. They were separated from the solution with fritted bridges. The cell was filled under argon with a dry acetonitrile solution of the photochromes. $TBAPF_6$ (0.1 M) was used as electrolyte.

Stopped flow absorption spectrophotometry: The measurements were performed on a BioLogic SFM-4000 coupled to a J&M Tidas diode array spectrometer (optical path: 1 cm), with a three-syringe setup (one containing **1c**$^{2+}$ 4.54×10^{-5} M in acetonitrile, the other containing the reductant in acetonitrile, the third used for solvent to record the blank). **1c**$^{2+}$ 4.54×10^{-5} M in acetonitrile was stored in syringe #4, decamethylferrocene 4.54×10^{-5} M or 8×10^{-5} M in acetonitrile was stored in syringe #1.

At t = 0 s, 150 µL of syringe #1 and 150 µL of syringe #4 were mixed, and spectra were recorded 10 ms after the mixing at 20°C.

Experiments were duplicated or triplicated for kinetic fits. Fits at 763 nm were performed using Kaleidagraph with a kinetic law corresponding to an exponential decay:

$$y = m1 + m2 \times \exp(-m3 \times t).$$

1c^{2+}was prepared in a setup consisting of a 6 mL cell containing **1o** (1.3 mg) and 6 mL of acetonitrile and NBu_4PF_6 0.1 M, and two compartments bearing a sintered glass frit at the end, filled with NBu_4PF_6 solution (0.1 M in acetonitrile) and in contact with the main solution. The working electrode (carbon foam) was placed in the main cell, the counter electrode (platinum grid) and reference electrode (SCE) were each placed in one of the compartments. The solution was electrolyzed at 1.2 V vs SCE for 2 hours (Figure S28). For the CV (Figure S28a), the Pt grid (CE) was replaced by a Pt wire and the working electrode was a glassy carbon electrode (3 mm diameter).

Coupled electrolysis-EPR spectroscopy: X-band EPR spectra were recorded on a Bruker ELEXSYS 500 spectrometer equipped with a Bruker ER 4116DM X band resonator and an Oxford Instruments continuous flow ESR 900 cryostat and ITC 503 temperature control system. The conditions used were: microwave frequency = 9.67 GHz; microwave power = 4.0 mW; modulation amplitude = 8 Gauss; modulation frequency = 100 KHz; gain = 50 dB; temperature = 298 K).

In situ EPR-cyclic voltammetry and electrolysis experiments were done using an *in-house* three-electrode setup consisting of two Pt spirals as working and counter electrodes and an Ag wire as pseudo-reference electrode that were placed inside an EPR tube.

To perform the experiments, the EPR tube housing the solution and the three electrodes were inserted in the cavity, the cavity was tuned, and the CV was obtained under EPR operating conditions.

For the electrolysis experiment, the desired potential was applied under operating conditions as the magnetic field was swept to obtain the spectra.

The electrochemical experiments were carried out with a PGSTAT204 potentiostat (Metrohm Autolab). Electrochemical data were collected with NOVA 2.1.

Cyclic voltammetry simulations: CV simulations were carried out using the DigiElch 8 software package (Elchsoft, Germany). Details are provided in the corresponding section (page 36).
Time traces simulations: The simulations of the time traces obtained with the stopped-flow experiments were performed using KinTek Explorer program.[1,2]

Computational details: The ground state structures of open (OF) and closed (CF) forms for terarylenes **1** – **5** in their different redox states were fully optimized at the DFT level of theory using ωB97X-D exchange-correlation functional (XCF) with the 6-311G(d,p) basis set. Next, frequencies were computed on the resulting optimized structures to ensure the absence of vibrational modes with imaginary wavenumber values. Molecular excitation energies of the different forms of **1** – **5** in different redox states were calculated using TDDFT (time-dependent density functional theory) with the optimized molecular structures at the ωB97X-D/6-311G(d,p) level. To understand the photochromic and redox-active properties and to support their interpretation from the experimental results, the computed absorption spectra need to be accurate enough. The dependence of excitation energies and electronic character of low-lying transitions was explored for different XCFs and basis sets for the compound **1**. We assessed a representative set of exchange-correlation functionals, namely, M06,[3] PBE0,[4] M062X,[3] MN15,[5] CAM-B3LYP,[6] MPW1PW91[7] and ωB97X-D,[8] which were all combined with the 6-311G(d,p) basis set. From this benchmark study, we selected the MPW1PW91 functional as it gave satisfactory results for the CF isomer in neutral state as well as in dicationic state (see Fig. 10 in the article). Both DFT and TD-DFT calculations were conducted by including solvent effects using the integral equation formalism of the polarizable continuum model (IEF-PCM),[9] considering acetonitrile as solvent during all calculations. All calculations were carried out with the Gaussian 16 code,[10] using default algorithms and parameters.

Synthetic procedures:

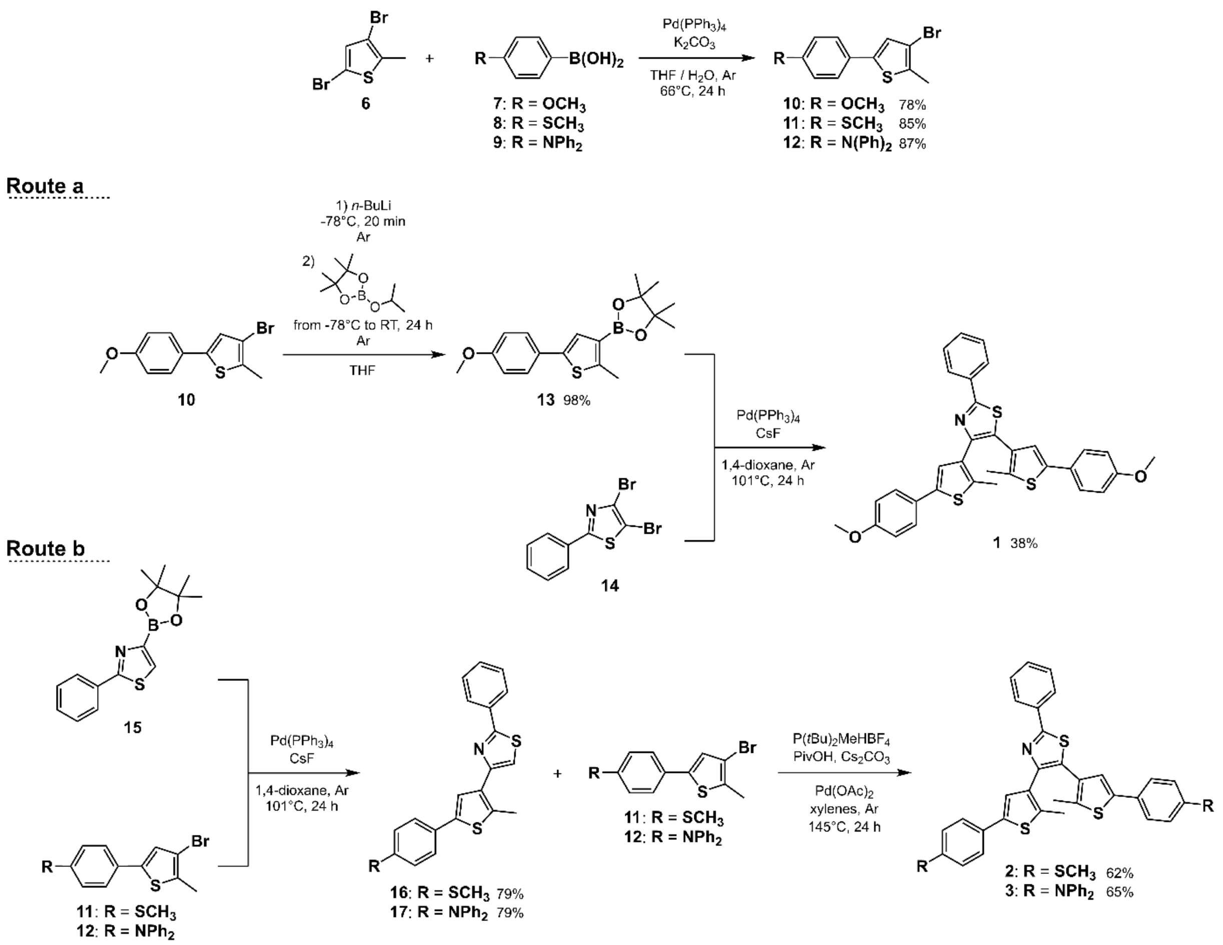

Scheme S1: Full synthetic routes a and b to afford photoswitches **1**, **2** and **3**.

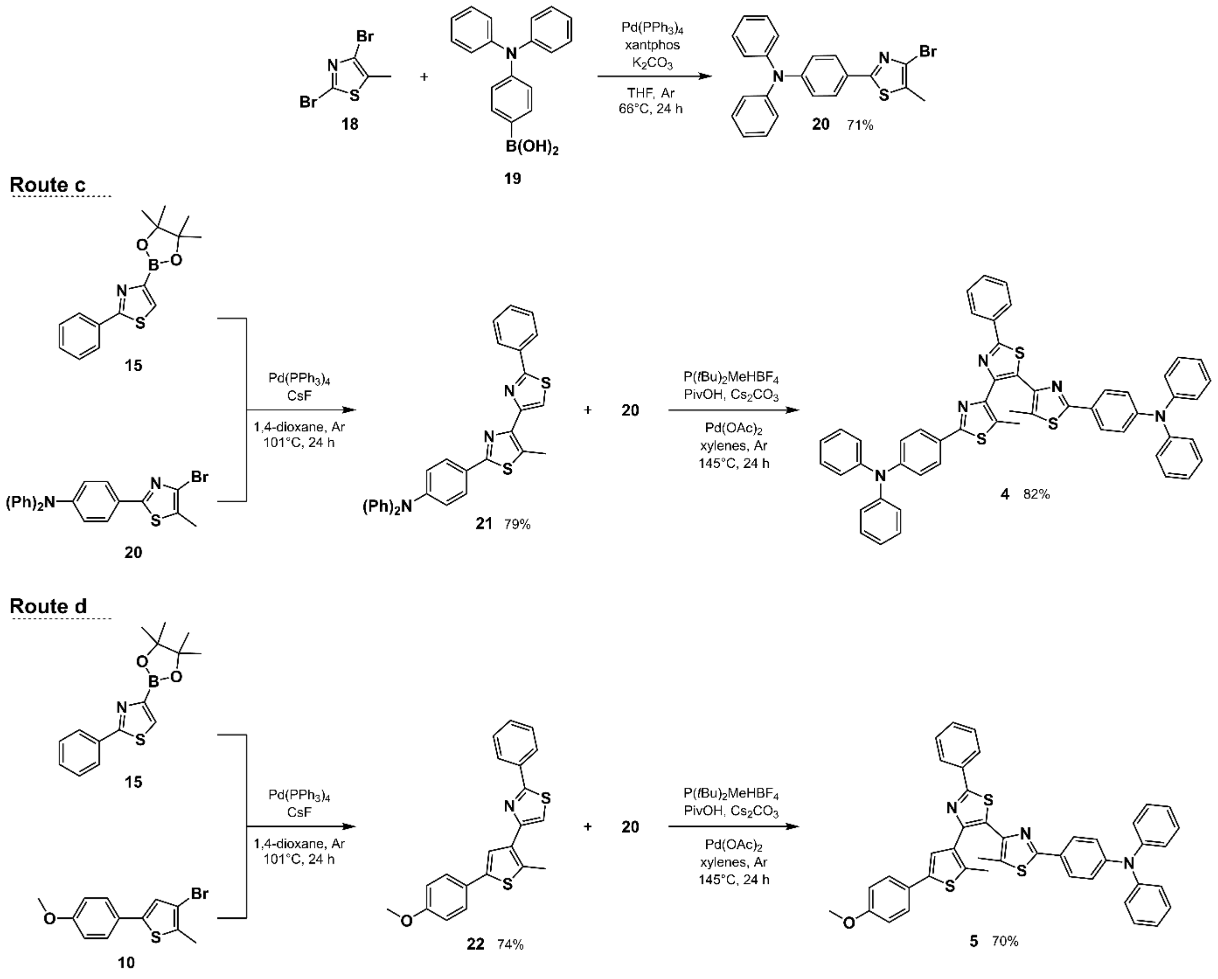

Scheme S2: Full synthetic routes c and d to afford photoswitches **4** and **5**.

3-bromo-5-(4-methoxyphenyl)-2-methylthiophene, **10**[11]

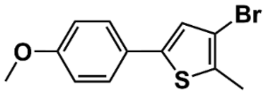

10

3,5-dibromo-2-methylthiophene, **6** (1.41 g, 0.705 mL, 5.5 mmol), 4-methoxyphenylboronic acid, **7** (920 mg, 6.05 mmol), $Pd(PPh_3)_4$ (254 mg, 0.22 mmol) and K_2CO_3 (835 mg, 6.05 mmol) were dissolved in a water / THF 1:1 mixture (40 mL). The biphasic mixture was stirred at 66°C under argon for 24 h, before being quenched with ethyl acetate (30 mL). The organic layer was extracted with ethyl acetate. The combined extracts were washed with brine, dried over anhydrous Na_2SO_4, and filtered. Evaporation of the solvent under vacuum afforded the crude product as yellow-brownish solid. Silica gel column chromatography (eluent: petroleum ether / dichloromethane 8:2) of the residue afforded pure **10** (1.22 g, 4.31 mmol, 78%) as a white crystalline solid. Data consistent with the literature.[11]

^{1}H-NMR ($CDCl_3$, 360 MHz): δ (ppm) 7.43 (d, *J* = 8.9 Hz, 1H), 6.98 (s, 1H), 6.90 (d, *J* = 8.9 Hz, 1H), 3.83 (s, 3H), 2.40 (s, 3H).
^{13}C-NMR ($CDCl_3$, 75 MHz): δ (ppm) 159.5, 141.2, 132.6, 126.7, 126.5, 124.5, 114.4, 109.7, 55.4, 14.9.

3-bromo-2-methyl-5-(4-(methylthio)phenyl)thiophene, **11**[12]

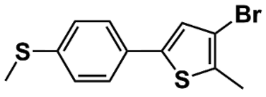

11

The previous procedure was followed for the reaction between **6** and 4-(methylthio)phenylboronic acid, **8** (505 mg, 3 mmol) to afford pure **11** (673 mg, 2.25 mmol, 75%) as a white solid after purification by silica gel column chromatography (eluent: petroleum ether / dichloromethane 9:1). Data consistent with the literature.[12]

^{1}H-NMR ($CDCl_3$, 300 MHz): δ (ppm) 7.42 (d, *J* = 8.5 Hz, 2H), 7.24 (d, *J* = 8.5 Hz, 2H), 7.07 (s, 1H), 2.50 (s, 3H), 2.41 (s, 3H).
^{13}C-NMR ($CDCl_3$, 75 MHz): δ (ppm) 140.8, 138.4, 133.4, 130.4, 126.9, 125.7, 125.3, 110.0, 15.8, 14.9.

4-(4-bromo-5-methylthiophen-2-yl)-N,N-diphenylaniline, **12**[13]

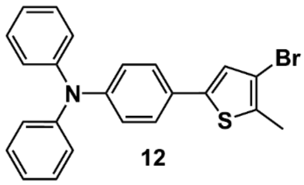

12

The previous procedure was followed for the reaction between **6** and 4-(N,N-diphenylamino)phenylboronic acid, **9** (867 mg, 3 mmol) to afford pure **12** (1.10 g, 2.61 mmol, 87%) as a light-yellow solid after purification by re-precipitation from CH_3OH. Data consistent with the literature.[13]

^{1}H-NMR ($CDCl_3$, 300 MHz): δ (ppm) 7.36 (d, *J* = 8.7 Hz, 2H), 7.30 – 7.24 (m, 4H), 7.11 (m, 4H), 7.04 (m, 4H), 7.00 (s, 1H), 2.40 (s, 3H).
^{13}C-NMR ($CDCl_3$, 75 MHz): δ (ppm) 147.6, 147.5, 141.2, 132.8, 129.5, 127.5, 126.2, 124.7, 124.7, 123.6, 123.3, 109.9, 14.9.
HRMS (ESI): calcd. for $C_{23}H_{19}BrNS^+$ $[M+H]^+$ 420.0416, found $[M+H]^+$ 420.0401.

2-(5-(4-methoxyphenyl)-2-methylthiophen-3-yl)-4,4,5,5-tetramethyl-1,3,2-dioxaborolane, **13**[11]

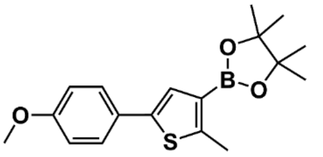

13

3-bromo-5-(4-methoxyphenyl)-2-methylthiophene, **10** (1.21 g, 4.28 mmol) was dissolved in distilled dry THF (20 mL) and the resulting solution was cooled to 78°C under Ar. Then, *n*-BuLi (2.5 M, 2.20 mL, 5.35 mmol) was added dropwise and the system was left stirring at -78°C under Ar. After 40 min, 2-isopropoxy-4,4,5,5-tetramethyl-1,3,2-dioxaborolane (995 mg, 1.10 mL, 5.35 mmol) was slowly added to the solution and the resulting system was stirred for 24 h while letting the temperature rise to room temperature under argon. After quenching the reaction mixture with NH_4Cl 1M solution (30 mL), the organic layer was extracted with ethyl acetate. The combined extracts were washed with brine, dried over anhydrous Na_2SO_4, and filtered. Removal of the solvent under reduced pressure afforded **13** (1.39 g, 4.20 mmol, 98%) as an oil that later crystallized as a white solid. It was used without further purification in the next reactions. Data consistent with the literature.[11]

^{1}H-NMR ($CDCl_3$, 300 MHz): δ (ppm) 7.49 (d, *J* = 8.9 Hz, 2H), 7.30 (s, 1H), 6.87 (d, *J* = 8.9 Hz, 2H), 3.82 (s, 3H), 2.68 (s, 3H), 1.33 (s, 12H).
^{13}C-NMR ($CDCl_3$, 75 MHz): δ (ppm) 158.9, 151.6, 140.9, 127.9, 127.6, 127.1, 114.3, 83.4, 55.5, 25.0, 16.0.

4-(2-methyl-5-(4-(methylthio)phenyl)thiophen-3-yl)-2-phenylthiazole, **16**

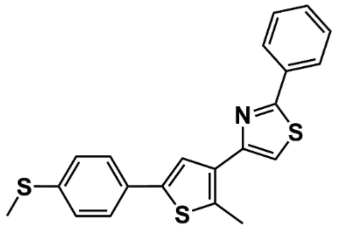

16

3-bromo-2-methyl-5-(4-(methylthio)phenyl)thiophene, **11** (150 mg, 0.50 mmol), 2-phenyl-4-(4,4,5,5-tetramethyl-1,3,2-dioxaborolan-2-yl)thiazole, **15**[14] (160 mg, 0.56 mmol), CsF (199 mg, 1.31 mmol) and $Pd(PPh_3)_4$ (23 mg, 0.02 mmol) were partially solubilized in dry 1,4-dioxane (10 mL) and the obtained mixture was stirred at reflux under argon for 24 h. The reaction was quenched with water (15 mL) and extracted with chloroform. The combined extracts were washed with brine, dried over anhydrous Na_2SO_4, and filtered. The filtrate was concentrated under reduced pressure giving the crude product as an oil. Purification by silica gel column chromatography (eluent: from petroleum ether / dichloromethane 7:3 to petroleum ether / dichloromethane 6:4) afforded pure **16** (150 mg, 0.40 mmol, 79%) as a white solid.

^{1}H-NMR ($CDCl_3$, 300 MHz): δ (ppm) 8.04 (m, 2H), 7.61 (s, 1H), 7.54 (d, *J* = 8.4 Hz, 2H), 7.47 (m, 4H), 7.28 (s, 2H), 2.76 (s, 3H), 2.51 (s, 3H).
^{13}C-NMR ($CDCl_3$, 75 MHz): δ (ppm) 167.3, 152.5, 139.5, 137.5, 136.0, 133.8, 132.9, 131.4, 130.2, 129.1, 127.1, 126.7, 126.0, 124.2, 113.9, 16.0, 15.5.
HRMS (ESI): calcd. for $C_{21}H_{18}NS_3^+$ $[M+H]^+$ 380.0596, found $[M+H]^+$ 380.0589.

4-(5-methyl-4-(2-phenylthiazol-4-yl)thiophen-2-yl)-N,N-diphenylaniline, **17**

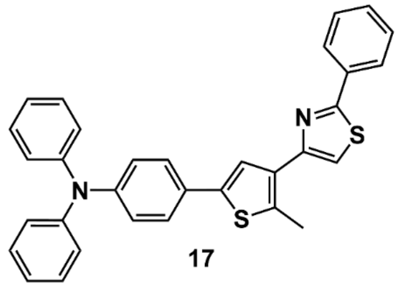

17

The previous procedure was followed for the reaction between **15** and 4-(4-bromo-5-methylthiophen-2-yl)-N,N-diphenylaniline, **12** (210 mg, 0.50 mmol) to afford **17** (197 mg, 0.40 mmol, 79%) as a cream white solid after purification by re-precipitation from CH_3OH.

^{1}H-NMR ($CDCl_3$, 360 MHz): δ (ppm) 8.04 (dd, *J* = 7.6, 2.0 Hz, 2H), 7.54 (s, 1H), 7.49 – 7.45 (m, 5H), 7.29 – 7.25 (m, 4H), 7.14 – 7.01 (m, 9H), 2.76 (s, 3H).
^{13}C-NMR ($CDCl_3$, 75 MHz): δ (ppm) 167.2, 152.6, 147.7, 147.1, 139.8, 135.5, 133.8, 132.8, 130.1, 129.4, 129.0, 128.6, 126.7, 126.5, 124.5, 123.9, 123.6, 123.1, 113.8, 15.5.
HRMS (ESI): calcd. for $C_{32}H_{25}N_2S_2^+$ $[M+H]^+$ 501.1454, found $[M+H]^+$ 501.1435; calcd. for $C_{32}H_{24}N_2NaS_2^+$ $[M+Na]^+$ 523.1273, found $[M+Na]^+$ 523.1254.

4-(4-bromo-5-methylthiazol-2-yl)-N,N-diphenylaniline, **20**

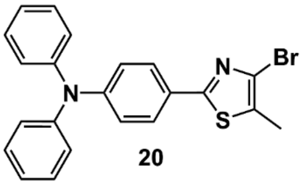

20

4-(N,N-diphenylamino)phenylboronic acid, **19** (292 mg, 1.01 mmol), 2,4-dibromo-5-methylthiazole, **18** (259 mg, 1.01 mmol), xantphos (15 mg, 0.03 mmol), $Pd(OAc)_2$ (7 mg, 0.03 mmol) and K_3PO_4 (658 mg, 3.1 mmol) were partially dissolved in distilled dry THF (10 mL). The obtained mixture was stirred at reflux under argon for 24 h. Once cooled down to room temperature, it was diluted with chloroform (10 mL) and filtered on a Celite pad. Multiple washings with chloroform were carried out and the obtained filtrate was concentrated under vacuum. The obtained crude product was purified by silica gel column chromatography (eluent: petroleum ether / dichloromethane 6:4) to afford 4-(4-bromo-5-methylthiazol-2-yl)-N,N-diphenylaniline, **20** (300 mg, 0.72 mmol, 71%) as a pale yellow crystalline solid.

^{1}H-NMR ($CDCl_3$, 300 MHz): δ (ppm) 7.70 (d, *J* = 8.8 Hz, 2H), 7.32 – 7.26 (m, 4H), 7.16 – 7.06 (m, 6H), 7.04 (d, *J* = 8.8 Hz, 2H), 2.41 (s, 3H).
^{13}C-NMR ($CDCl_3$, 75 MHz): δ (ppm) 165.4, 149.8, 147.1, 129.6, 127.4, 127.0, 126.3, 125.3, 125.0, 123.9, 122.1, 13.1.
HRMS (ESI): calcd. for $C_{22}H_{18}BrN_2S^+$ $[M+H]^+$ 421.0368, found $[M+H]^+$ 421.0360; calcd. for $C_{22}H_{17}BrN_2NaS^+$ $[M+Na]^+$ 443.0188, found $[M+Na]^+$ 443.0178.

4-(5-methyl-2'-phenyl-[4,4'-bithiazol]-2-yl)-N,N-diphenylaniline, **21**

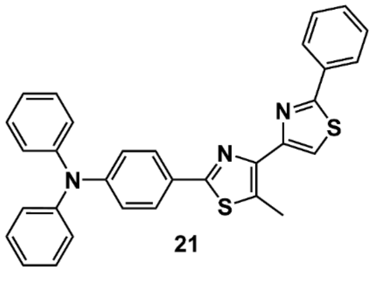

21

4-(4-bromo-5-methylthiazol-2-yl)-N,N-diphenylaniline, **20** (176 mg, 0.42 mmol), 2-phenyl-4-(4,4,5,5-tetramethyl-1,3,2-dioxaborolan-2-yl)thiazole, **15** (138 mg, 0.48 mmol), CsF (170 mg, 1.12 mmol) and $Pd(PPh_3)_4$ (27 mg, 0.02 mmol) were partially solubilized in dry 1,4-dioxane (10 mL) and the obtained light yellow mixture was stirred at reflux under argon for 24 h. The reaction was quenched with water (20 mL) and extracted with chloroform. The combined extracts were washed with brine, dried over anhydrous Na_2SO_4, and filtered. The filtrate was concentrated under vacuum giving the crude product as a greenish solid which was purified by silica gel column chromatography (eluent: dichloromethane / petroleum ether 1:1) to afford pure 4-(5-methyl-2'-phenyl-[4,4'-bithiazol]-2-yl)-N,N-diphenylaniline, **77** (166 mg, 0.33 mmol, 79%) as a pale green solid.

^{1}H-NMR ($CDCl_3$, 250 MHz): δ (ppm) 8.04 (m, 2H), 7.88 (s, 1H), 7.81 (d, *J* = 8.7 Hz, 2H), 7.46 (m, 3H), 7.32 – 7.25 (m, 4H), 7.16 – 7.04 (m, 8H), 2.97 (s, 3H).

^{13}C-NMR ($CDCl_3$, 75 MHz): δ (ppm) 167.5, 163.8, 152.8, 149.4, 147.3, 145.5, 134.0, 130.4, 130.0, 129.5, 129.0, 127.5, 127.4, 126.6, 125.1, 123.7, 122.7, 116.5, 13.2.
HRMS (ESI): calcd. for $C_{31}H_{24}N_3S_2^+$ $[M+H]^+$ 502.1406, found $[M+H]^+$ 502.1400; calcd. for $C_{31}H_{23}N_3NaS_2^+$ $[M+Na]^+$ 524.1226, found $[M+Na]^+$ 524.1219.

4-(5-(4-methoxyphenyl)-2-methylthiophen-3-yl)-2-phenylthiazole, **22**

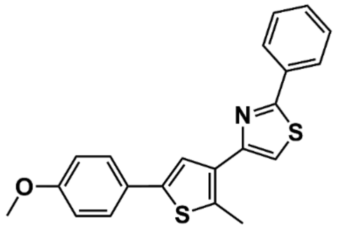

22

The previous procedure was repeated by replacing **20** with 3-bromo-5-(4-methoxyphenyl)-2-methylthiophene, **10** (566 mg, 2 mmol) to afford 4-(5-(4-methoxyphenyl)-2-methylthiophen-3-yl)-2-phenylthiazole, **22** (538 mg, 1.48 mmol, 74%) as white crystalline solid after purification by silica gel column chromatography (eluent: initially petroleum ether / dichloromethane 7:3, then petroleum ether / dichloromethane 6:4).

^{1}H-NMR ($CDCl_3$, 300 MHz): δ (ppm) 8.05 – 8.02 (m, 2H), 7.54 (d, *J* = 8.8 Hz, 2H), 7.51 (s, 1H), 7.49 – 7.43 (m, 3H), 7.27 (s, 1H), 6.92 (d, *J* = 8.8 Hz, 2H), 3.84 (s, 3H), 2.75 (s, 3H).
^{13}C-NMR ($CDCl_3$, 75 MHz): δ (ppm) 167.2, 159.1, 152.6, 139.9, 135.2, 133.8, 132.7, 130.1, 129.0, 127.3, 126.9, 126.7, 123.4, 114.4, 113.8, 55.5, 15.4.
HRMS (ESI): calcd. for $C_{21}H_{18}NOS_2^+$ $[M+H]^+$ 364.0824, found $[M+H]^+$ 364.0820.

Terarylene **1**

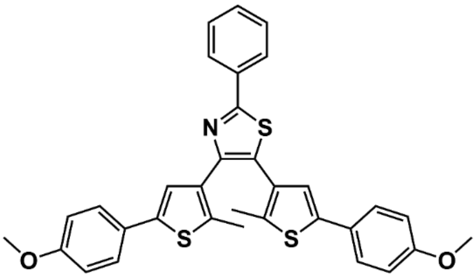

1

Route a: 2-(5-(4-methoxyphenyl)-2-methylthiophen-3-yl)-4,4,5,5-tetramethyl-1,3,2-dioxaborolane, **13** (380 mg, 1.15 mmol), 2-phenyl-4,5-dibromothiazole, **14**[15,16] (160 mg, 0.50 mmol), CsF (380 mg, 2.50 mmol) and $Pd(PPh_3)_4$ (52 mg, 0.045 mmol) were partially solubilized in dry 1,4-dioxane (15 mL) and the obtained mixture was stirred at reflux under argon for 24 h. Then, the reaction was quenched with water (20 mL) and extracted with chloroform. The combined organic phase was washed with brine, dried over anhydrous Na_2SO_4 and filtered. The solvents were removed under vacuum and the obtained residue was purified by silica gel column chromatography (eluent: initially dichloromethane / petroleum ether 6:4, then

dichloromethane / petroleum ether 8:2) to afford **1** (108 mg, 0.19 mmol, 38%) as a light blue solid.

^{1}H-NMR ($CDCl_3$, 300 MHz): δ (ppm) 8.07 – 8.00 (m, 2H), 7.50 – 7.38 (m, 7H), 7.11 (s, 1H), 7.02 (s, 1H), 6.93 – 6.83 (m, 4H), 3.83 (s, 3H), 3.81 (s, 3H), 2.33 (s, 3H), 2.20 (s, 3H).
^{13}C-NMR ($CDCl_3$, 91 MHz): δ (ppm) 165.7, 159.3, 159.0, 148.6, 140.7, 140.0, 136.8, 136.4, 133.8, 133.0, 130.1, 129.2, 129.0, 128.1, 127.4, 126.9, 126.8, 126.5, 124.2, 123.8, 114.4, 114.3, 55.4, 14.7, 14.3.
HRMS (ESI): calcd. for $C_{33}H_{28}NO_2S_3^+$ $[M+H]^+$ 566.1277, found $[M+H]^+$ 566.1258.

Terarylene **2**

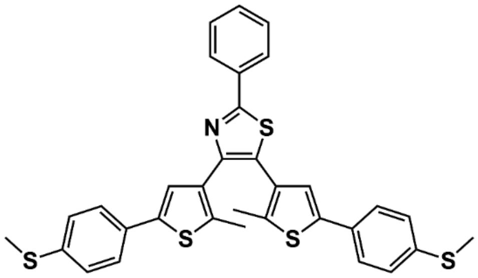

2

Route b: 4-(2-methyl-5-(4-(methylthio)phenyl)thiophen-3-yl)-2-phenylthiazole, **16** (132 mg, 0.35 mmol), 3-bromo-2-methyl-5-(4-(methylthio)phenyl)thiophene, **11** (105 mg, 0.35 mmol), $P(tBu)_2MeHBF_4$ (17 mg, 0.07 mmol), Cs_2CO_3 (237 mg, 0.73 mmol), pivalic acid (12 mg, 0.12 mmol) and $Pd(OAc)_2$ (6.2 mg, 0.03 mmol) were partially solubilized in dry xylenes (3 mL) and the obtained mixture was stirred at reflux for 24 hours. The reaction was then diluted with chloroform and filtered through a Celite pad. The obtained solution was washed with water, dried over anhydrous Na_2SO_4 and filtered. The filtrate was concentrated under vacuum and silica gel column chromatography (eluent: from dichloromethane / petroleum ether 4:6 to dichloromethane / petroleum ether 1:1) of the residue afforded pure **2** (130 mg, 0.22 mmol, 62%) as a cream white solid.

^{1}H-NMR ($CDCl_3$, 360 MHz): δ (ppm) 8.14 – 8.06 (m, 2H), 7.50 – 7.47 (m, 3H), 7.45 – 7.40 (m, 4H), 7.25 – 7.18 (m, 5H), 7.09 (s, 1H), 2.50 (s, 3H), 2.49 (s, 3H), 2.32 (s, 3H), 2.21 (s, 3H).
^{13}C-NMR ($CDCl_3$ + drops of methanol-d_4, 91 MHz): δ (ppm) 166.0, 148.3, 140.3, 139.6, 137.9, 137.5, 137.3, 137.1, 133.4, 132.9, 131.3, 130.7, 130.2, 129.1, 129.0, 128.0, 127.0, 126.9, 126.4, 125.8, 125.8, 124.8, 124.4, 15.9, 15.8, 14.5, 14.3.
HRMS (ESI): calcd. for $C_{33}H_{28}NS_5^+$ $[M+H]^+$ 598.0820, found $[M+H]^+$ 598.0778.

Terarylene **3**

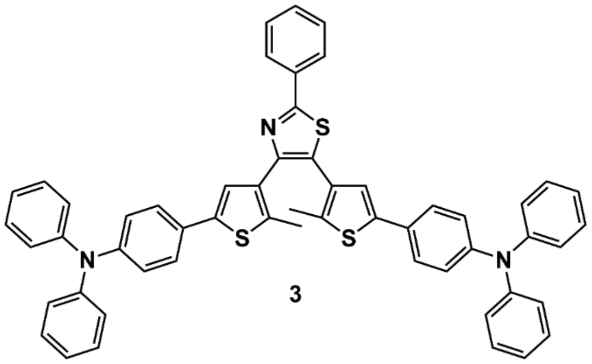

3

The previous route b was followed for the reaction between **17** (150 mg, 0.30 mmol) and **12** (126 mg, 0.30 mmol) to afford pure **3** (164 mg, 0.195 mmol, 65%) as a greenish solid after silica gel column chromatography (eluent: from dichloromethane / petroleum ether 3:7 to dichloromethane / petroleum ether 4:6) and washing with diethyl ether.

^{1}H-NMR ($CDCl_3$, 300 MHz): δ (ppm) 8.04 – 8.01 (m, 2H), 7.47 – 7.43 (m, 3H), 7.39 – 7.34 (m, 5H), 7.29 – 7.28 (m, 2H), 7.26 – 7.22 (m, 5H), 7.13 – 7.08 (m, 9H), 7.06 – 7.01 (m, 9H), 2.34 (s, 3H), 2.19 (s, 3H).
^{13}C-NMR ($CDCl_3$, 91 MHz): δ (ppm) 165.7, 148.5, 147.7, 147.6, 147.4, 147.0, 140.7, 140.0, 137.0, 136.6, 133.8, 133.1, 130.1, 129.4, 129.4, 129.3, 129.1, 128.8, 128.1, 128.1, 126.5, 126.4, 126.4, 124.6, 124.5, 124.4, 124.0, 124.0, 123.8, 123.2, 123.1, 14.8, 14.4.
HRMS (ESI): The sample could not be ionized to perform the analysis.

Terarylene **4**

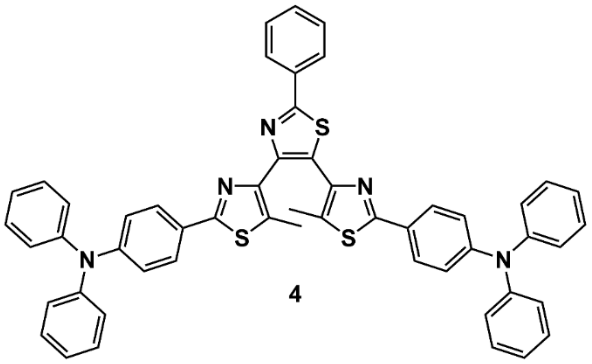

4

Route c: 4-(5-methyl-2'-phenyl-[4,4'-bithiazol]-2-yl)-N,N-diphenylaniline, **21** (136 mg, 0.271 mmol), 4-(4-bromo-5-methylthiazol-2-yl)-N,N-diphenylaniline, **20** (115 mg, 0.273 mmol), $P(tBu)_2MeHBF_4$ (7 mg, 0.03 mmol), Cs_2CO_3 (176 mg, 0.54 mmol), pivalic acid (8.80 mg, 0.09 mmol) and $Pd(OAc)_2$ (5 mg, 0.02 mmol) were partially solubilized in dry xylenes (3 mL) and the obtained mixture was stirred at reflux for 24 hours. The reaction was diluted with dichloromethane and filtered through a Celite pad. The obtained solution was washed with water, dried over anhydrous Na_2SO_4, and filtered. The filtrate was concentrated under vacuum and silica gel column chromatography (eluent: dichloromethane) of the residue afforded pure photochrome **4** (188 mg, 0.222 mmol, 82%) as a pale green solid thanks to a re-precipitation from a mixture of diethyl ether and ethanol 96%.

^{1}H-NMR ($CDCl_3$, 360 MHz): δ (ppm) 8.05 (dd, *J* = 7.4, 2.2 Hz, 2H), 7.75 (d, *J* = 8.7 Hz, 2H), 7.66 (d, *J* = 8.7 Hz, 2H), 7.45 (m, 3H), 7.33 – 7.24 (m, 8H), 7.19 – 6.94 (m, 16H), 2.46 (s, 3H), 2.08 (s, 3H).
^{13}C-NMR ($CDCl_3$, 91 MHz): δ (ppm) 167.1, 164.2, 163.9, 149.5, 149.2, 147.6, 147.3, 147.2, 146.3, 143.5, 133.7, 132.1, 131.3, 130.2, 129.8, 129.5, 129.0, 127.5, 127.4, 127.4, 127.1, 126.7, 125.1, 125.1, 123.7, 123.6, 122.5, 122.5, 12.7, 12.4.
HRMS (ESI): The sample could not be ionized to perform the analysis.

Terarylene **5**

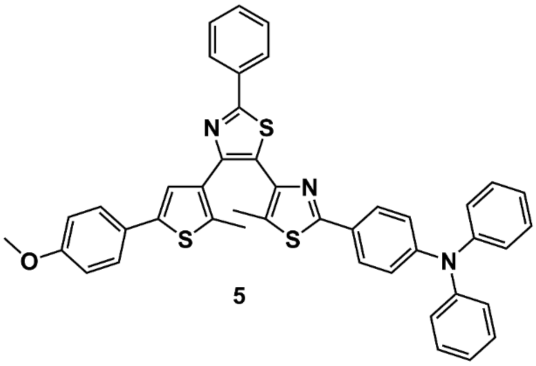

5

Route d: 4-(5-(4-methoxyphenyl)-2-methylthiophen-3-yl)-2-phenylthiazole, **22** (128 mg, 0.35 mmol), 4-(4-bromo-5-methylthiazol-2-yl)-N,N-diphenylaniline, **20** (149 mg, 0.35 mmol), $P(tBu)_2MeHBF_4$ (9 mg, 0.04 mmol), Cs_2CO_3 (230 mg, 0.70 mmol), pivalic acid (11 mg, 0.11 mmol) and $Pd(OAc)_2$ (6.40 mg, 0.03 mmol) were partially solubilized in dry xylenes (3 mL) and the obtained mixture was stirred at reflux for 24 hours. The reaction was diluted with chloroform (15 mL) and water (15 mL). The organic layer was extracted with chloroform. The obtained solution was washed with water, dried over anhydrous Na_2SO_4, and filtered. The filtrate was concentrated under vacuum and silica gel column chromatography (eluent: dichloromethane) of the residue afforded pure photochrome **5** (172 mg, 0.245 mmol, 70%) as a pale green solid thanks to a precipitation from a dichloromethane solution induced by methanol.

^{1}H-NMR ($CDCl_3$, 360 MHz): δ (ppm) 8.05 – 8.03 (m, 2H), 7.78 (d, *J* = 8.7 Hz, 2H), 7.47 – 7.42 (m, 5H), 7.32 – 7.27 (m, 4H), 7.16 – 7.13 (m, 5H), 7.11 – 7.06 (m, 4H), 6.87 (d, *J* = 8.8 Hz, 2H), 3.82 (s, 3H), 2.36 (s, 3H), 2.01 (s, 3H).
^{13}C-NMR ($CDCl_3$, 91 MHz): δ (ppm) 166.9, 164.7, 159.0, 149.6, 149.1, 147.2, 143.4, 140.2, 136.8, 133.7, 133.3, 131.1, 130.2, 129.6, 129.0, 127.8, 127.4, 127.3, 126.9, 126.8, 126.6, 125.2, 123.8, 122.5, 114.3, 55.5, 14.6, 12.3.
HRMS (ESI): calcd. for $C_{43}H_{34}N_3OS_3^+$ $[M+H]^+$ 704.1858, found $[M+H]^+$ 704.1836.

Additional spectroscopic data:

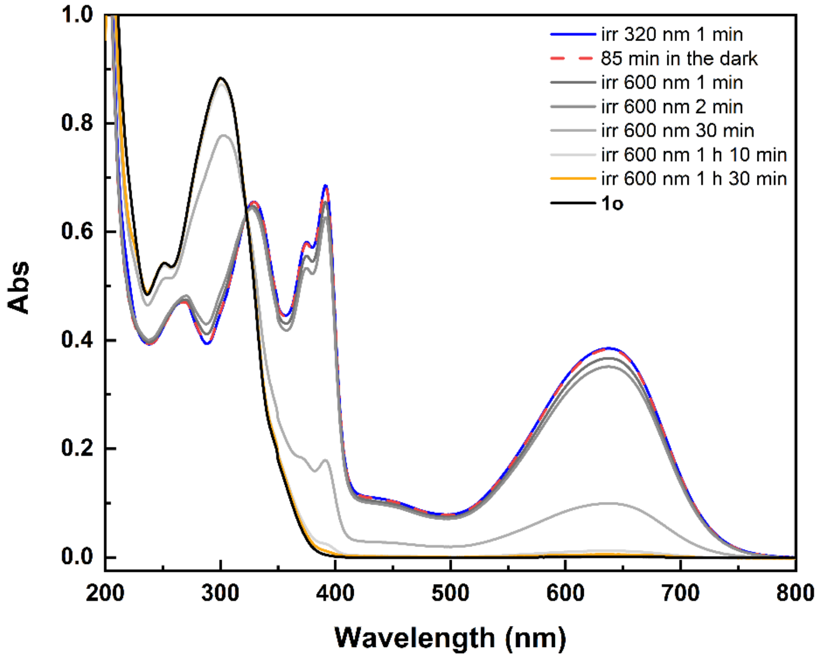

Figure S1: Spectral evolution of **1** (1.84 × 10^{-5} M) in CH_3CN under visible light irradiation (600 nm) from **1c**-rich photo-stationary state (blue solid line) to the open form (**1o**, black solid line). The spectrum recorded after having kept the UV-irradiated solution in the dark at room temperature for 85 min is provided with a red dashed line. Optical path of the cuvette: 1 cm.

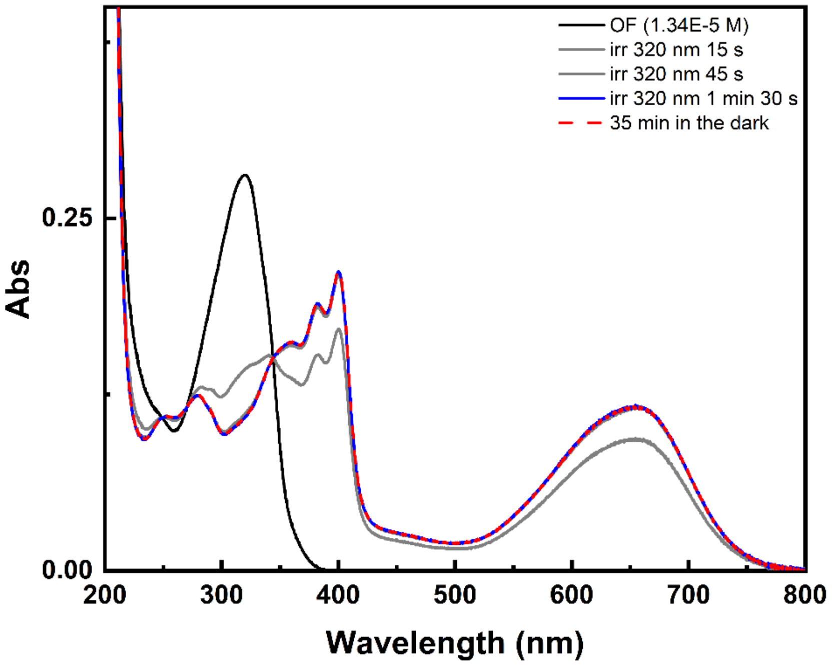

Figure S2: Spectral evolution of **2** in CH_3CN + 2% v/v CH_2Cl_2 under UV light irradiation (320 nm) from the black line (open form) to the blue line (photo-stationary state). The spectrum

recorded after having kept the irradiated solution in the dark at room temperature is provided as red dashed line. Optical path of the cuvette: 1 cm.

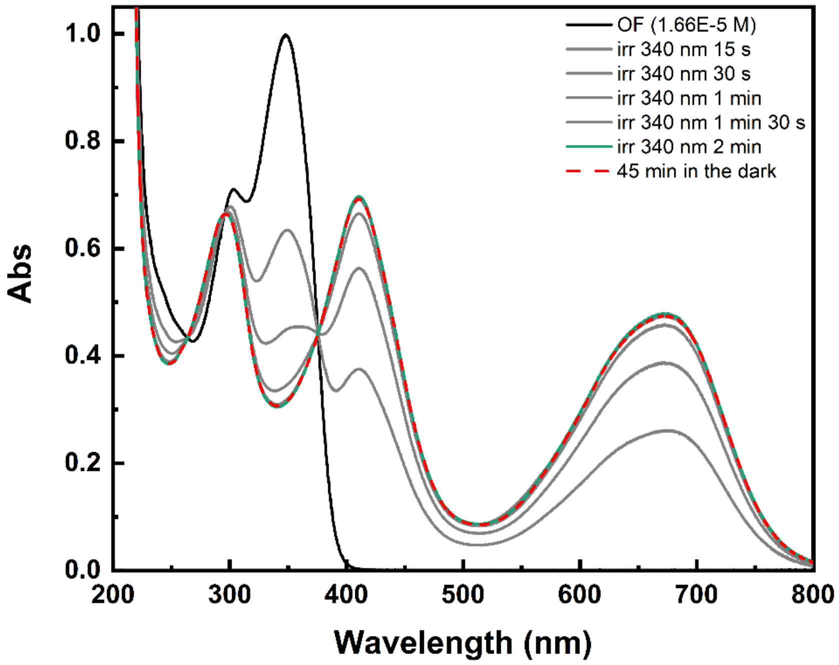

Figure S3: Spectral evolution of **3** in CH_3CN + 2% v/v CH_2Cl_2 under UV light irradiation (340 nm) from the black line (open form) to the green line (photo-stationary state). The spectrum recorded after having kept the irradiated solution in the dark at room temperature is provided as red dashed line. Optical path of the cuvette: 1 cm.

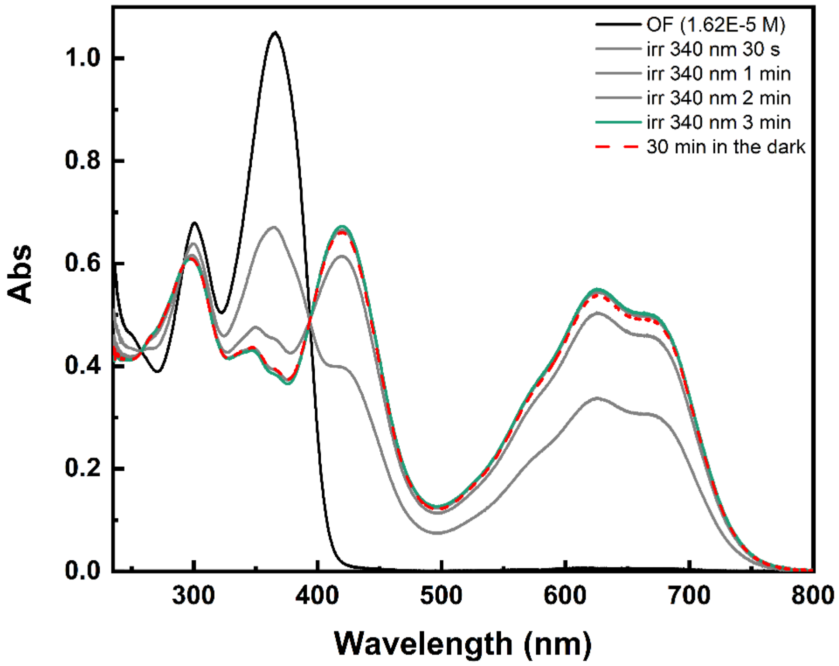

Figure S4: Spectral evolution of **4** in CH_2Cl_2 under UV light irradiation (340 nm) from the black line (open form) to the green line (photo-stationary state). The spectrum recorded after having

kept the irradiated solution in the dark at room temperature is provided as red dashed line. Optical path of the cuvette: 1 cm.

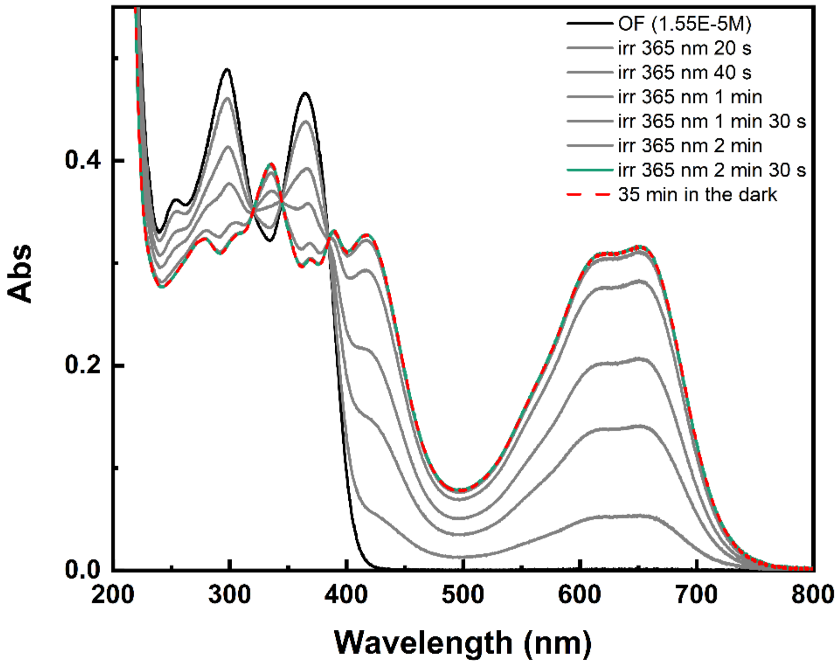

Figure S5: Spectral evolution of **5** in CH_3CN + 2% v/v CH_2Cl_2 under UV light irradiation (365 nm) from the black line (open form) to the green line (photo-stationary state). The spectrum recorded after having kept the irradiated solution in the dark at room temperature is provided as red dashed line. Optical path of the cuvette: 1 cm.

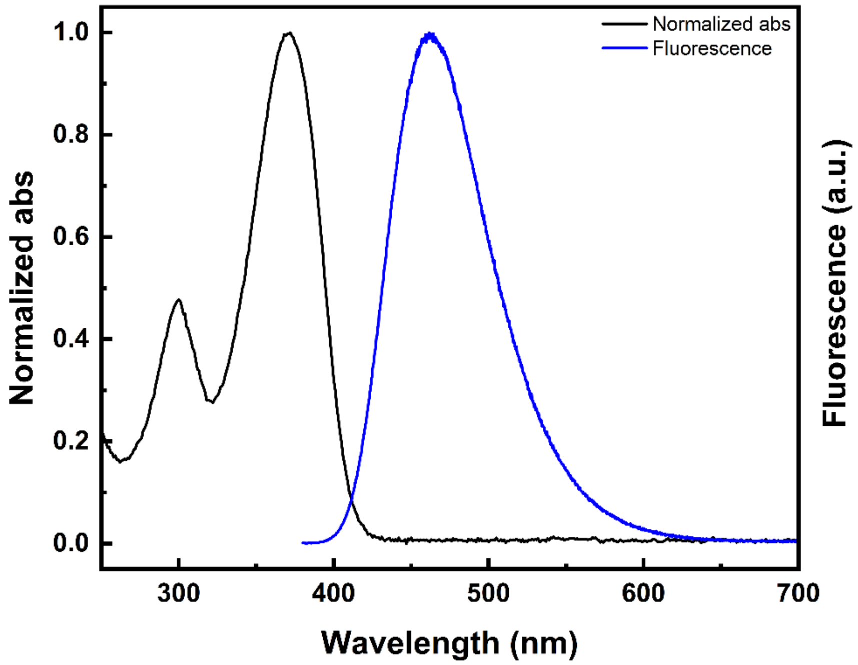

Figure S6: (black solid line) normalized UV-vis spectrum of **20** in dichloromethane; (blue solid line) emission spectrum of **20** in dichloromethane (λ_{exc} = 370 nm).

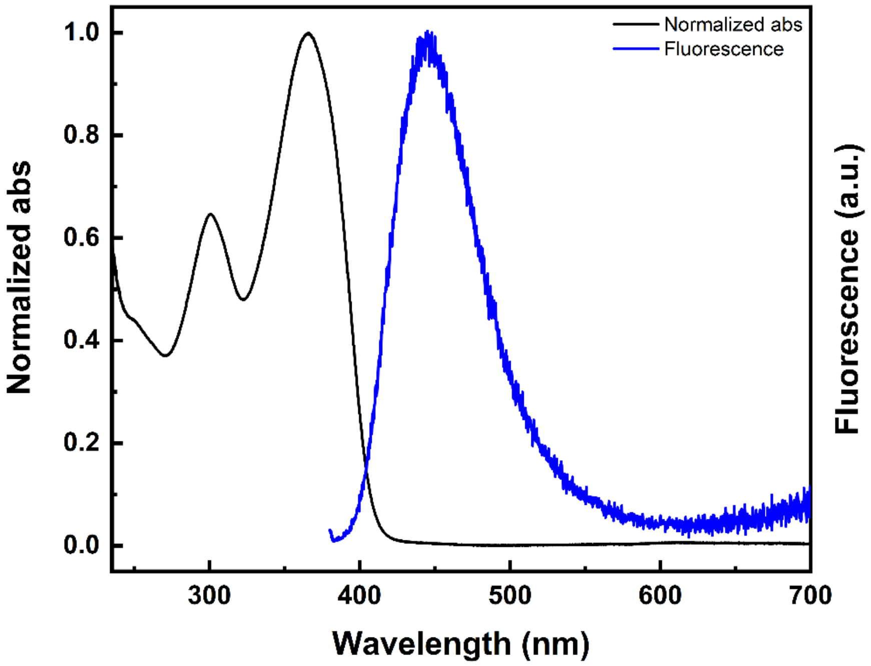

Figure S7: (black solid line) normalized UV-vis spectrum of **4** in dichloromethane; (blue solid line) emission spectrum of **4** in dichloromethane (λ_{exc} = 370 nm).

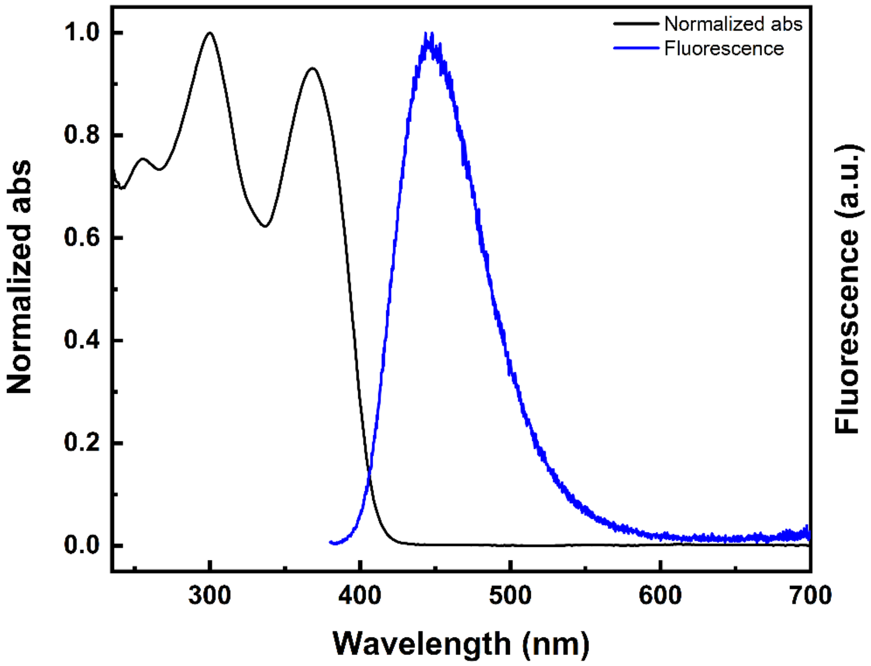

Figure S8: (black solid line) normalized UV-vis spectrum of **5** in dichloromethane; (blue solid line) emission spectrum of **5** in dichloromethane (λ_{exc} = 370 nm).

Additional electrochemical data:

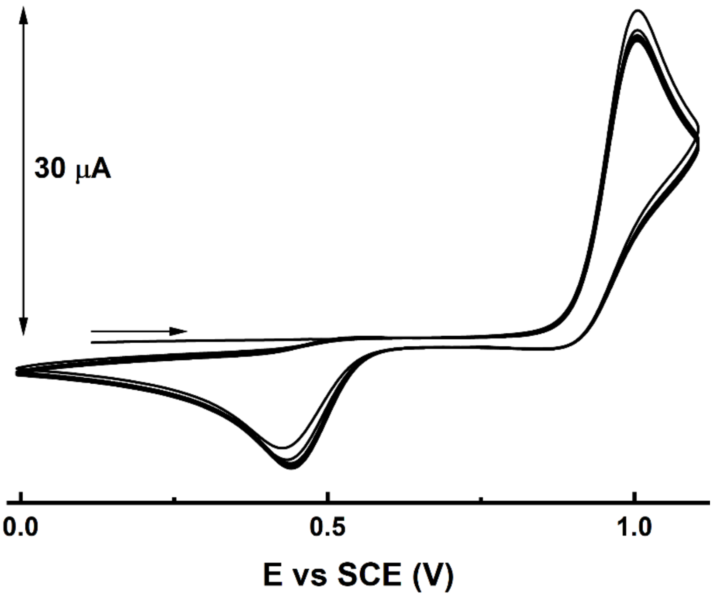

Figure S9: CVs of **4o** (1 mM) in CH_2Cl_2 / $TBAPF_6$ 0.1 M. 25 oxidation-reduction cycles at ν = 100 mV s^{-1}.

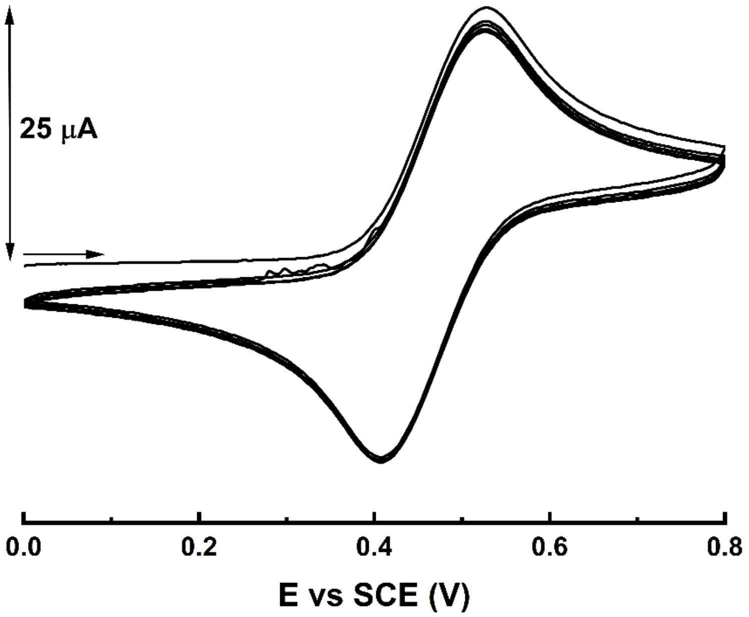

Figure S10: CVs of **4** (1 mM) in CH_2Cl_2 / $TBAPF_6$ 0.1 M after partial conversion to **4c** upon irradiation at 365 nm. 5 oxidation-reduction cycles at ν = 100 mV s^{-1}.

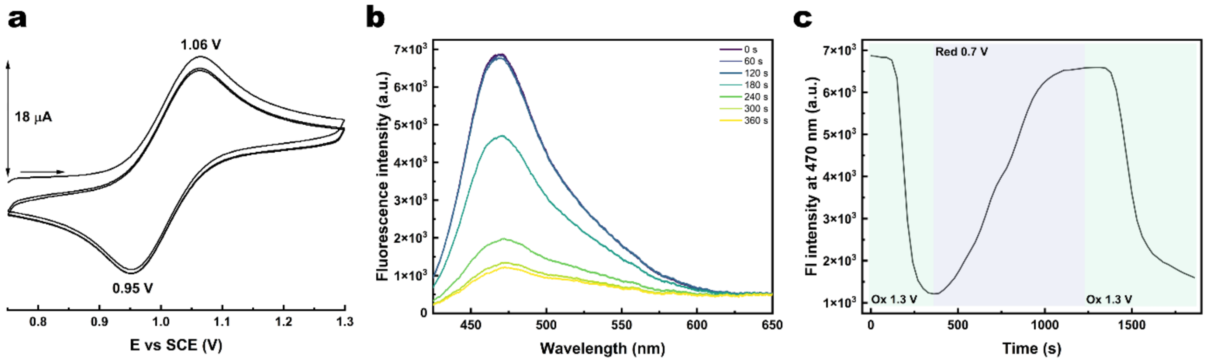

Figure S11: a) CV of intermediate **20** (1 mM) in CH_2Cl_2 / $TBAPF_6$ 0.1 M, 3 oxidation-reduction cycles at ν = 100 mV s^{-1}; b) Decrease of the emission band of intermediate **20** in CH_2Cl_2 / $TBAPF_6$ 0.1 M upon oxidation at 1.30 V vs SCE; c) Variation of the intensity at 470 nm of the emission band of intermediate **20** in CH_2Cl_2 / $TBAPF_6$ 0.1 M upon consecutive oxidation (1.30 V vs SCE) – reduction (0.70 V vs SCE) – oxidation (1.30 V vs SCE).

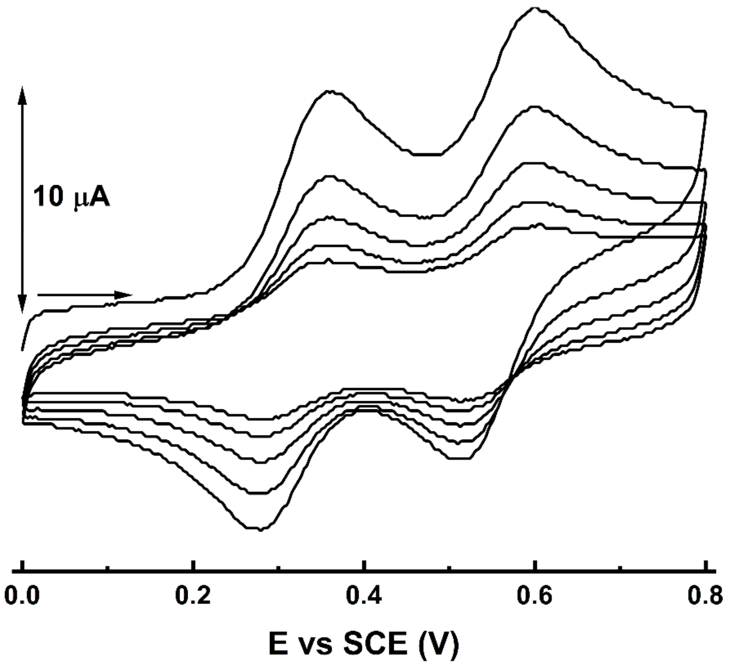

Figure S12: CVs of **2** (1 mM) in CH_2Cl_2 / $TBAPF_6$ 0.1 M after partial conversion to **2c** upon irradiation at 365 nm. 5 oxidation-reduction cycles at ν = 100 mV s^{-1}.

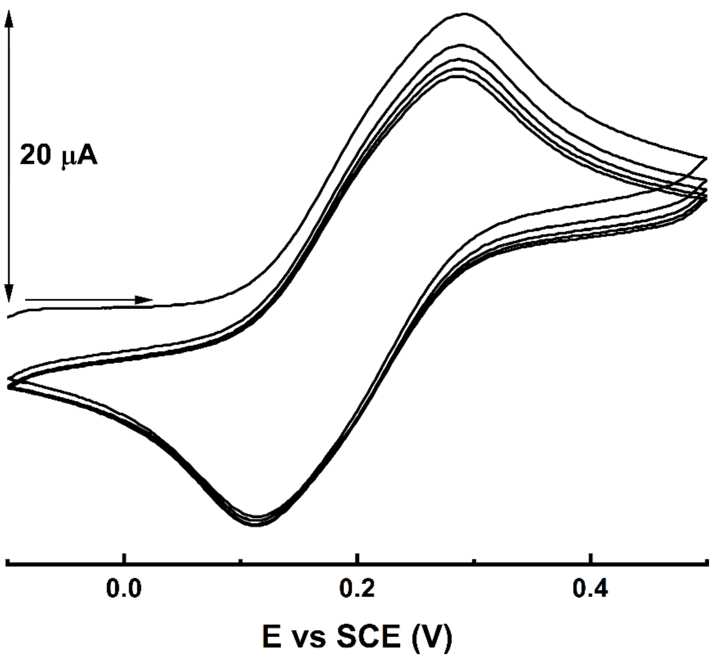

Figure S13: CVs of **3** (1 mM) in CH_2Cl_2 / $TBAPF_6$ 0.1 M after partial conversion to **3c** upon irradiation at 365 nm. 5 oxidation-reduction cycles at ν = 100 mV s^{-1}.

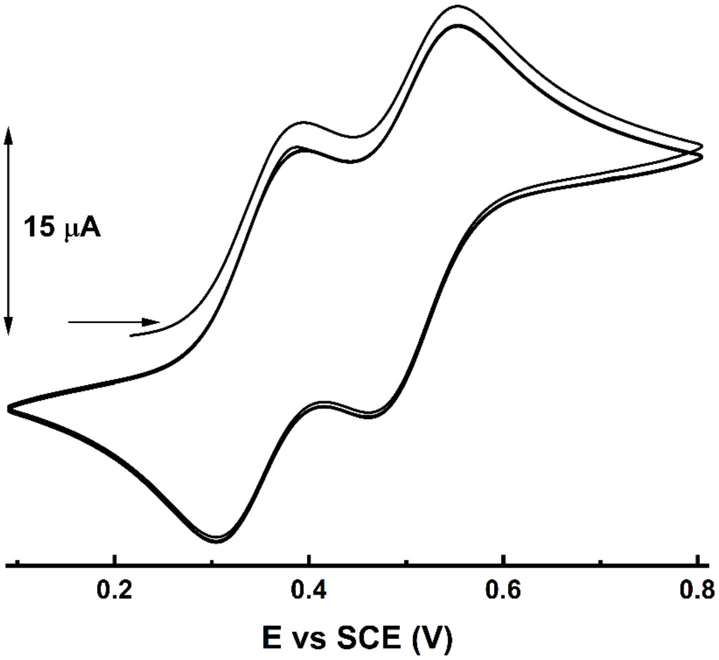

Figure S14: CVs of **5** (1 mM) in CH_2Cl_2 / $TBAPF_6$ 0.1 M after partial conversion to **5c** upon irradiation at 365 nm. 3 oxidation-reduction cycles at ν = 100 mV s^{-1}.

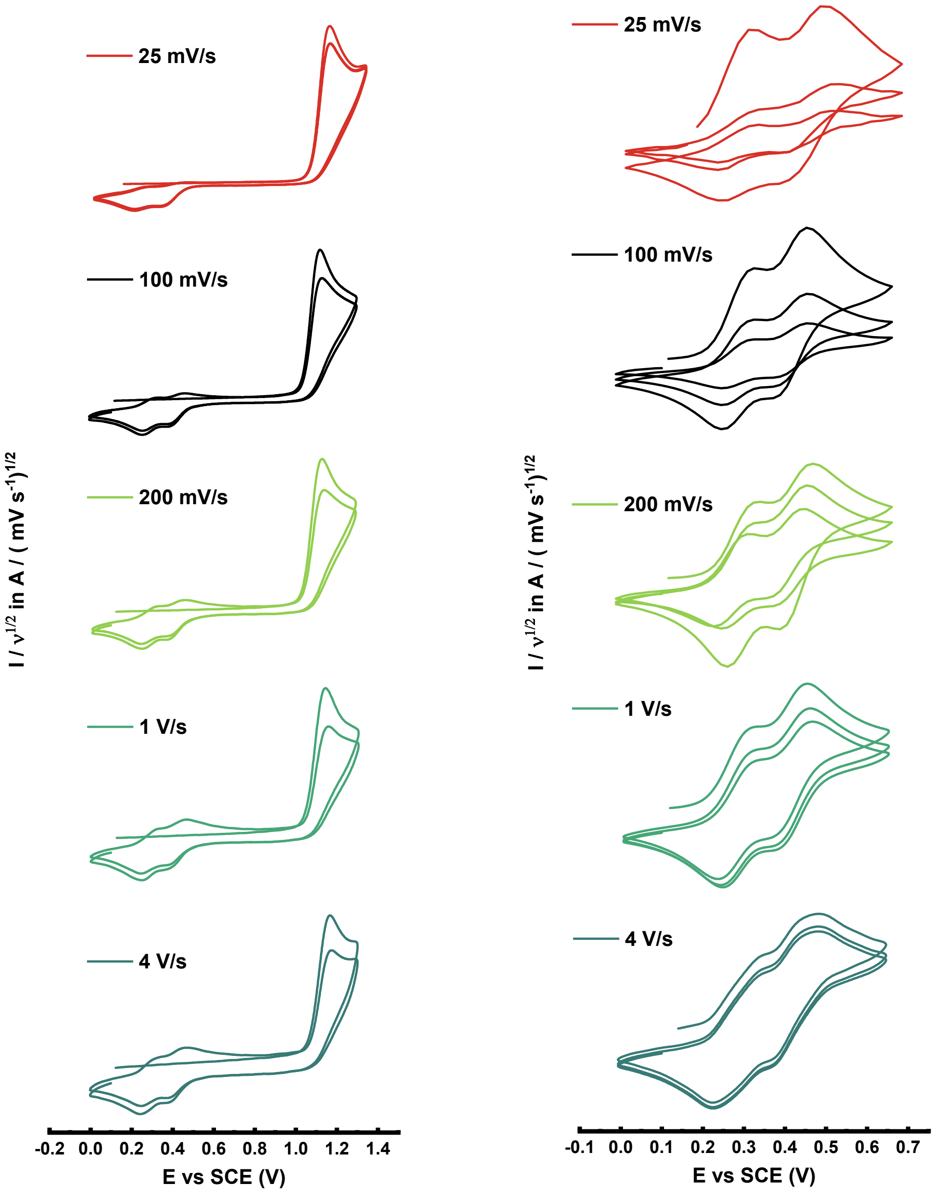

Figure S15: CVs of **1** (1 mM) in CH_3CN / $TBAPF_6$ 0.1 M at different scan rates. Left) before irradiation at 365 nm. Right) after irradiation at 365 nm.

Additional spectroelectrochemical data:

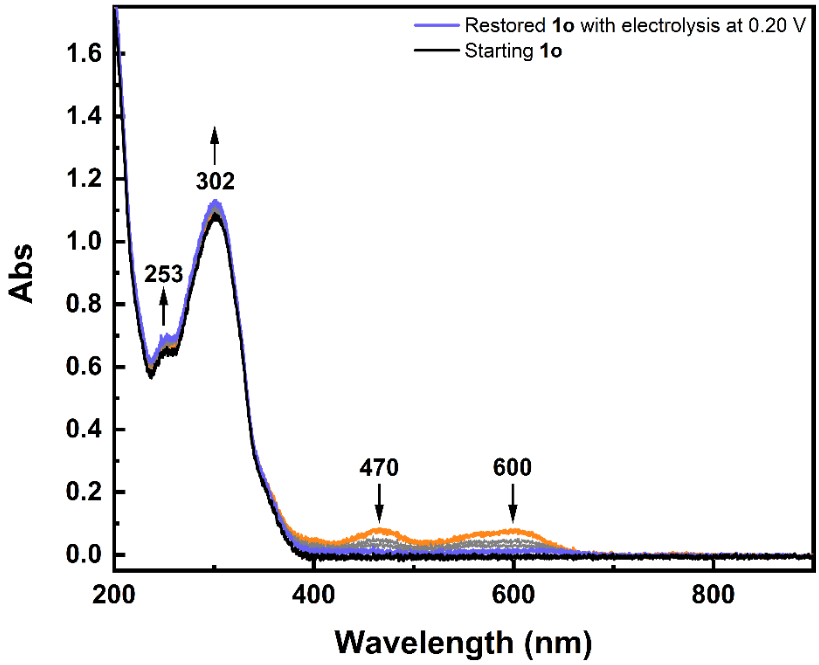

Figure S16: Spectral evolution of **1c**$^{2+}$ (2.27×10^{-4} M) in CH_3CN / $TBAPF_6$ 0.1 M under reduction at 0.20 V at RT. Optical path of the cuvette: 1 mm.

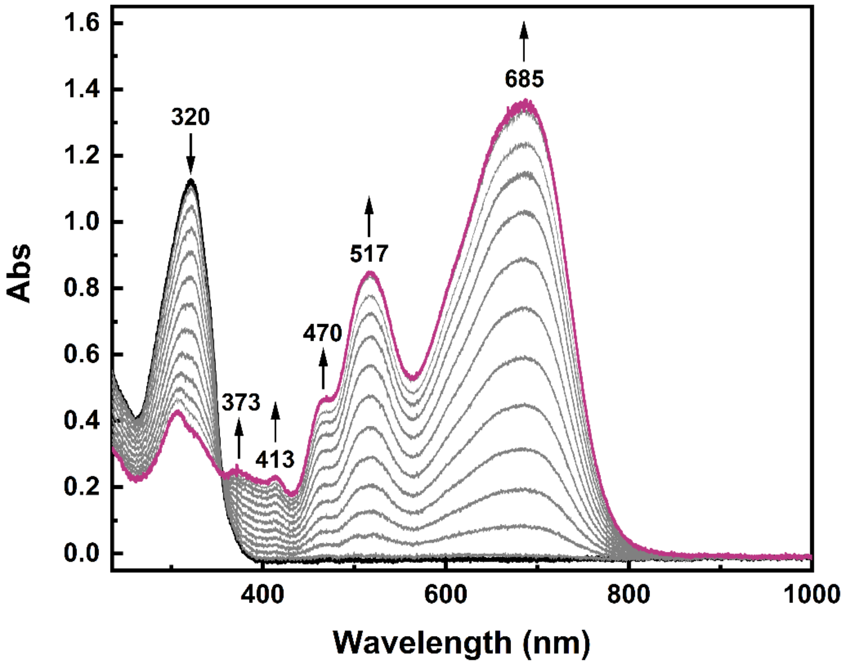

Figure S17: Spectral evolution of **2o** (5.38×10^{-4} M) in CH_2Cl_2 / $TBAPF_6$ 0.1 M under oxidation at 1.30 V at RT. Optical path of the cuvette: 1 mm.

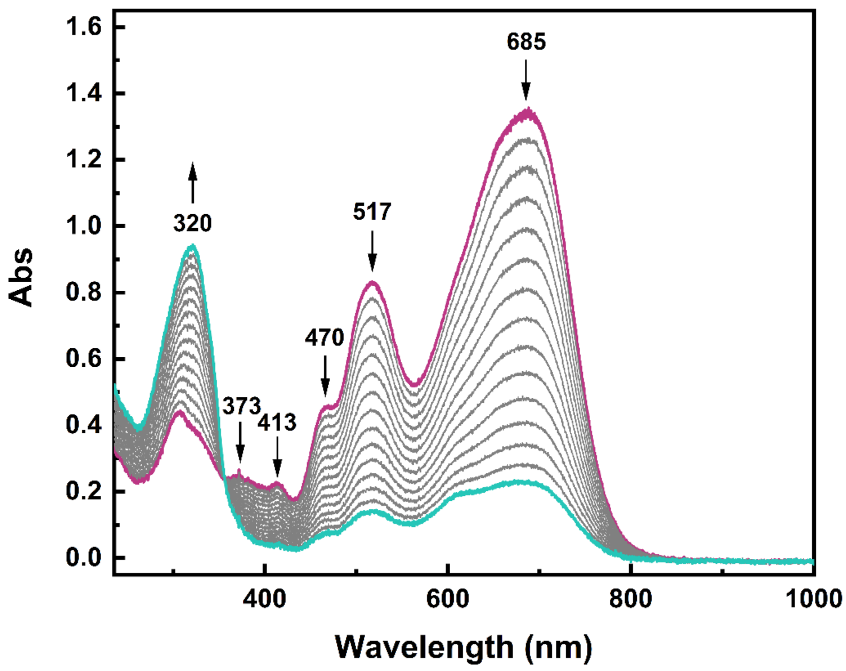

Figure S18: Spectral evolution of **2c**$^{2+}$ (5.38×10^{-4} M) in CH_2Cl_2 / $TBAPF_6$ 0.1 M under reduction at 0.55 V at RT. Optical path of the cuvette: 1 mm.

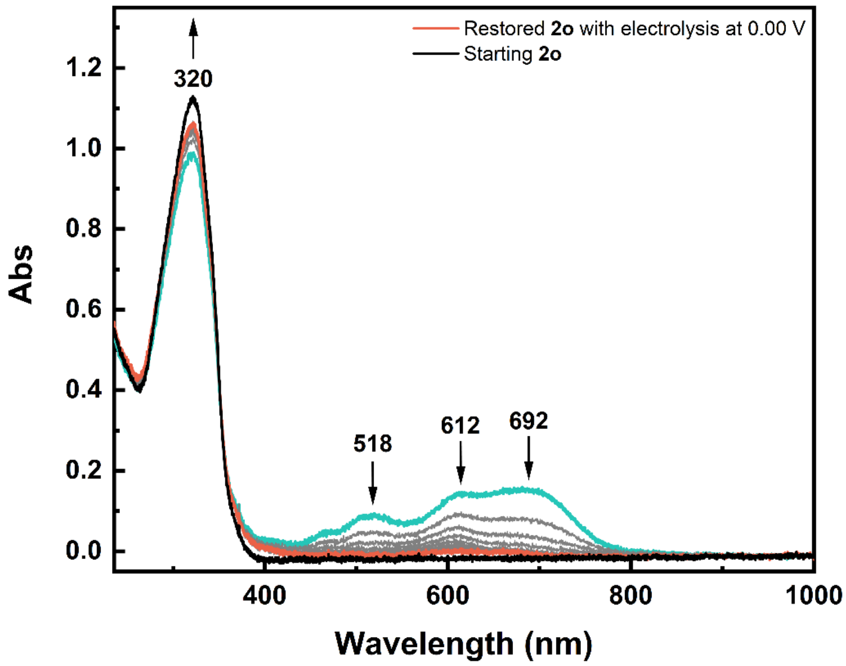

Figure S19: Spectral evolution of **2c**$^{2+}$ (5.38×10^{-4} M) in CH_2Cl_2 / $TBAPF_6$ 0.1 M under reduction at 0.00 V at RT. Optical path of the cuvette: 1 mm.

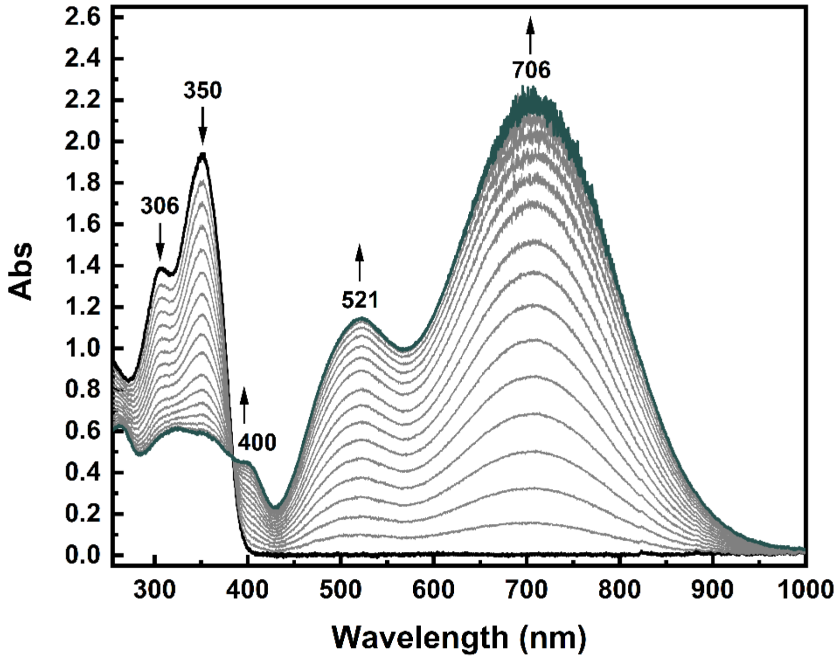

Figure S20: Spectral evolution of **3o** (3.17×10^{-4} M) in CH_2Cl_2 / $TBAPF_6$ 0.1 M under oxidation at 1.00 V at RT. Optical path of the cuvette: 1 mm.

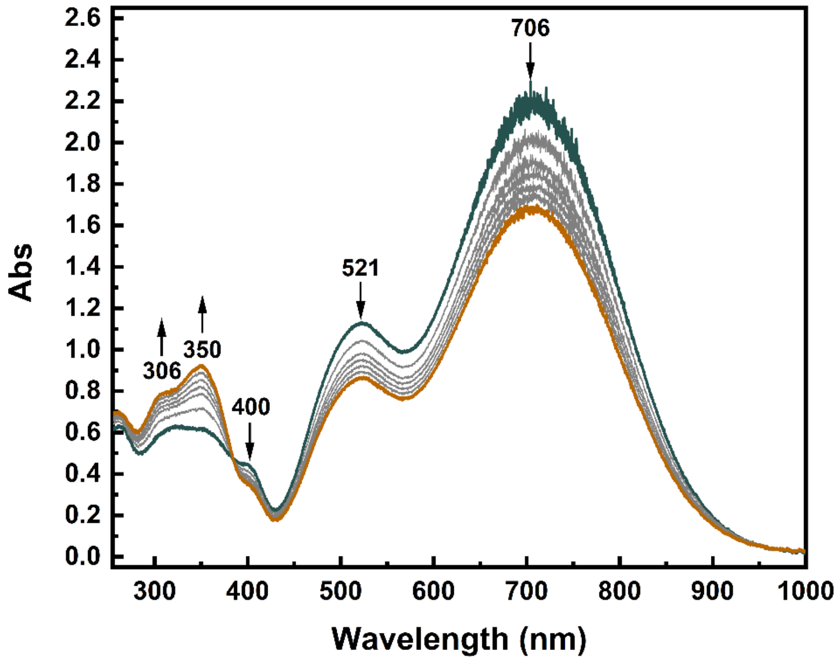

Figure S21: Spectral evolution of **3c^{2+}** (3.17×10^{-4} M) in CH_2Cl_2 / $TBAPF_6$ 0.1 M under reduction at 0.25 V at RT. Optical path of the cuvette: 1 mm.

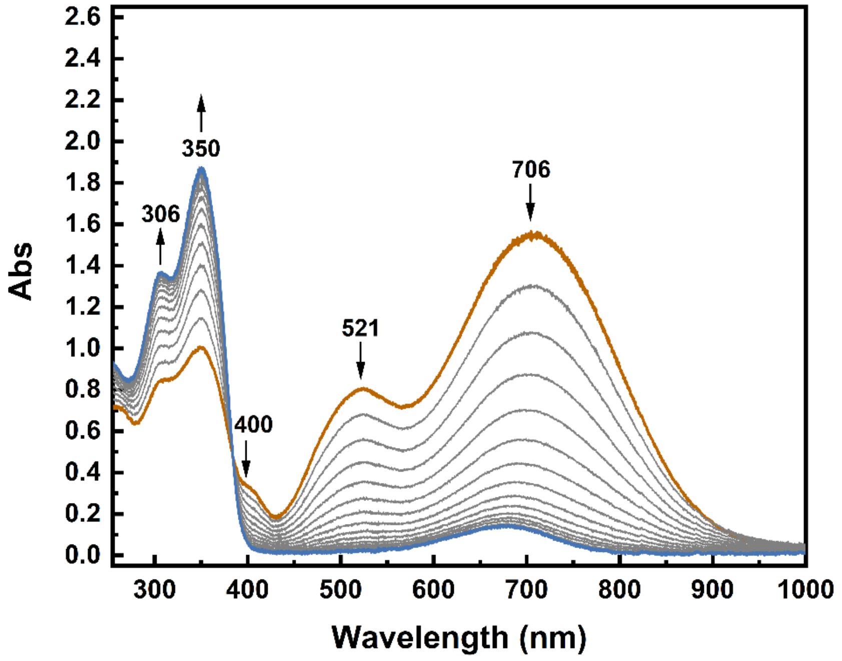

Figure S22: Spectral evolution of $\mathbf{3c}^{2+}$ (3.17×10^{-4} M) in CH_2Cl_2 / $TBAPF_6$ 0.1 M under reduction at 0.00 V at RT. Optical path of the cuvette: 1 mm.

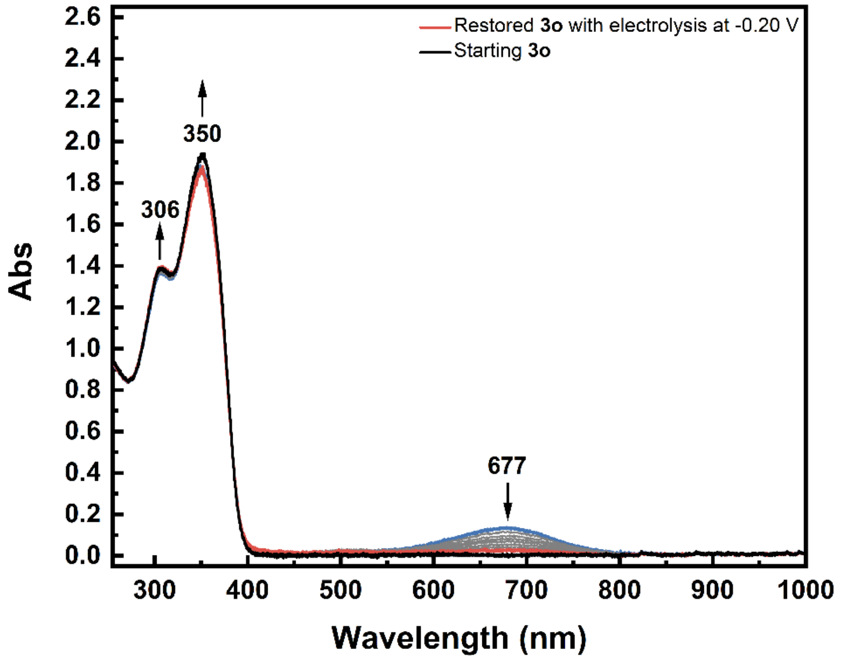

Figure S23: Spectral evolution of $\mathbf{3c}^{2+}$ (3.17×10^{-4} M) in CH_2Cl_2 / $TBAPF_6$ 0.1 M under reduction at -0.20 V at RT. Optical path of the cuvette: 1 mm.

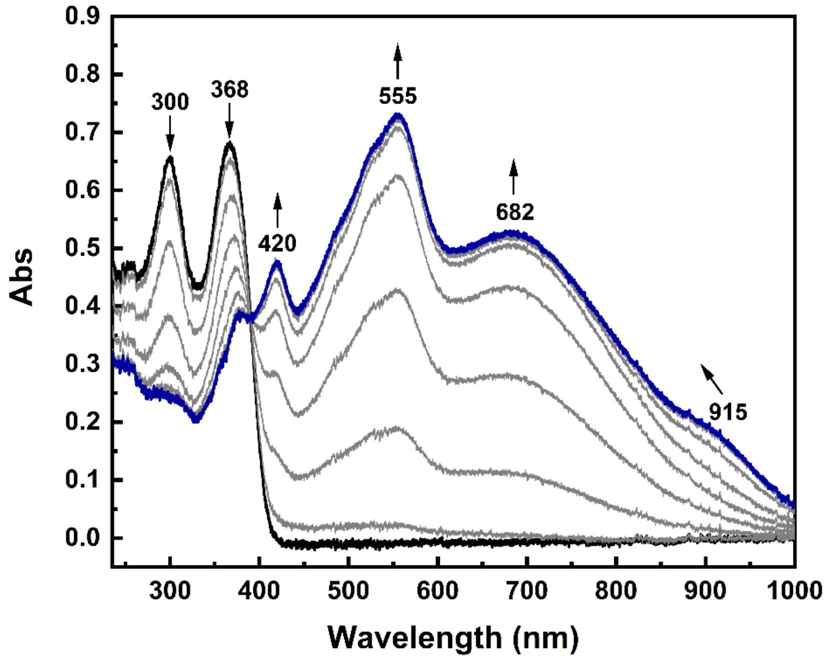

Figure S24: Spectral evolution of **5o** (2.25×10^{-4} M) in CH_2Cl_2 / $TBAPF_6$ 0.1 M under oxidation at 1.30 V at RT. Optical path of the cuvette: 1 mm.

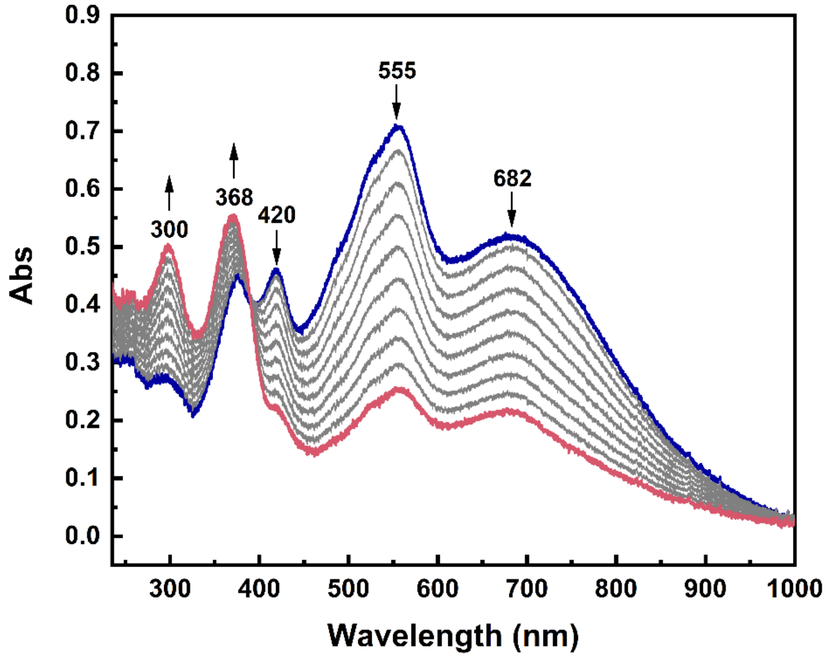

Figure S25: Spectral evolution of **5c**$^{2+}$ (2.25×10^{-4} M) in CH_2Cl_2 / $TBAPF_6$ 0.1 M under reduction at 0.45 V at RT. Optical path of the cuvette: 1 mm.

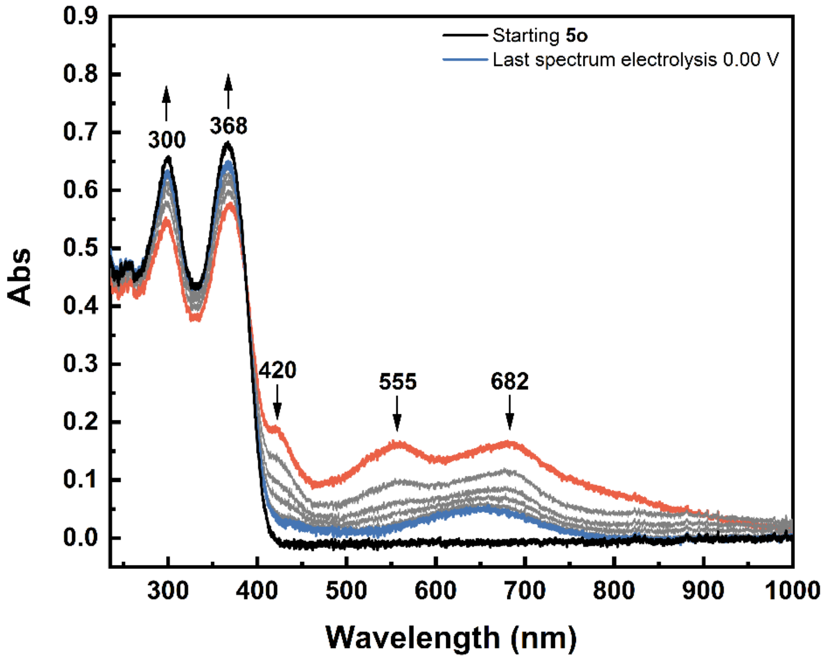

Figure S26: Spectral evolution of $\mathbf{5c}^{2+}$ (2.25×10^{-4} M) in CH_2Cl_2 / $TBAPF_6$ 0.1 M under reduction at 0.00 V at RT. Optical path of the cuvette: 1 mm.

Stopped-flow measurements and chemical red/ox experiments:

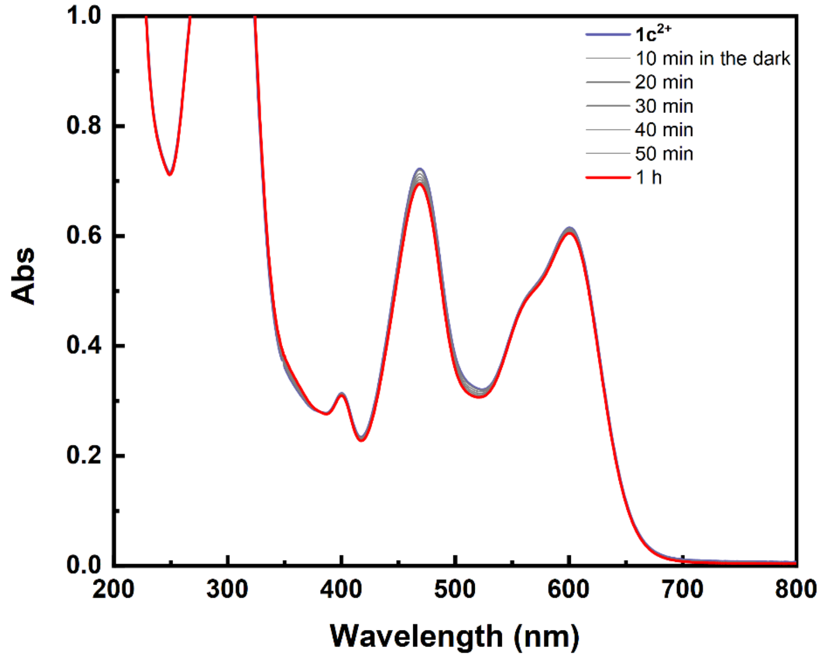

Figure S27: Thermal stability of $\mathbf{1c}^{2+}$ obtained by chemical oxidation of **1o** $1.92\text{x}10^{-5}$ M with 2 eq of “magic blue” in CH_3CN in the dark at room temperature. Optical path of the cuvette: 1 cm.

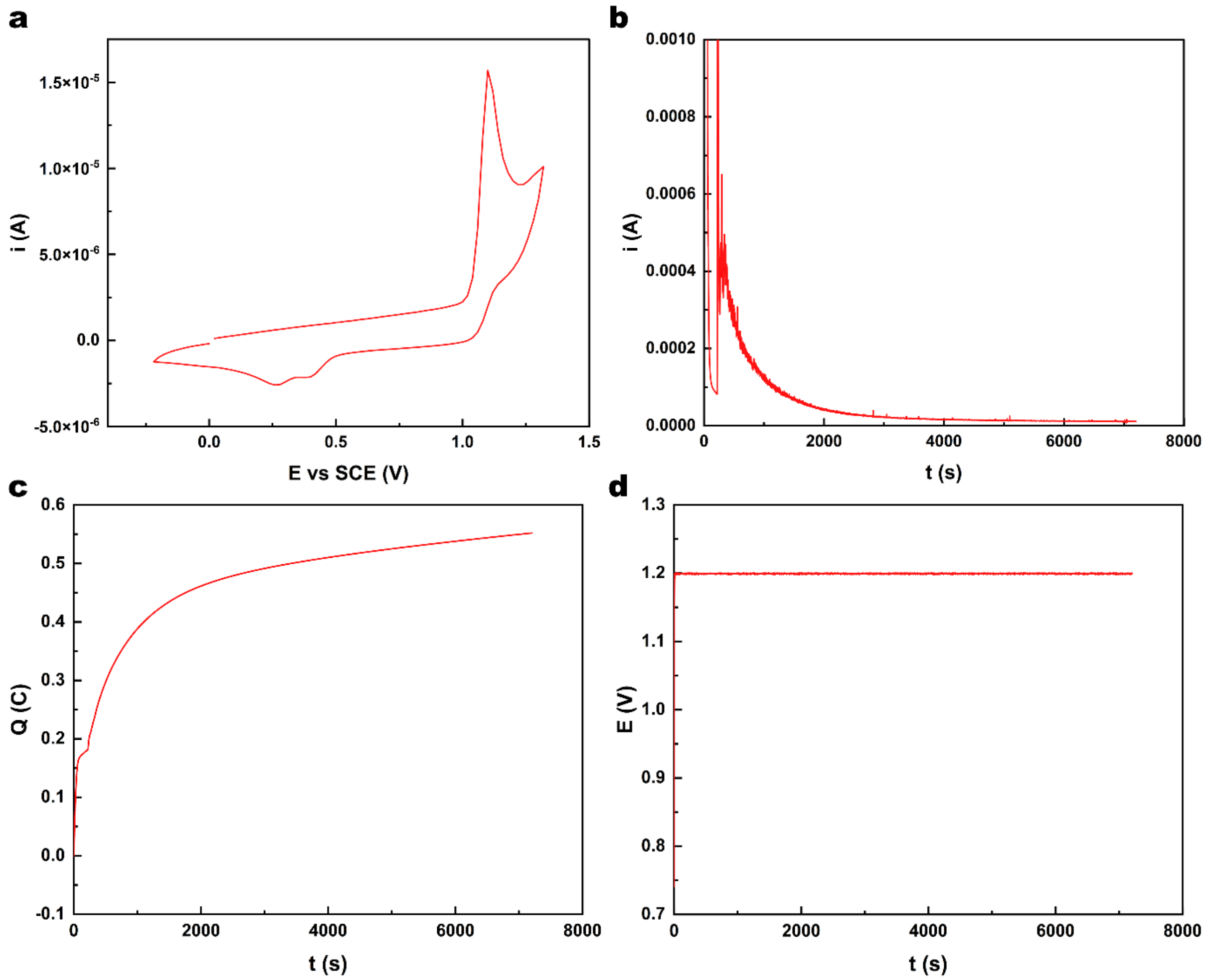

Figure S28: a) CV of **1o** in CH_3CN / $TBAPF_6$ 0.1 M in the electrolysis setup, ν = 100 mV s^{-1}, before the two-hour oxidation to prepare **1c**$^{2+}$; b) evolution of i (A) vs t (s) during the two-hour oxidation at 1.2 V vs SCE; c) evolution of Q (C) vs t (s) during the two-hour oxidation at 1.2 V vs SCE; d) evolution of E (V) vs t (s) during the two-hour oxidation at 1.2 V vs SCE.

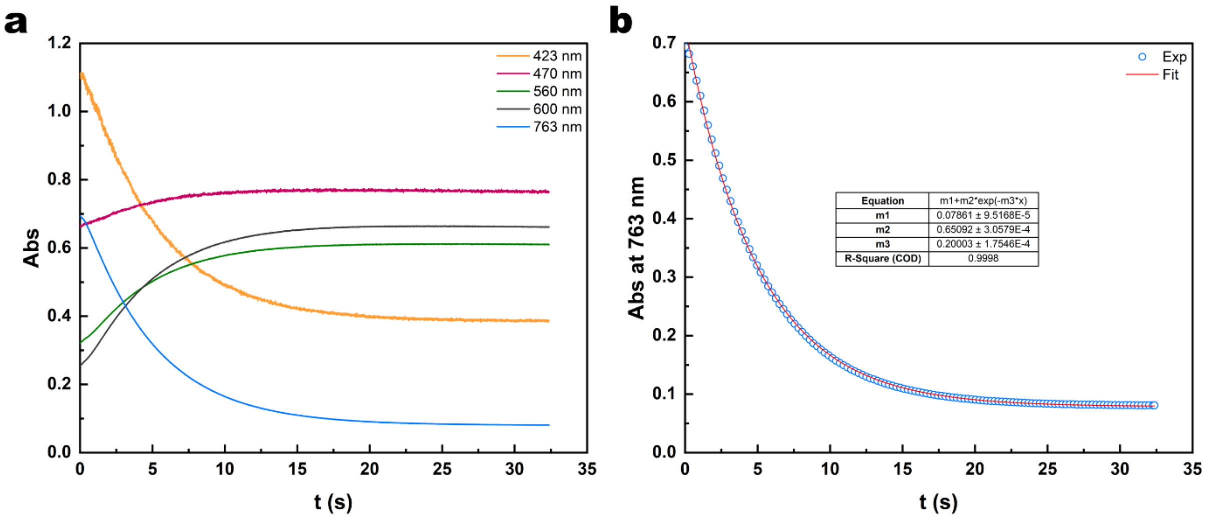

Figure S29: a) time traces at 423 nm, 470 nm, 560 nm, 600 nm and 763 nm for the stopped flow experiment monitoring the reduction of a **1c**$^{2+}$ solution (2.27×10^{-5} M) with a $Me_{10}Fc$ solution (2.27×10^{-5} M) in CH_3CN; b) fit of the time trace at 763 nm with y = m1 + m2 × exp(-m3 × t).

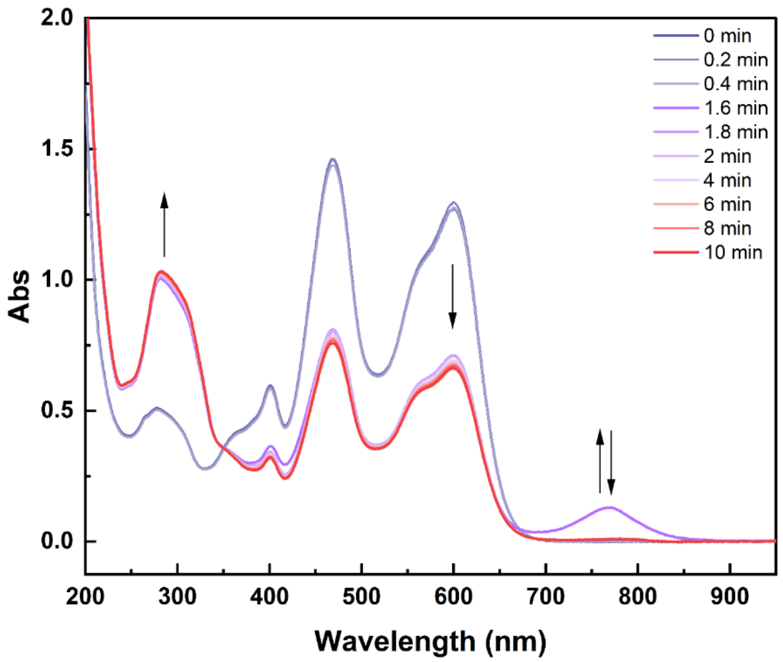

Figure S30: Spectral evolution after the addition of 1 eq of decamethylferrocene to **1c**$^{2+}$ (2.27×10^{-5} M, light purple solid line). Spectra were recorded after the addition of $Me_{10}Fc$ each 0.2 min. A selection of spectra is shown for the sake of clarity. The spectrum recorded after 10 min is provided with a red solid line and shows a 1:1 **1o** / **1c**$^{2+}$ composition. Optical path of the cuvette: 1 cm.

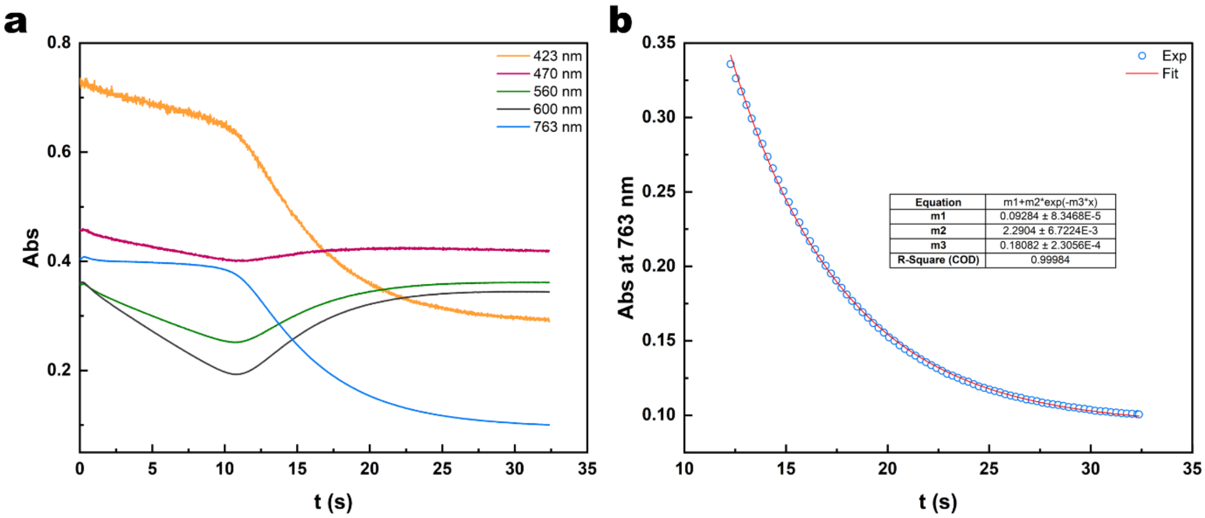

Figure S31: a) time traces at 423 nm, 470 nm, 560 nm, 600 nm and 763 nm for the stopped flow experiment monitoring the reduction of a **1c**$^{2+}$ solution (2.27×10^{-5} M) with a $Me_{10}Fc$ solution (4×10^{-5} M) in CH_3CN; b) fit of the time trace at 763 nm with y = m1 + m2 × exp(-m3 × t) for the decay after 10 s.

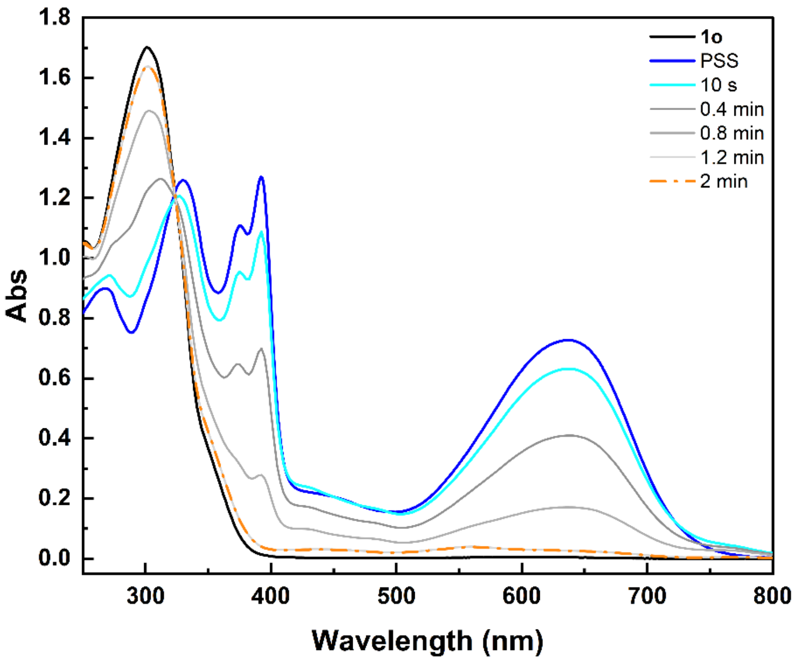

Figure S32: Spectral evolution after the addition of 0.10 eq of ferrocenium tetrafluoroborate to **1** at the photostationary state (blue solid line). The first spectrum recorded after the addition of Fc^+ (10 s) is provided with a light blue solid line. The following spectra were recorded every 0.4 min. The last spectrum at 2 min ca. is shown with an orange dashed-dotted line. Optical path of the cuvette: 1 cm.

Coupled electrolysis-EPR spectroscopy:

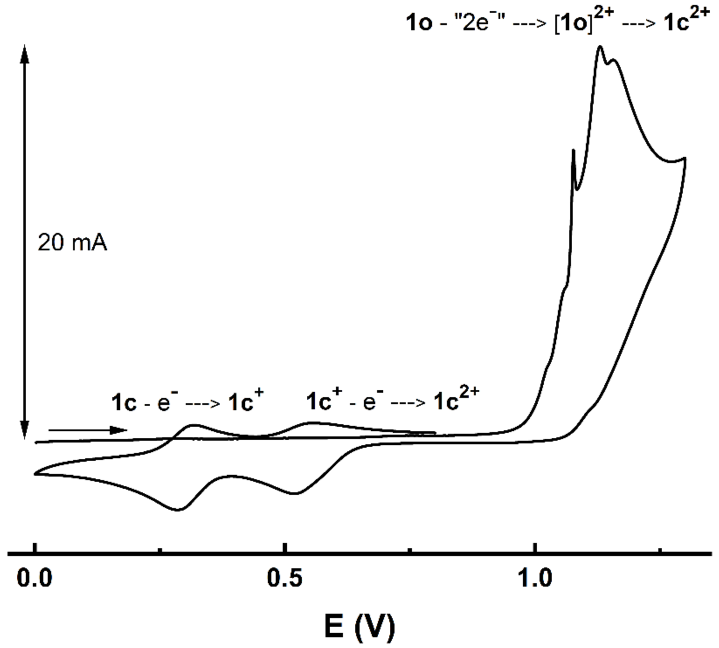

Figure S33: CV of **1o** (2.5 mM) in CH_2Cl_2 / $TBAPF_6$ 0.24 M with the *in-house* three-electrode setup inside an EPR tube. ν = 100 mV s^{-1}.

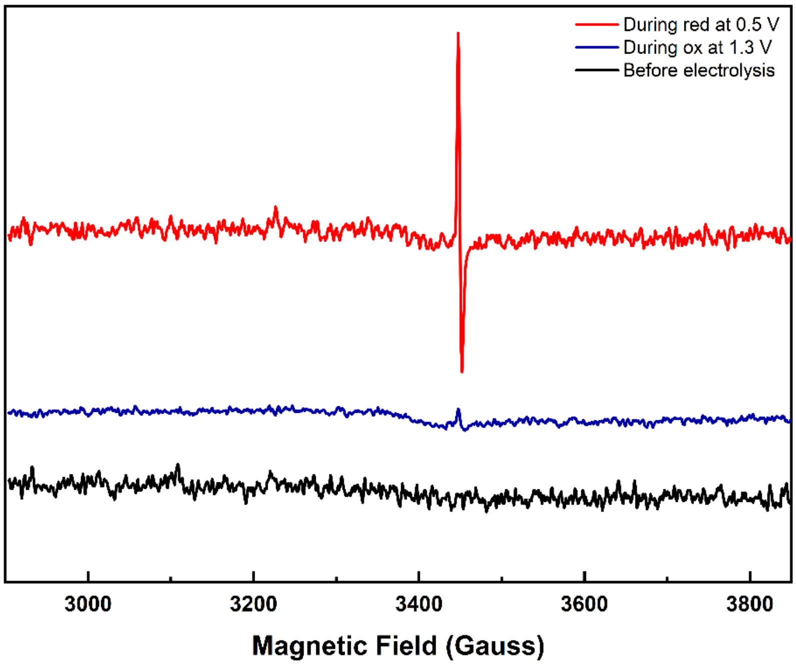

Figure S34: EPR spectra of **1** (2.5 mM) in CH_2Cl_2 / $TBAPF_6$ 0.24 M before the electrolysis, during the oxidation at 1.3 V and during the reduction at 0.5 V. Spectral acquisition time: 28 s.

Cyclic voltammetry simulations:

The parameters used in the simulations are presented in the following tables (Table S1 – S2).

To reduce the number of adjustable parameters, all electrochemical processes were considered with a α = 0.5 and the same electron transfer rate constant (i.e. k_s = 0.02 cm s^{-1}; the value of was determined by adjustment of the CV of **1c**).

It was assumed that all the involved species have the same diffusion coefficient, and its value was determined independently by DOSY-NMR. The data treatment to estimate the diffusion coefficient of **1o** is available in Figure S35. Ferrocene was added to the solution of **1o** in CD_3CN to have a reference. The exponential decays were fitted with the formula [Y = B+F*exp(-X*G)], where G is the diffusion coefficient in $cm^2\ s^{-1}$. The average of the G values for **1o** is 1.47×10^{-5} $cm^2\ s^{-1}$ and it was approximated to 1.5×10^{-5} $cm^2\ s^{-1}$ in the simulations for all the involved species. The diffusion coefficient for Fc is 2.64×10^{-5} $cm^2\ s^{-1}$, in agreement with reported values in the literature.[17]

The oxidation of **1o** was modelled with an EEC mechanism. Both electrochemical processes involving **1o** were arbitrarily set to the same potential while in reality they should occur at different potentials: either the second electron transfer is more difficult due to electrostatic constraints, or the second electron transfer is easier if a potential inversion phenomenon occurs. This consideration is beyond the scope of the present study. For the chemical step, again, only a lower value of the constant can be evaluated due to the limited exploration of the scan rate range. The value is thus estimated to be greater than 100 s^{-1}, but is likely to be faster, as suggested by Feringa and co-workers.

Regarding the inclusion of the disproportionation reaction of **1c**$^{+\bullet}$ in the simulations (light orange row in Tables S1 and S2) it should be noted that the thermodynamic constant is automatically set by the standard potential of each electron transfer, and that only a lower possible value of the rate constants may be estimated since above a certain value the contribution to the CV simulation is negligible, probably due to the fact that the reaction becomes too fast so that the process is limited by the diffusion rate of **1c** and **1c**$^{2+}$. The dismutation is estimated to be greater than 1000 $M^{-1}\ s^{-1}$ while the comproportionation is faster than 232000 $M^{-1}\ s^{-1}$.

On the other hand, adding a parasitic reaction causing an irreversible decomposition of **1c**$^{2+}$ (light blue row in Table S1) and arbitrarily adjusting its rate constant, allows a further refinement of the fit between experimental and simulated CVs. The occurrence of such a reaction could be considered reasonable since the degradation of redox-active DAEs in electrochemical conditions has been reported in the literature.[18,19]

Finally, the initial concentration of **1c** for the modelling of the CVs on such photogenerated species was estimated in such a way that the current for the simulated CVs could match the experimental one.

Table S1: Used parameters for the simulation of the CVs of **1o**. "=" is used for reversible reactions; "=>" is used for irreversible reactions.

Charge-Transfer reaction	Type	E° (V)	α / λ (eV)	k_s (cm s^{-1})
$\mathbf{1o}^{2+}$ + e = $\mathbf{1o}^{+}$	BV	1.1	0.5	0.02
$\mathbf{1o}^{+}$ + e = **1o**	BV	1.1	0.5	0.02
$\mathbf{1c}^{2+}$ + e = $\mathbf{1c}^{+}$	BV	0.42	0.5	0.02
$\mathbf{1c}^{+}$ + e = **1c**	BV	0.28	0.5	0.02

Chemical reaction	Keq	kf	kb
$\mathbf{1o}^{+}$ = $\mathbf{1c}^{+}$	20	2	0.1
$\mathbf{1o}^{+}$ + $\mathbf{1c}^{+}$ => **1o** + $\mathbf{1c}^{2+}$	/	> 5×10^{6}	0
$\mathbf{1o}^{+}$ + **1c** => **1o** + $\mathbf{1c}^{+}$	/	> 9×10^{6}	0
$\mathbf{1c}^{+}$ + $\mathbf{1c}^{+}$ = $\mathbf{1c}^{2+}$ + **1c**	0.004305	> 1000	> 2.32×10^{5}
$\mathbf{1o}^{2+}$ => $\mathbf{1c}^{2+}$	/	> 100	0
$\mathbf{1c}^{2+}$=> **R**	/	0.003	0

Species	Boundary	D (cm^2 s^{-1})	Cinit (mol L^{-1})
**1o**	ORB	1.5×10^{-5}	0.001
$\mathbf{1o}^{+}$	ORB	1.5×10^{-5}	0
$\mathbf{1o}^{2+}$	ORB	1.5×10^{-5}	0
**1c**	ORB	1.5×10^{-5}	0
$\mathbf{1c}^{+}$	ORB	1.5×10^{-5}	0

**1c**$^{2+}$	ORB	1.5×10^{-5}	0

Table S2: Used parameters for the simulation of the CVs of **1c**. "=" is used for reversible reactions; "=>" is used for irreversible reactions.

Charge-Transfer reaction	Type	E° (V)	α / λ (eV)	Ks (cm s^{-1})
**1c**$^{2+}$ + e = **1c**$^{+}$	BV	0.42	0.5	0.02
**1c**$^{+}$ + e = **1c**	BV	0.28	0.5	0.02

Chemical reaction	Keq	kf	kb
**1o**$^{+}$ = **1c**$^{+}$	20	2	0.1
**1o**$^{+}$ + **1c**$^{+}$ => **1o** + **1c**$^{2+}$	/	> 5×10^{6}	0
**1o**$^{+}$ + **1c** => **1o** + **1c**$^{+}$	/	> 9×10^{6}	0
**1c**$^{+}$ + **1c**$^{+}$ = **1c**$^{2+}$ + **1c**	0.004305	> 1000	> 2.32×10^{5}

Species	Boundary	D (cm^2 s^{-1})	Cinit (mol L^{-1})
**1o**	ORB	1.5×10^{-5}	0.00064
**1o**$^{+}$	ORB	1.5×10^{-5}	0
**1o**$^{2+}$	ORB	1.5×10^{-5}	0
**1c**	ORB	1.5×10^{-5}	0.00036
**1c**$^{+}$	ORB	1.5×10^{-5}	0
**1c**$^{2+}$	ORB	1.5×10^{-5}	0

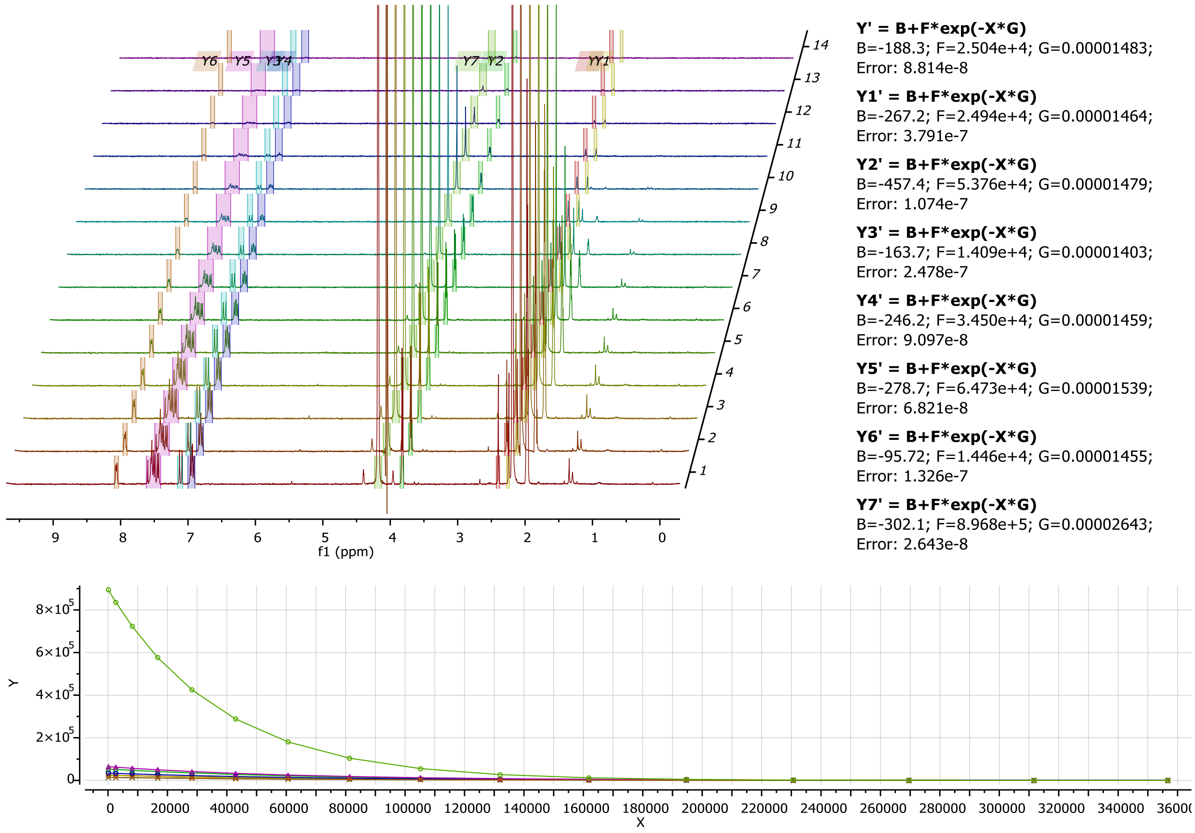

Figure S35: DOSY-NMR of **1o** + Fc in CD_3CN at 293 K and data fitting. The peak at 4.15 ppm is related to Fc.

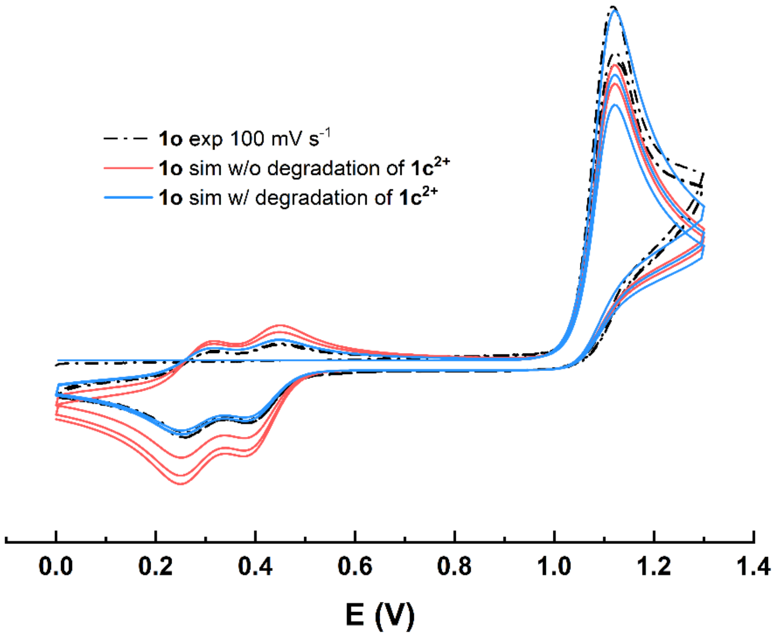

Figure S36: Comparison between the experimental CV of **1o** (black dashed-dotted line) and the simulated CVs with the parameters of Table S1, ν = 100 mV s^{-1}. A light red solid line is used

for the model without inclusion of the degradation reaction of **1c**$^{2+}$ while the simulation with its addition is provided with a light blue solid line.

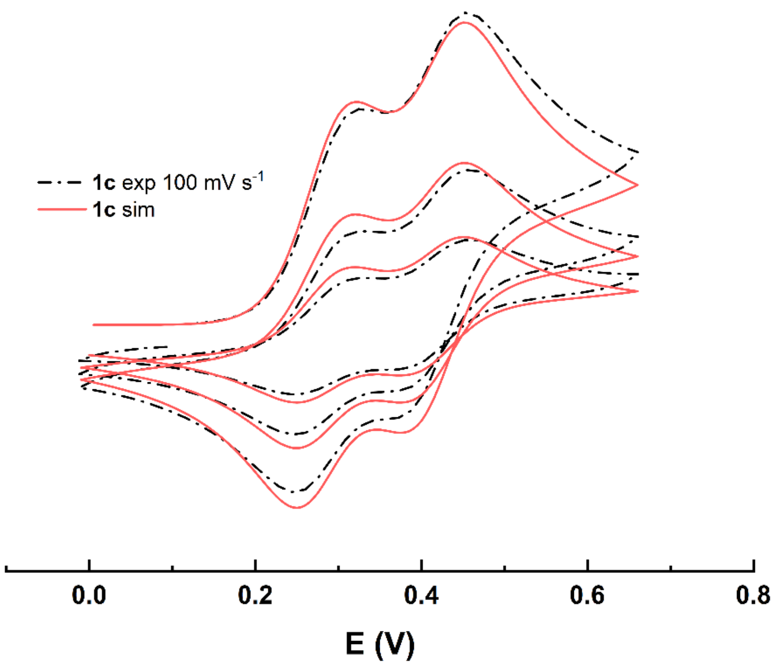

Figure S37: Comparison between the experimental CV of photogenerated **1c** (black dashed-dotted line) and the simulated CV (light red solid line) with the parameters of Table S2, ν = 100 mV s^{-1}.

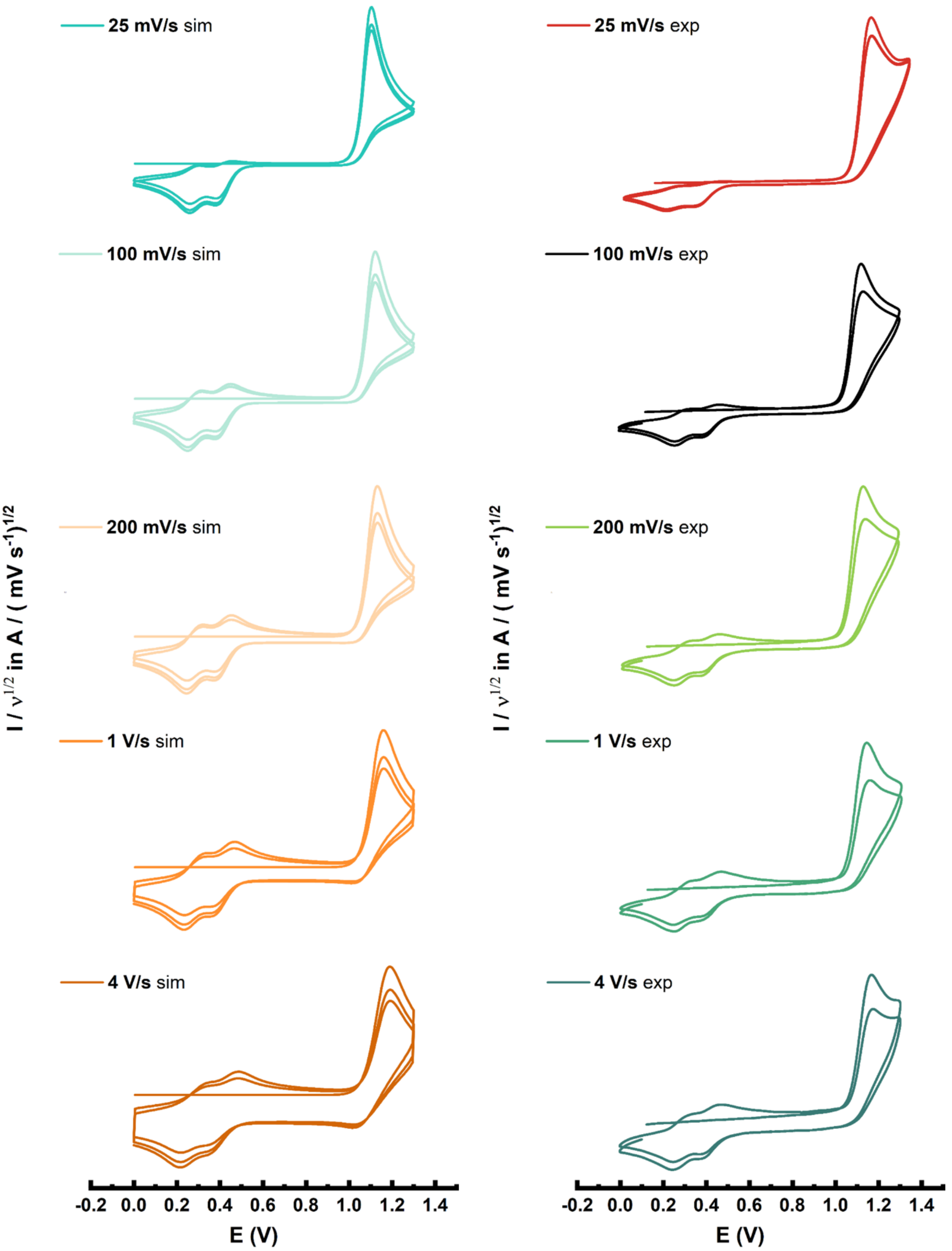

Figure S38: Simulated CVs (left) of **1o** with the parameters of Table S1 at different scan rates and experimental CVs (right) for comparison.

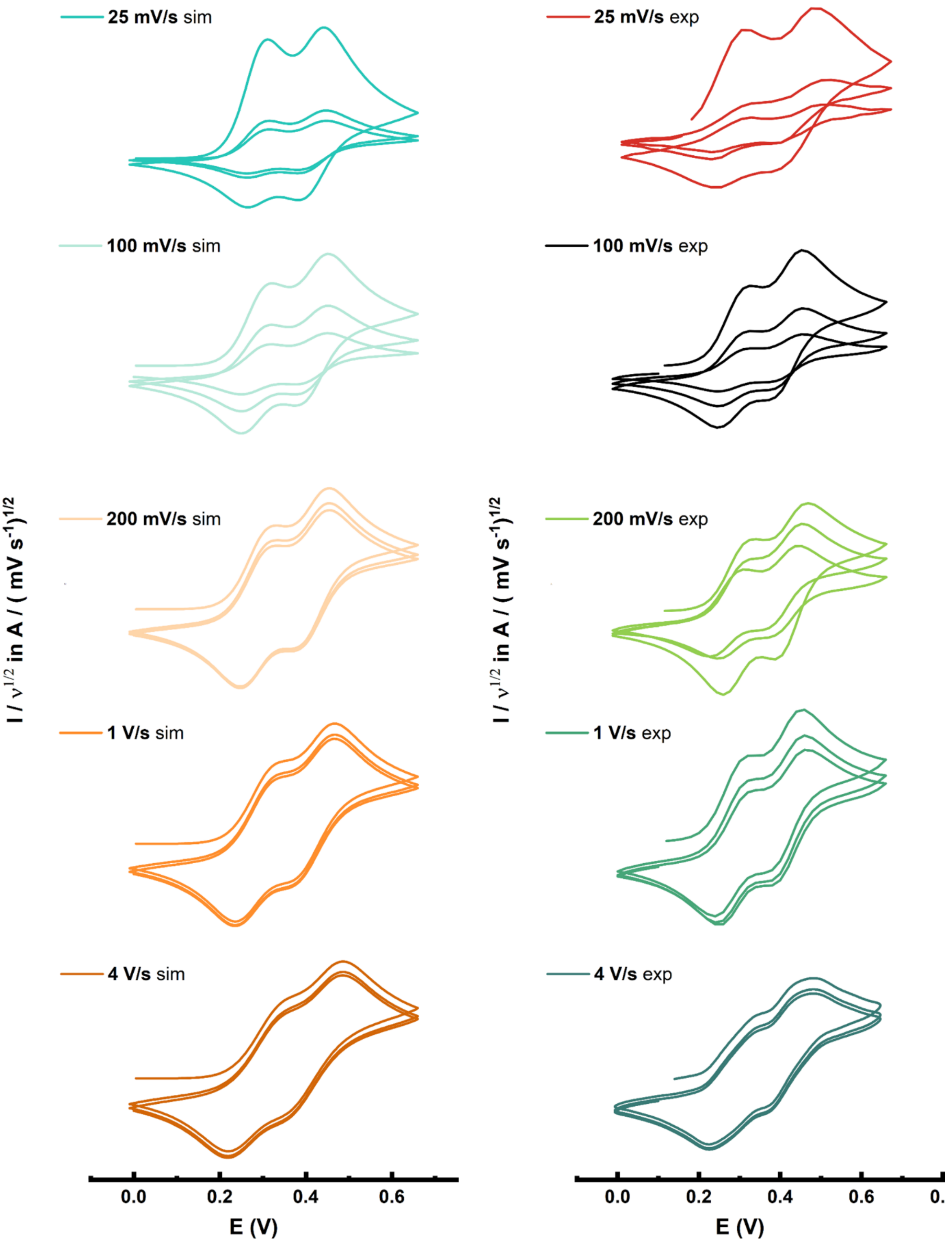

Figure S39: Simulated CVs (left) of **1c** with the parameters of Table S2 at different scan rates and experimental CVs (right) for comparison.

Time traces simulations:

First, the simulation of the decay at 763 nm was carried out for $\mathbf{1c}^{2+}$ (2.27×10^{-5} M) vs $Me_{10}Fc$ (2.27×10^{-5} M).

Expression of the simulated absorbance

It was assumed that only $\mathbf{1c}^{+\bullet}$ absorbed at this wavelength.
If $\mathbf{1c}^{2+}$ was fully converted to $\mathbf{1c}^{+\bullet}$ immediately after mixing (e.g. $[\mathbf{1c}^{+\bullet}]° = 2.27\times10^{-5}$ M), the absorbance = 0.7 at 763 nm gives an estimation of $\varepsilon(\mathbf{1c}^{+\bullet}$, 763 nm) = 30800 $M^{-1}cm^{-1}$.
Abs (763nm) was expressed as Abs (763nm) = $[\mathbf{1c}^{+\bullet}] \times \varepsilon(\mathbf{1c}^{+\bullet}$, 763 nm) + offset = $[\mathbf{1c}^{+\bullet}] \times 30800 + 0.076$.
The offset value was evaluated from the absorbance at the end of the decay at 763 nm.

Mechanism

The mechanism was simulated with the following reactions:

$$1c^{\bullet+} \underset{k_{-1}}{\overset{k_1}{\rightleftharpoons}} 1o^{\bullet+}$$

$$1c^{\bullet+} + 1o^{\bullet+} \underset{k_{-2}}{\overset{k_2}{\rightleftharpoons}} 1c^{2+} + 1o$$

$$1c + 1o^{\bullet+} \underset{k_{-3}}{\overset{k_3}{\rightleftharpoons}} 1o + 1c^{\bullet+}$$

$$1c^{\bullet+} + 1c^{\bullet+} \underset{k_{-4}}{\overset{k_4}{\rightleftharpoons}} 1c + 1c^{2+}$$

Reactions (2) and (3) were considered totally displaced and the backward rates (i.e. k_{-2} and k_{-3}) were taken equal to zero to limit overparametrization. The forward and backward rates of reaction (4) were frozen to the optimal values determined for the CV simulations. The remaining parameters were adjusted to reproduce the evolution at 600 nm and 763 nm for the experiments with 1 eq $Me_{10}Fc$ and 1.8 eq $Me_{10}Fc$.

Initial concentrations

Assuming that immediately after mixing, $\mathbf{1c}^{2+}$ was fully converted to $\mathbf{1c}^{+\bullet}$, the concentration is supposed to be $[\mathbf{1c}^{+\bullet}]° = 2.27\times10^{-5}$ M).

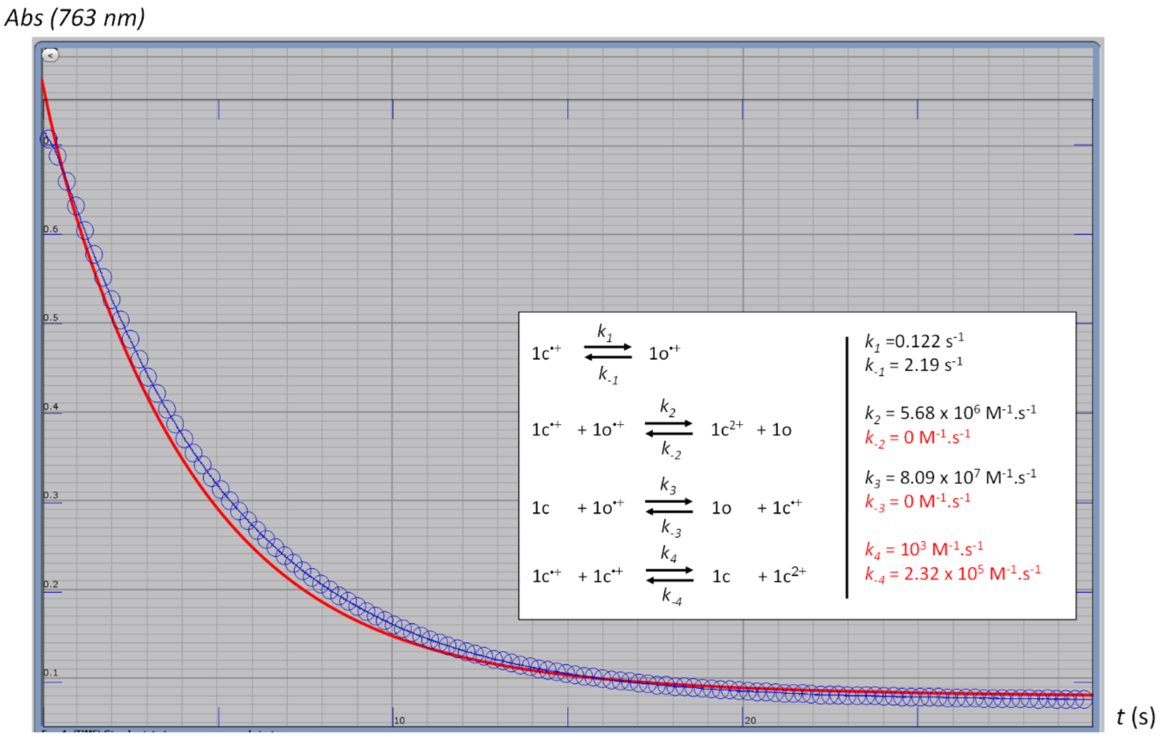

Figure S40: Best simulation (red solid line) obtained for the decay of the band at 763 nm observed upon mixing **1c**$^{2+}$ (2.27×10^{-5} M in CH_3CN containing 0.1 M $TBAPF_6$, generated by electrochemical oxidation of **1o**) and $Me_{10}Fc$ (2.27×10^{-5} M in CH_3CN). The estimated rate constants are provided in the white box. The frozen rate constants are written in red.

Next, the simulation of the time traces at 763 nm and 600 nm was done for **1c**$^{2+}$ (2.27×10^{-5} M) vs $Me_{10}Fc$ (4×10^{-5} M).

Expression of the simulated absorbance

1. At 763 nm:

It was assumed again that only **1c**$^{+\bullet}$ absorbed at 763 nm.

Abs (763nm) was expressed as

Abs (763nm) = [**1c**$^{+\bullet}$] × ε(**1c**$^{+\bullet}$, 763 nm) + offset = [**1c**$^{+\bullet}$] × 30800 + 0.095.

The offset value was evaluated from the absorbance at the end of the decay at 763 nm.

2. At 600 nm:

At 600 nm, **1c**, **1c**$^{2+}$ and **1c**$^{+\bullet}$ can absorb. For **1c** and **1c**$^{2+}$, the extinction coefficients were evaluated from the UV-vis spectra of these species and their absorbance at 600 nm (Figures 3 and 6a).

ε(**1c**, 600 nm) = 16200 M^{-1} cm^{-1}

ε(**1c**$^{2+}$, 600 nm) = 57200 M^{-1} cm^{-1}

For **1c**$^{+\bullet}$, ε was evaluated from the ratio between the absorbance of the radical at 600 nm and 763 nm.
ε(**1c**$^{+\bullet}$, 600 nm) = 9000 M^{-1} cm^{-1}
Abs (600nm) = [**1c**] × ε(**1c**$^{+\bullet}$, 600 nm) + [**1c**$^{2+}$] × ε(**1c**$^{+\bullet}$, 600 nm) + [**1c**$^{+\bullet}$] × ε(**1c**$^{+\bullet}$, 600 nm) + offset = [**1c**] × 16200 + [**1c**$^{2+}$] × 57200 + [**1c**$^{+\bullet}$] × 9000 + 0.055.
The offset was adjusted to fit the absorbance at 600 nm at the end of the evolution.

Mechanism

The mechanism was simulated again with the same reactions and the same assumptions:

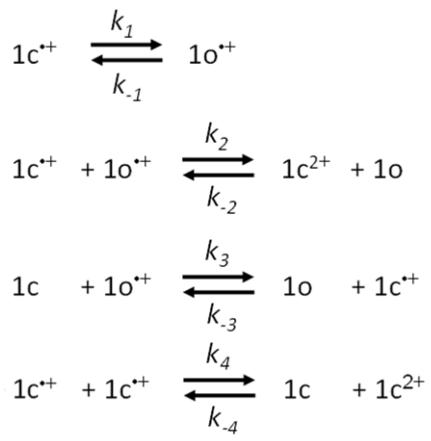

Initial concentrations

[**1c**$^{2+}$] = 2.27×10^{-5} M
[$Me_{10}Fc$]= 4×10^{-5} M

The following reactions take place instantaneously upon mixing:

1c^{2+} + $Me_{10}Fc$ ⇆ **1c**$^{+\bullet}$ + $Me_{10}Fc^+$
1c^{2+} + 2 $Me_{10}Fc$ ⇆ **1c** + 2 $Me_{10}Fc^+$

In the hypothesis where only these reactions have taken place at t = 0 and they are quantitative, we can estimate:

[**1c**]° = 1.73×10^{-5} M
[**1c**$^{+\bullet}$]° = 0.57×10^{-5} M
(2 × [**1c**]° + [**1c**$^{+\bullet}$]° = 4×10^{-5} M and [**1c**]° + [**1c**$^{+\bullet}$]° = 2.27×10^{-5} M)

But given that the reactions mentioned in the mechanism must take place simultaneously, **1c** will be partially converted to **1c**$^{+•}$. The experimental data was better simulated with the following initial concentrations:

[**1c**]° = 1.363×10^{-5} M
[**1c**$^{+•}$]° = 1.027×10^{-5} M

The overall initial concentration had to be increased to 2.39×10^{-5} M for optimal simulation.

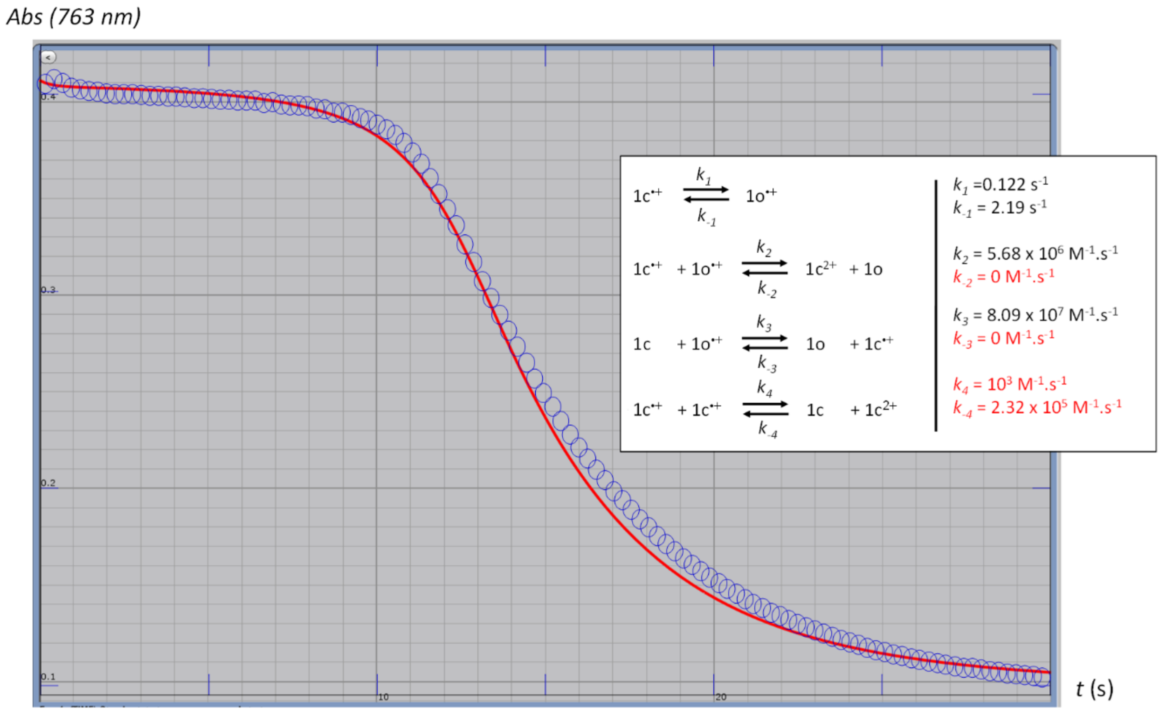

Figure S41: Best simulation (red solid line) obtained for the decay of the band at 763 nm observed upon mixing **1c**$^{2+}$ (2.27×10^{-5} M in CH_3CN containing 0.1 M $TBAPF_6$, generated by electrochemical oxidation of **1o**) and $Me_{10}Fc$ (4×10^{-5} M in CH_3CN). Initial concentrations for this simulation: [**1c**]° = 1.363×10^{-5} M and [**1c**$^{+•}$]° = 1.027×10^{-5} M. The estimated rate constants are provided in the white box. The frozen rate constants are written in red.

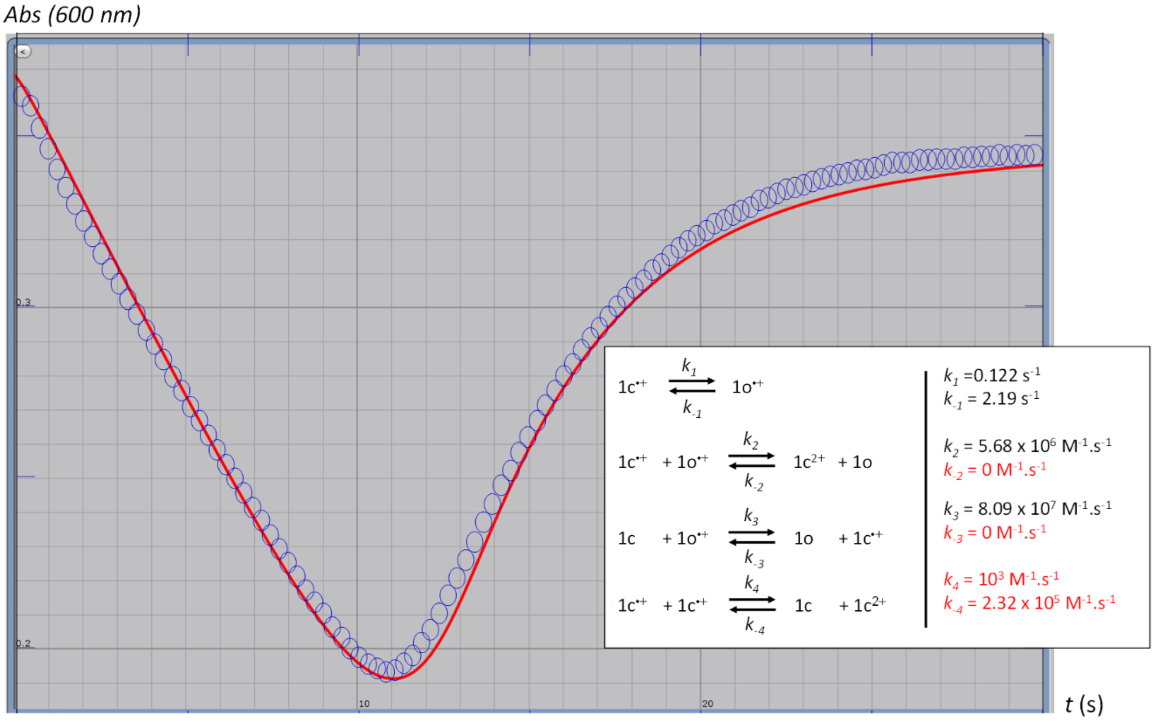

Figure S42: Best simulation (red solid line) obtained for the evolution of the band at 600 nm observed upon mixing **1c**$^{2+}$ (2.27×10^{-5} M in CH_3CN containing 0.1 M $TBAPF_6$, generated by electrochemical oxidation of **1o**) and $Me_{10}Fc$ (4×10^{-5} M in CH_3CN). Initial concentrations for this simulation: [**1c**]° = 1.363×10^{-5} M and [**1c**$^{+\bullet}$]° = 1.027×10^{-5} M. The estimated rate constants are provided in the white box. The frozen rate constants are written in red.

Additional calculation data:

Table S3: Aromatic energy (ωB97X-D/6-311G(d,p) calculations).

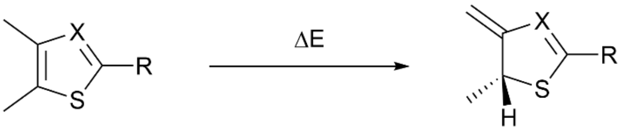

X	R	ΔE (kJ mol^{-1})
CH	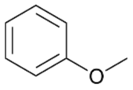	64.0
CH	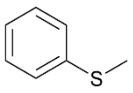	65.4
CH	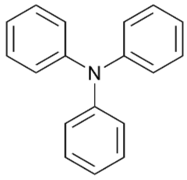	65.3
N	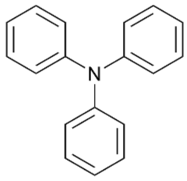	60.6

The aromatic stabilization of the aryl groups connected to the central ring of the photochromic compound was calculated using the reaction indicated in Table S3. The energy difference is computed between 60.6 and 65.4 kJ mol^{-1} which suggests that the large part of the energy difference between the OF and CF is due to aromatization of the side groups. This indicates that the aromaticity is the key value that controls the stability of the closed-ring isomers with respect to the open isomers.

Table S4: Relative energies (in kJ mol^{-1}) of the closed form isomers of **1** and **DTE** in neutral, radical and dicationic states at the ωB97X-D/6-311G(d,p) level of calculations. The values are calculated with respect to the energies of the antiparallel open form isomers in such redox states (0 kJ mol^{-1} for the sake of comparison).

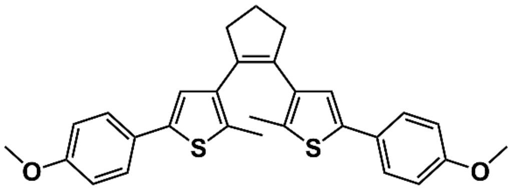

DTE

Redox state	1	DTE
**+0**		
$\Delta E_{CF\text{ - }OF}$	78.8	37.3
**+1**		
$\Delta E_{CF\text{ - }OF}$	-22.1	-46.9
**+2**		
$\Delta E_{CF\text{ - }OF}$	-100.2*	-109.6

* The antiparallel geometries of **1** in the open form dicationic state couldn't be obtained since the optimization converged to the corresponding dicationic closed forms. The relative energies of the closed forms were then calculated from the parallel conformation of the open forms.

*Selection of the best exchange-correlation functional for TD-DFT calculations: benchmark study on OF and CF forms of compound **1***

The calculated absorption maximum wavelength λ_{max}(calc) and the deviation from experimental value are listed in Table S4. Most of the λ_{max}(calc) values for the open- and closed-ring isomers differed significantly from the experimental absorption maximum wavelength (λ_{max}(exptl)) values by 0.05 eV or more. However, the λ_{max}(calc) values for the closed form **1c** estimated at the MPW1PW91/6-311G(d,p) level was 640 nm, in excellent agreement with the λ_{max}(exptl) = 636 nm. This functional performs also well for the **1o** which means it can describe excited states from the least to the most conjugated form in an extremely well-balanced way. In addition, the λmax(calc) values for the CF forms of compounds **2** – **5** did not greatly differ from the λ_{max}(exp) values (Table S5). Therefore, we concluded that TD-DFT calculations at the MPW1PW91/6-311G(d,p)//ωB97X-D/6-311G(d,p) level was the best choice to predict the absorption spectral properties of terarylenes **1** – **5**.

Table S5: Theoretical maximum absorption wavelength λ_{max}(calc) and deviation from experimental ($\Delta\lambda_{max}$ = λ_{max}(calc) - λ_{max}(exptl)) value calculated using different functionals for open (**1o**, antiparallel) and closed (**1c**) forms of compound **1**.

	**1o**			**1c**		
	λ_{max} (calc)	$\Delta\lambda_{max}$		λ_{max} (calc)	$\Delta\lambda_{max}$	
		nm	eV		nm	eV
PBE0/6-311G(d,p)	318	16	-0.21	759	123	-0.31
M06/6-311G(d,p)	355	53	-0.61	655	19	-0.06
**MPW1PW91/6-311G(d,p)**	305	3	**-0.04**	640	4	**-0.01**
M062X/6-311G(d,p)	304	2	-0.03	572	-64	0.22
CAM-B3LYP/6-311G(d,p)	312	10	-0.13	590	-46	0.15
ωB97X-D/6-311G(d,p)	300	-2	0.03	586	-50	0.17
MN15/6-311G(d,p)	323	11	-0.27	593	-43	0.14

Table S6: Theoretical maximum absorption wavelength λ_{max}(calc) and deviation from experimental ($\Delta\lambda_{max}$ = λ_{max}(calc) - λ_{max}(exptl)) value calculated for the closed forms of compounds **2** – **5** at the MPW1PW91/6-311G(d,p)//ωB97X-D /6-311G(d,p) level of calculations.

	**2**	**3**	**4**	**5**
Calc	652	677	633	642
Exptl	660	671	613	654
$\Delta\lambda_{max}$	-8	-6	20	-12

Table S7: Wavelengths of the three first absorption transitions and deviation from experimental (Δλ = λ(calc) - λ (exptl)) values calculated using different functionals for dicationic closed form of compound **1** (i.e. **1c**$^{2+}$).

	**λ(calc)**	**λ(exptl)**	**Δλ**
PBE0/6-311G(d,p)	569<br>450<br>404	600<br>470<br>400	-31<br>-20<br>4
M06/6-311G(d,p)	569<br>464<br>452	600<br>470<br>400	-31<br>-6<br>52
MPW1PW91/6-311G(d,p)	570<br>450<br>400	600<br>470<br>400	-30<br>-20<br>0
M062X/6-311G(d,p)	501<br>410<br>398	600<br>470<br>400	-99<br>-60<br>-2
CAM-B3LYP/6-311G(d,p)	496<br>405<br>391	600<br>470<br>400	-104<br>-65<br>-9
ωB97X-D/6-311G(d,p)	483<br>398<br>385	600<br>470<br>400	-117<br>-72<br>-15
MN15/6-311G(d,p)	531<br>427<br>413	600<br>470<br>400	-69<br>-43<br>13

Table S8: Computed energies of vertical electronic transitions (λ in nm), oscillator strength (*f*) and main orbitals (H = HOMO and L = LUMO) implied in the transition for **1o**, **1c**, **1c**$^{+•}$ and **1c**$^{2+}$.

	**λ (nm)**	***f***	**assignment**
**1o**			
$S_0 \rightarrow S_1$	357	0.1533	H → L
$S_0 \rightarrow S_2$	327	0.0303	H-1 → L
$S_0 \rightarrow S_3$	305	0.7579	H-2 → L
$S_0 \rightarrow S_4$	301	0.4792	H → L+1
**1c**			
$S_0 \rightarrow S_1$	640	0.4841	H → L
$S_0 \rightarrow S_2$	441	0.2385	H → L+1
$S_0 \rightarrow S_3$	397	0.4493	H-1 → L
**1c**$^{+•}$			
$S_0 \rightarrow S_1$	1235	0.1956	H(α) → L(α)
$S_0 \rightarrow S_2$	677	0.4472	H(β) → L(β)
$S_0 \rightarrow S_3$	573	0.0199	H-1(α) → L(α), H-1(β) → L(β)
$S_0 \rightarrow S_4$	533	0.0907	H-1(β) → L(β), H(α) → L+1(α)
$S_0 \rightarrow S_5$	531	0.0489	H-2(β) → L(β)
$S_0 \rightarrow S_6$	493	0.0070	H(α) → L+1(α)
$S_0 \rightarrow S_7$	440	0.0972	H(α) → L+1(α)
$S_0 \rightarrow S_8$	435	0.0915	H-2(α) → L (α)
$S_0 \rightarrow S_9$	430	0.0389	H-2(α) → L+2(α) H-3(β) → L(β)
$S_0 \rightarrow S_{10}$	423	0.2873	H-2(α) → L(α) H(β) → L+1(β)

**$1c^{2+}$**			
$S_0 \rightarrow S_1$	570	0.7464	H → L
$S_0 \rightarrow S_2$	472	0.0123	H-1 → L
$S_0 \rightarrow S_3$	450	0.6839	H → L+1
$S_0 \rightarrow S_4$	446	0.2148	H-2 → L
$S_0 \rightarrow S_5$	413	0.0181	H-3 → L
$S_0 \rightarrow S_6$	403	0.8923	H-1 → L+1

	1o
**L+1**	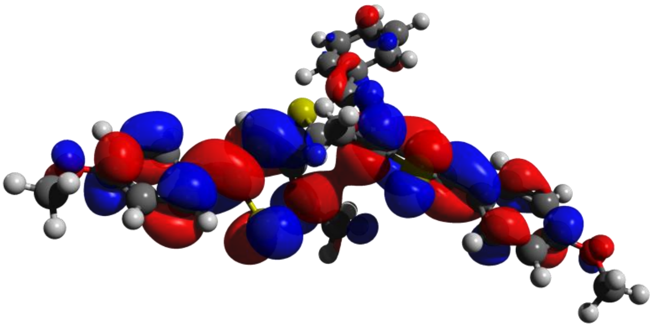
**L**	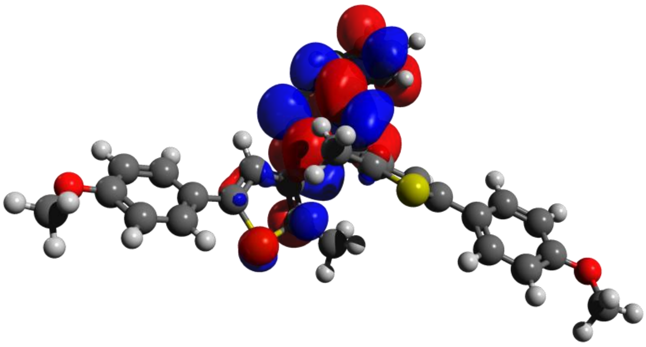
**H**	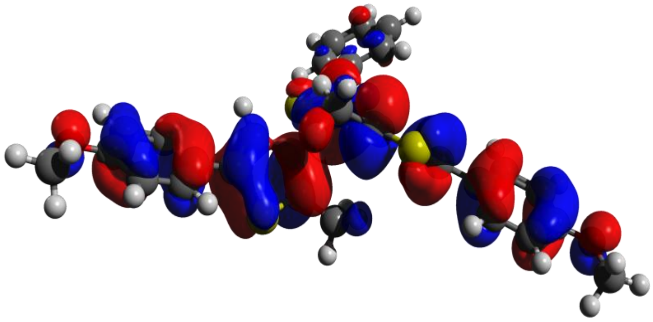
**H-1**	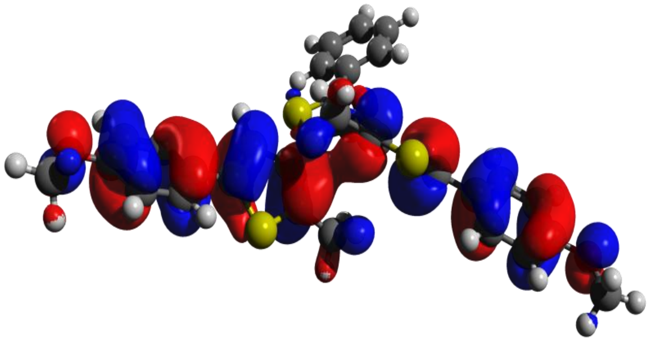

H-2

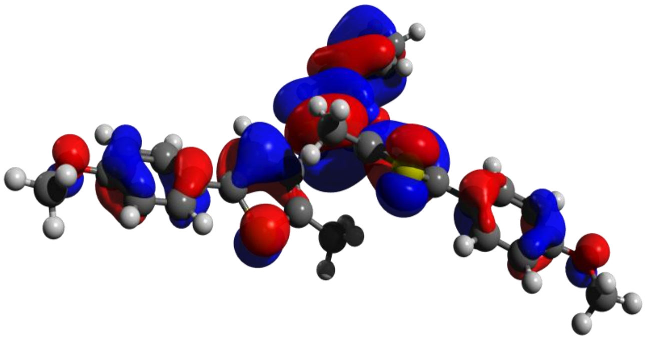

1c

L+1

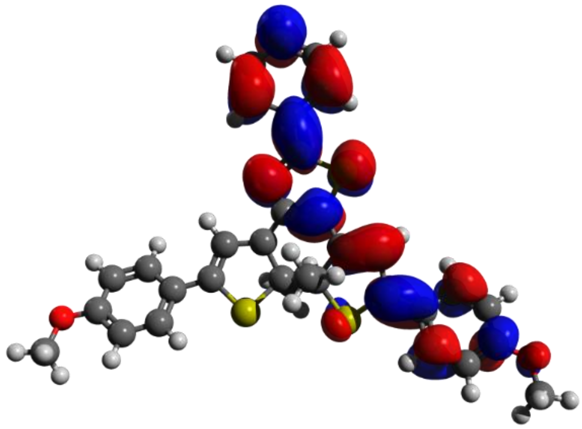

L

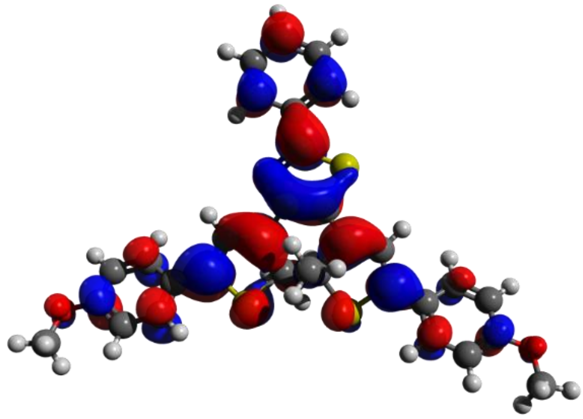

H

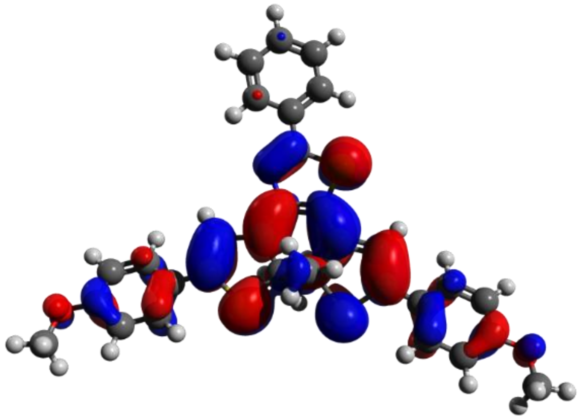

H-1

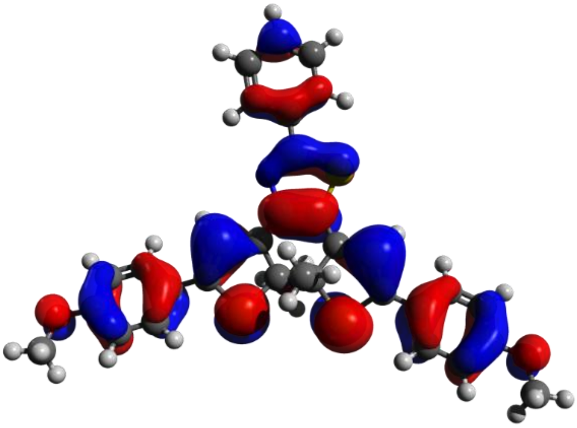

$1c^{+\bullet}$

L+1(α)

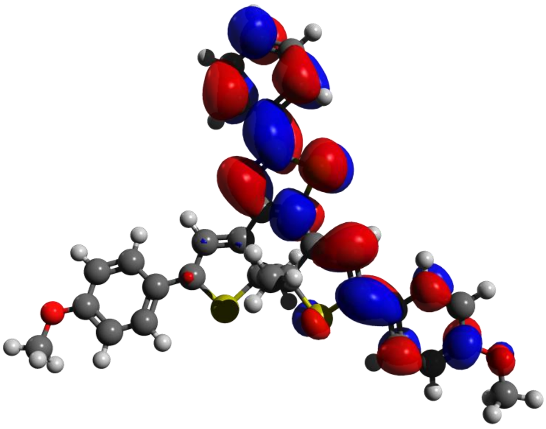

L(α)

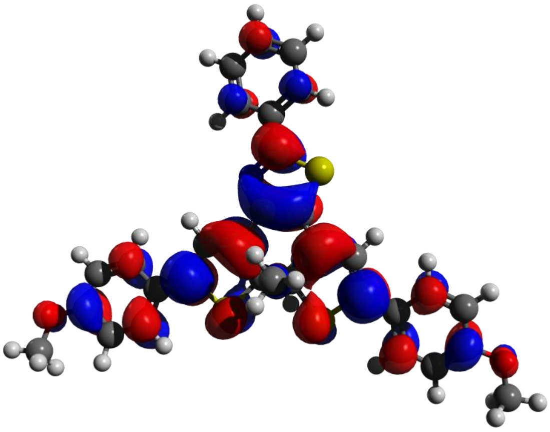

H(α)

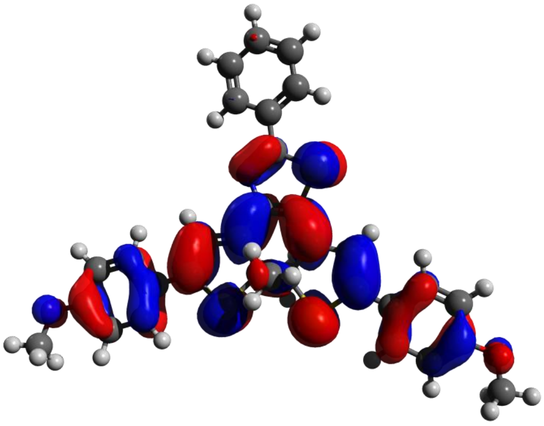

H-1(α)

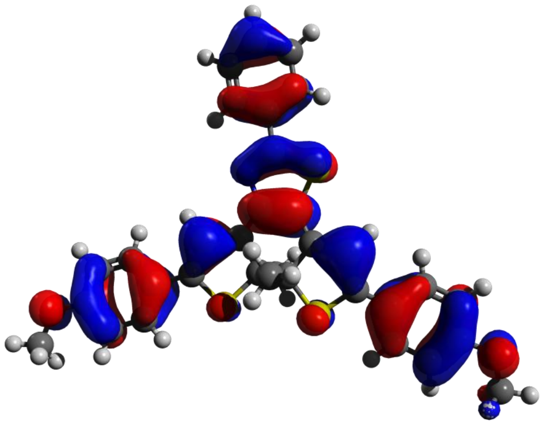

H-2(α)

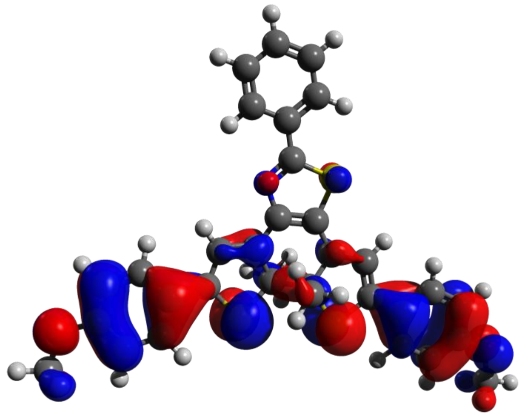

L(β)

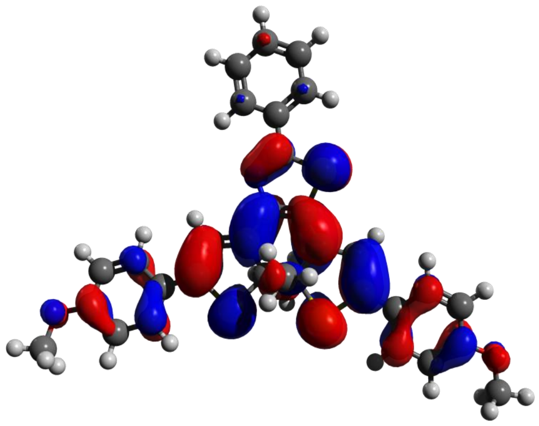

H(β)

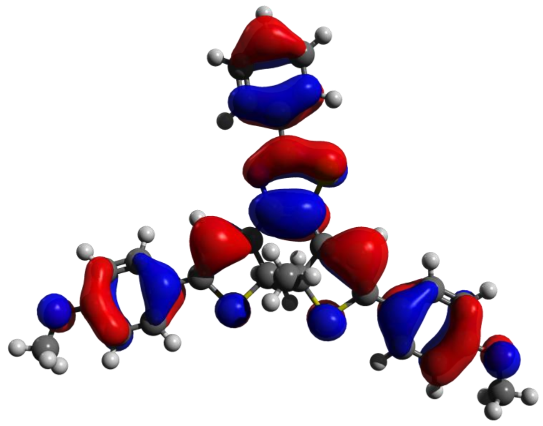

H-1(β)

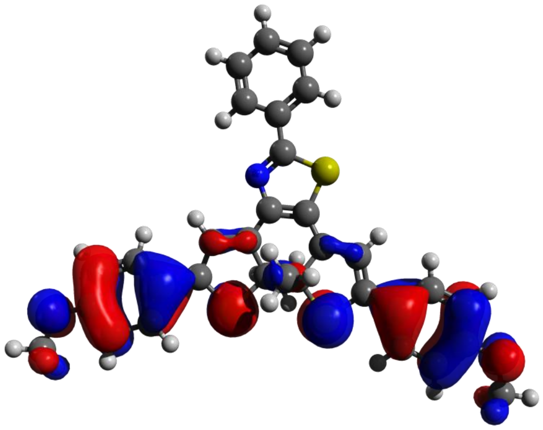

H-2(β)

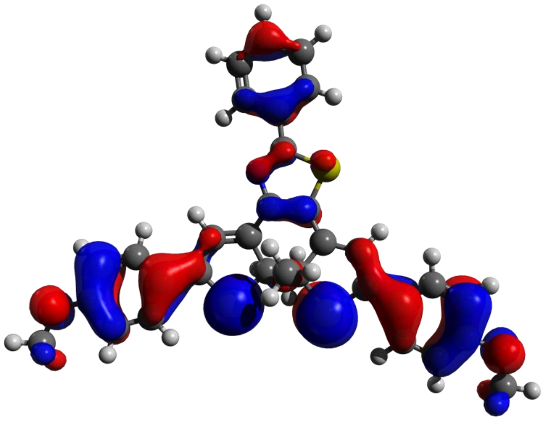

**$1c^{2+}$**

L+1

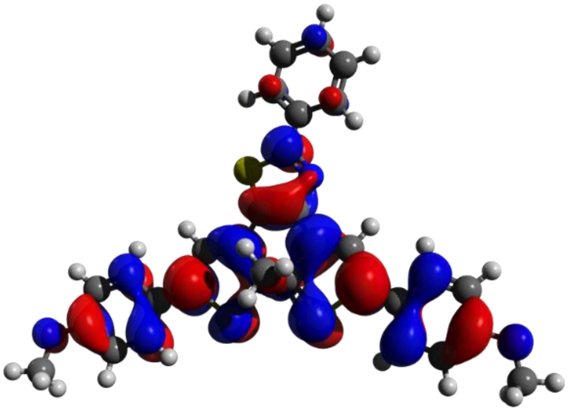

L

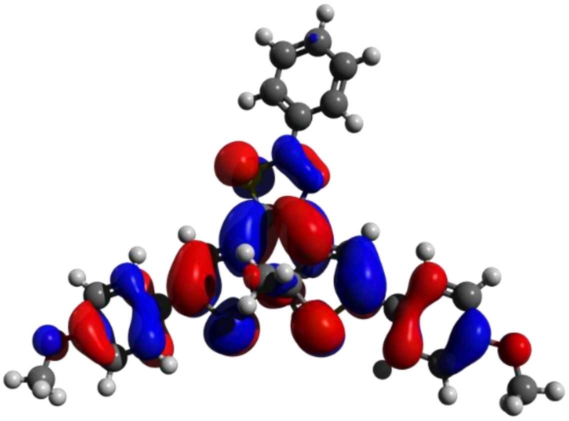

H

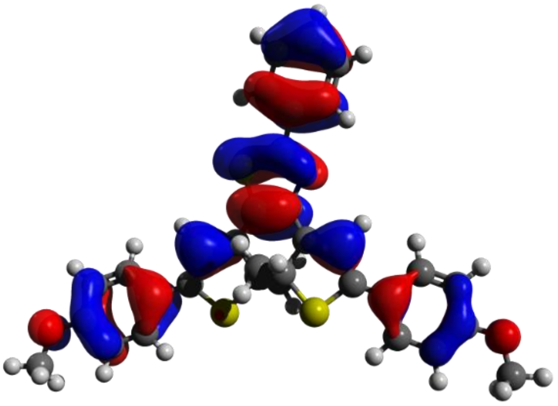

H-1

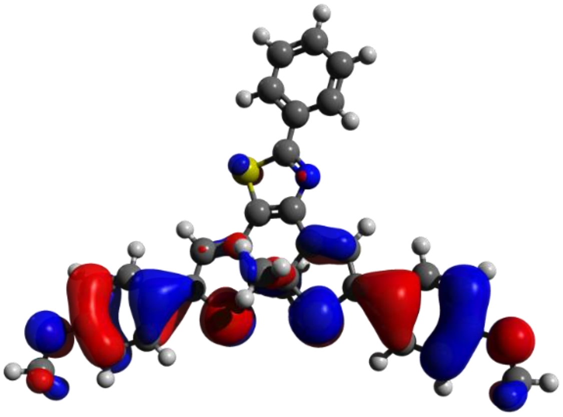

H-2

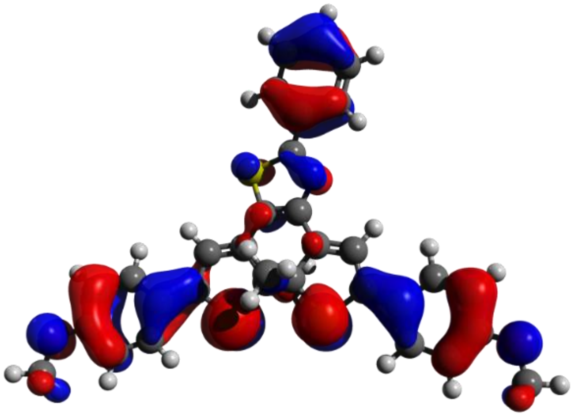

H-3

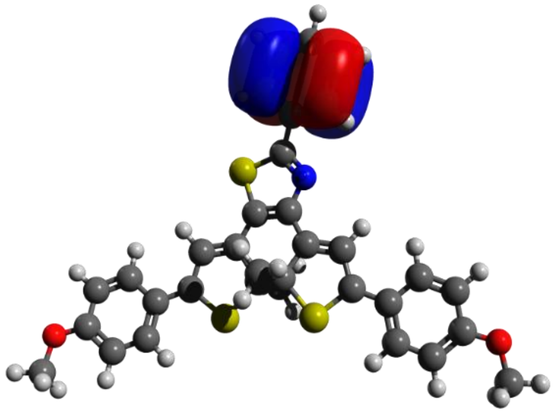

Figure S43: Calculated molecular orbital distribution implied in the electronic transitions for **1o**, **1c**, **1c**$^{+\bullet}$ and **1c**$^{2+}$ (ωB97X-D/6-311G(d,p) calculations).

1

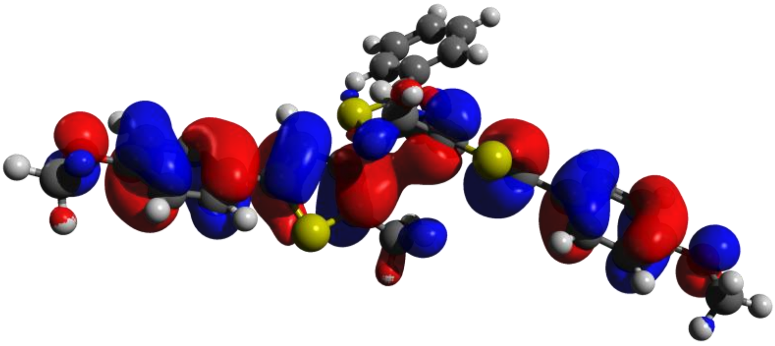

2

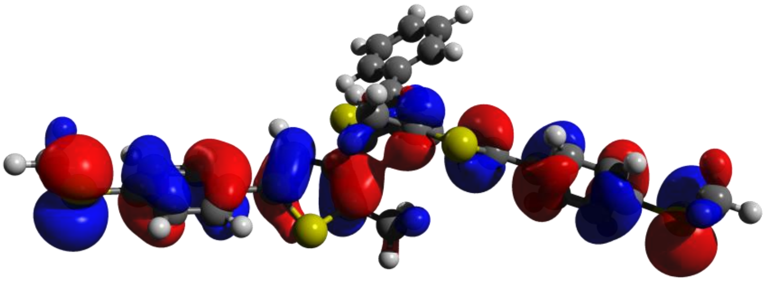

3

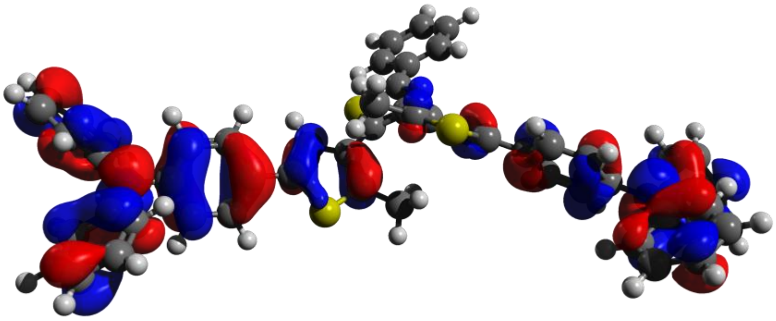

4

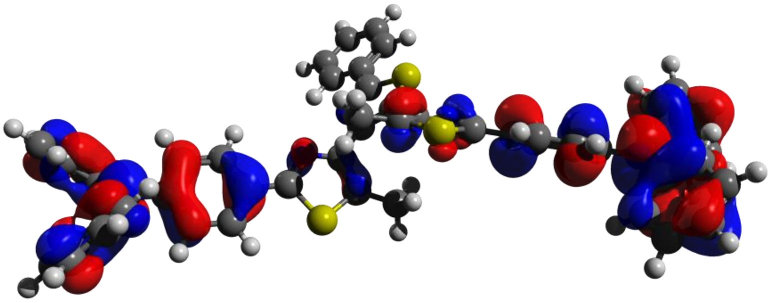

5

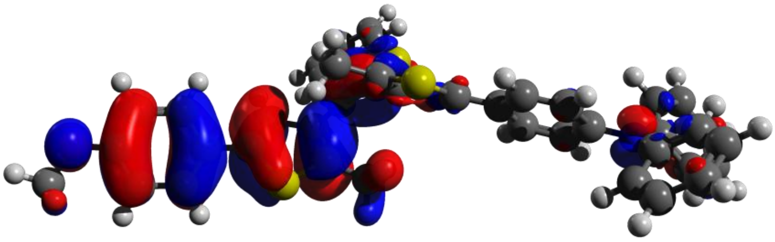

Figure S44: Calculated HOMO-1 of the OF isomers for compounds **1** – **5** (ωB97X-D/6-311G(d,p) calculations).

^{1}H- and ^{13}C-NMR spectra:

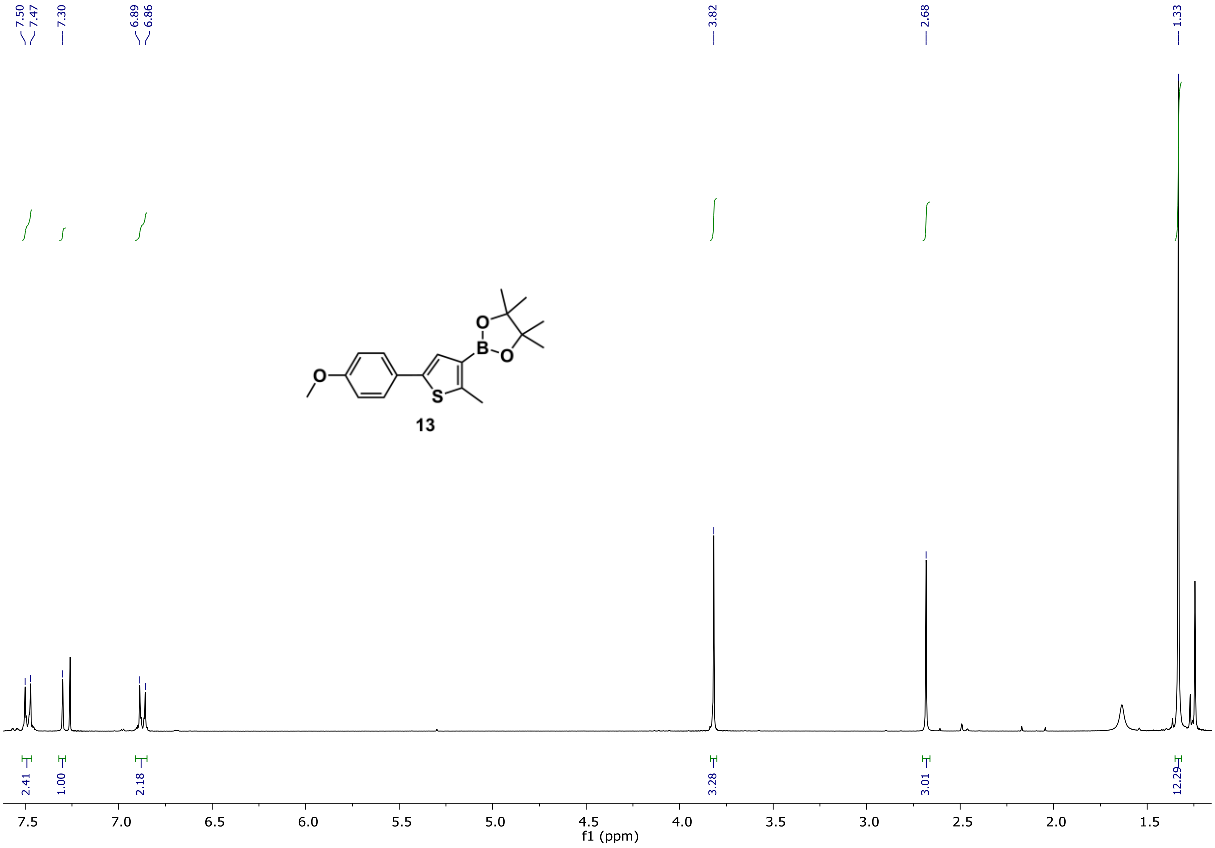

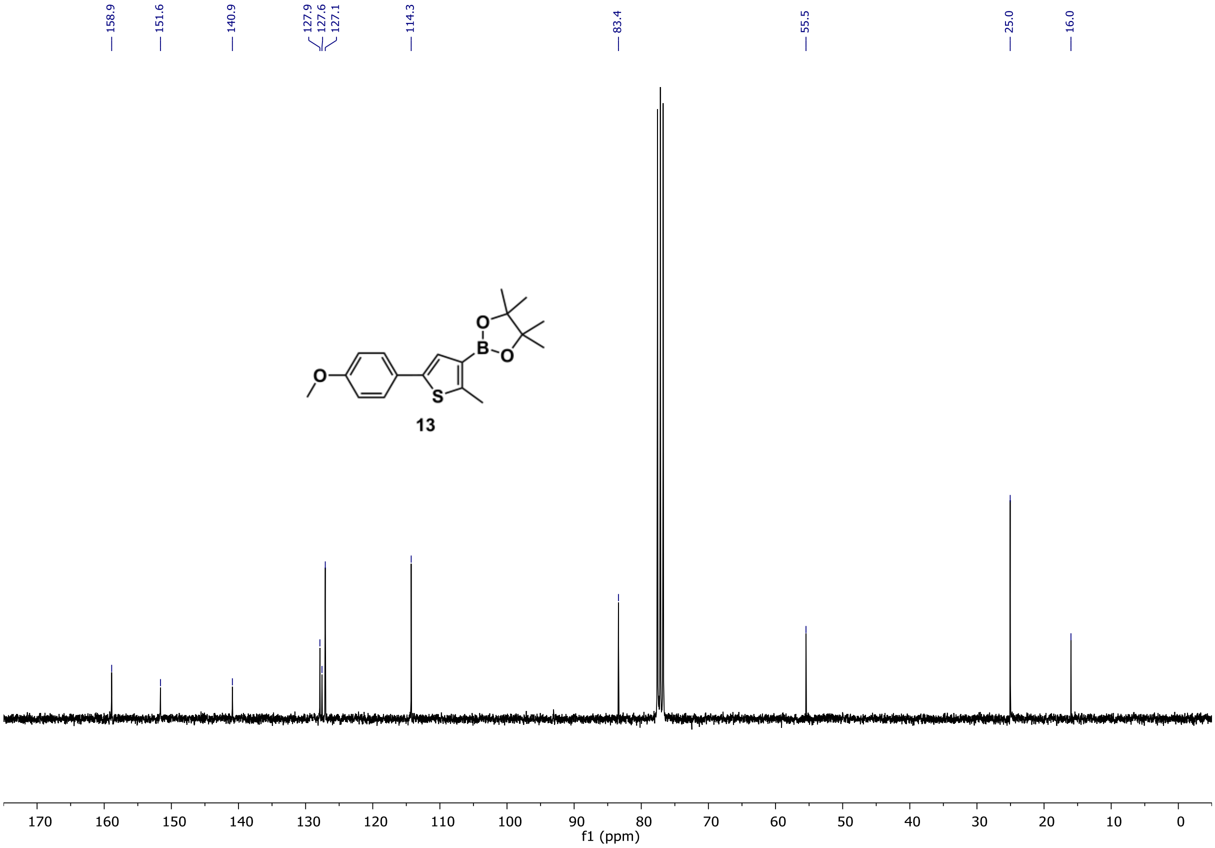

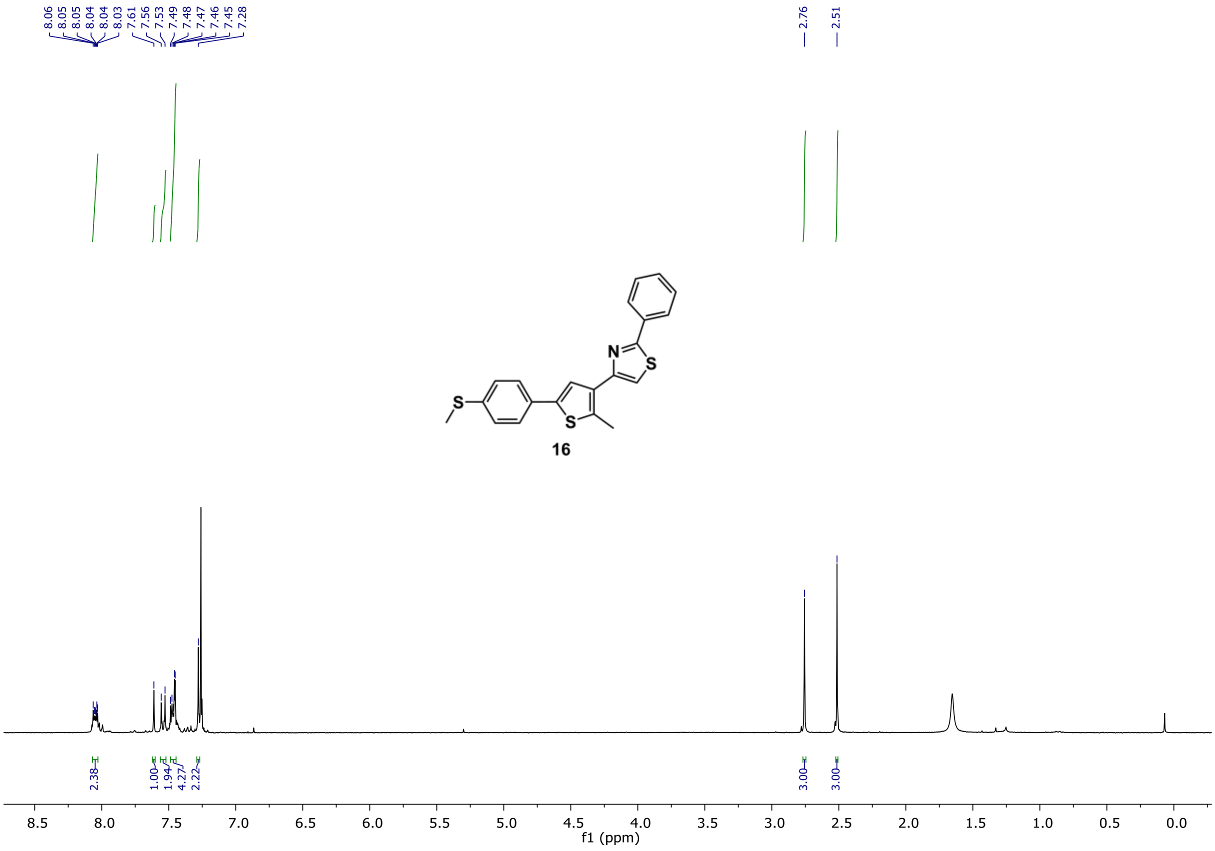
8.06
8.05
8.05
8.04
8.04
8.03
7.61
7.56
7.53
7.49
7.48
7.47
7.46
7.45
7.28
2.76
2.51
N
S
S
S
16
2.38
1.00
1.94
4.27
2.22
3.00
3.00
8.5 8.0 7.5 7.0 6.5 6.0 5.5 5.0 4.5 4.0 3.5 3.0 2.5 2.0 1.5 1.0 0.5 0.0
f1 (ppm)

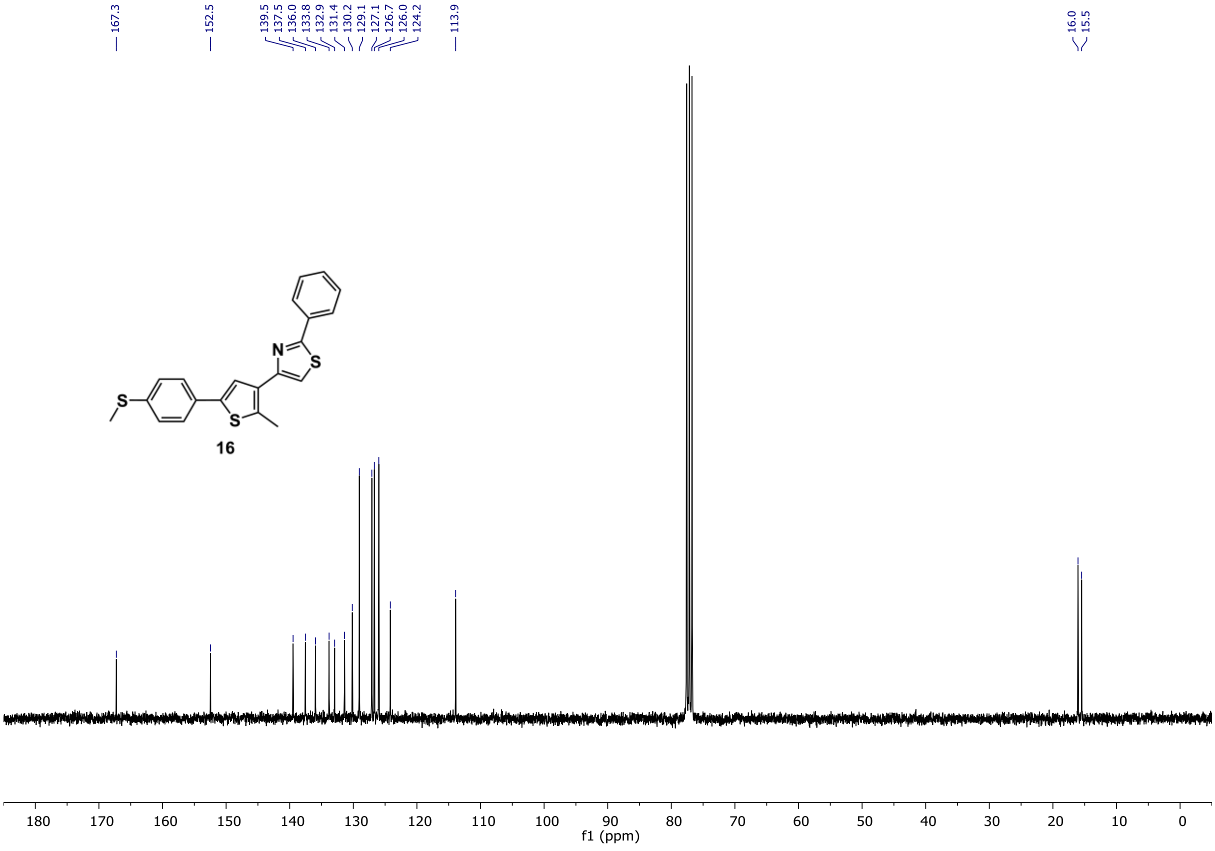
167.3
152.5
139.5
137.5
136.0
133.8
132.9
131.4
130.2
129.1
127.1
126.7
126.0
124.2
113.9
16.0
15.5
N
S
S
S
16
180 170 160 150 140 130 120 110 100 90 80 70 60 50 40 30 20 10 0
f1 (ppm)

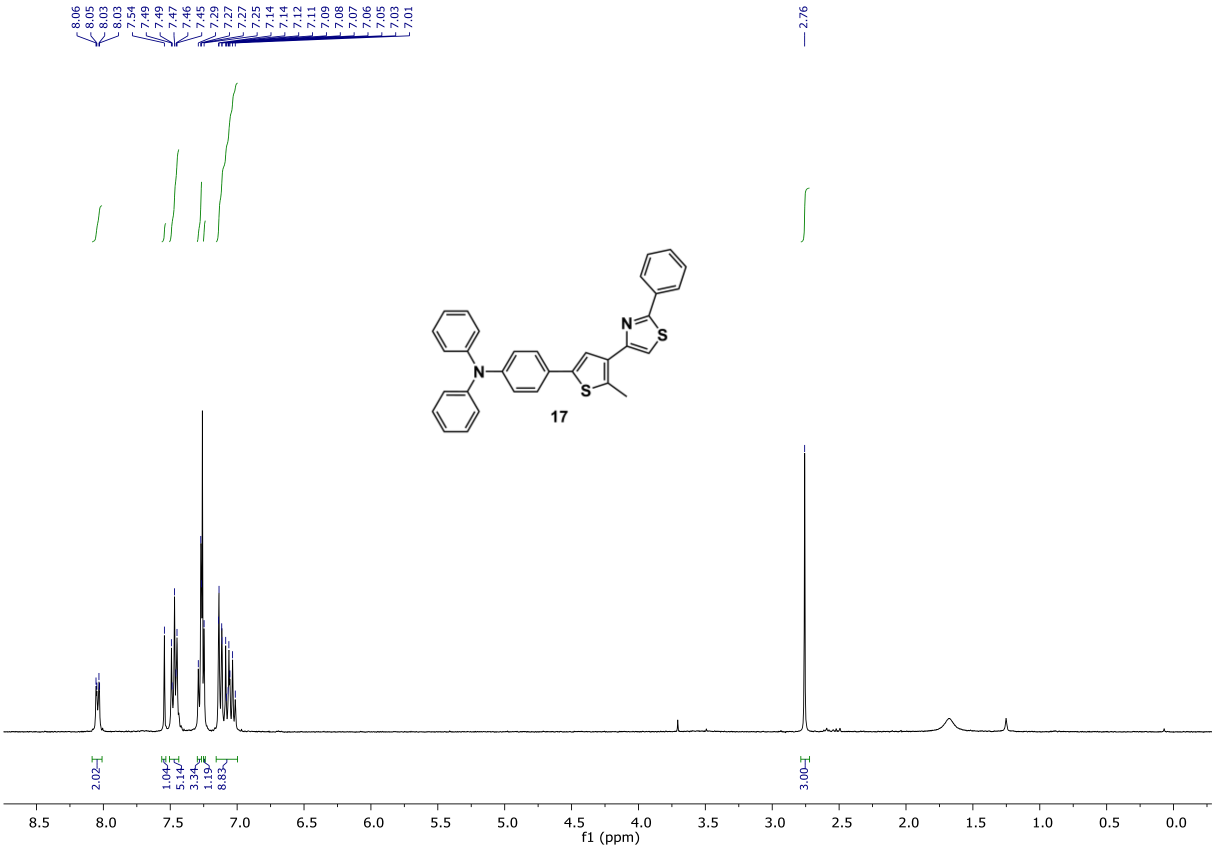
8.06
8.05
8.03
8.03
7.54
7.49
7.49
7.47
7.46
7.45
7.29
7.27
7.27
7.25
7.14
7.14
7.12
7.11
7.09
7.08
7.07
7.06
7.05
7.03
7.01
2.76
N
S
N
S
17
2.02
1.04
5.14
3.34
1.19
8.83
3.00
f1 (ppm)

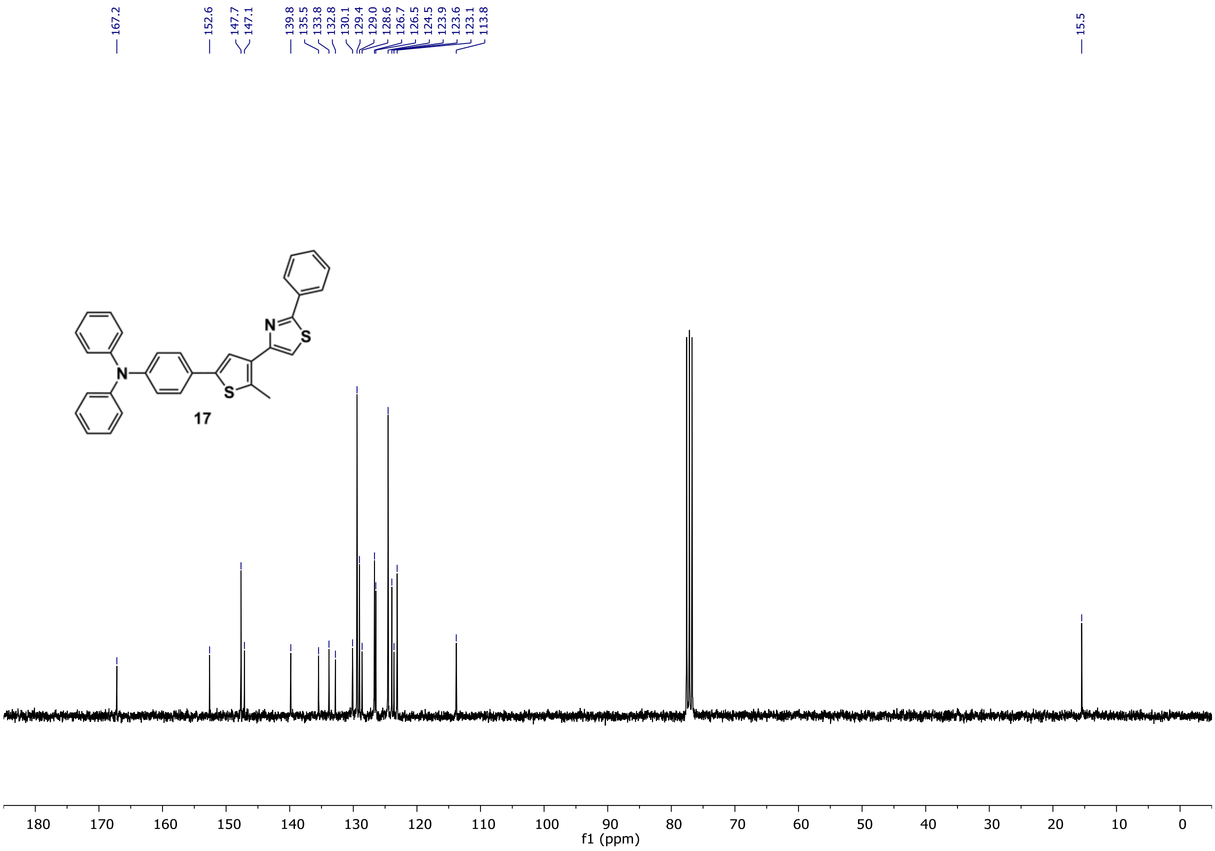
167.2
152.6
147.7
147.1
139.8
135.5
133.8
132.8
130.1
129.4
129.0
128.6
126.7
126.5
124.5
123.9
123.6
123.1
113.8
15.5
N
S
N
S
17
f1 (ppm)

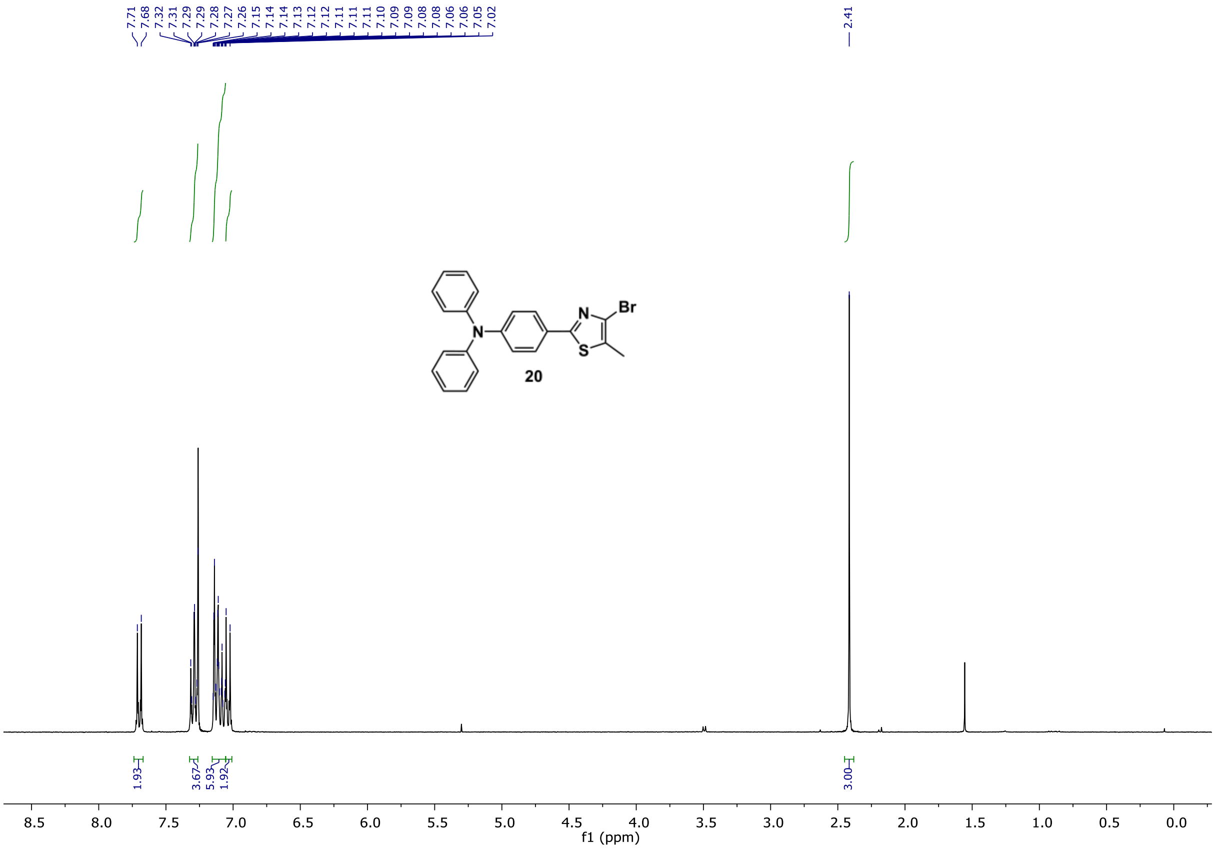
7.71
7.68
7.32
7.31
7.29
7.29
7.28
7.27
7.26
7.15
7.14
7.14
7.13
7.12
7.12
7.11
7.11
7.11
7.10
7.09
7.09
7.08
7.08
7.06
7.06
7.05
7.02
2.41
N
N
S
Br
20
1.93
3.67
5.93
1.92
3.00
8.5
8.0
7.5
7.0
6.5
6.0
5.5
5.0
4.5
4.0
3.5
3.0
2.5
2.0
1.5
1.0
0.5
0.0
f1 (ppm)

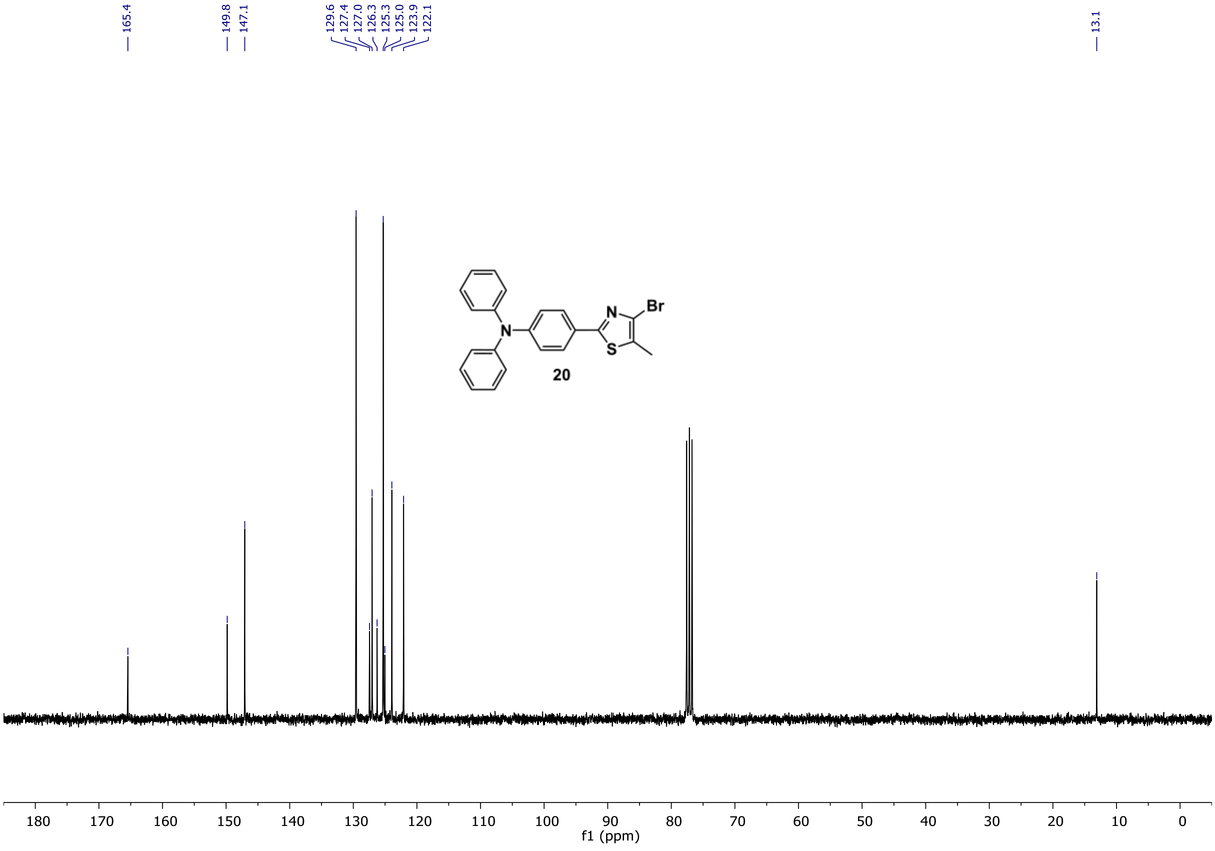
165.4
149.8
147.1
129.6
127.4
127.0
126.3
125.3
125.0
123.9
122.1
13.1
N
N
S
Br
20
180
170
160
150
140
130
120
110
100
90
80
70
60
50
40
30
20
10
0
f1 (ppm)

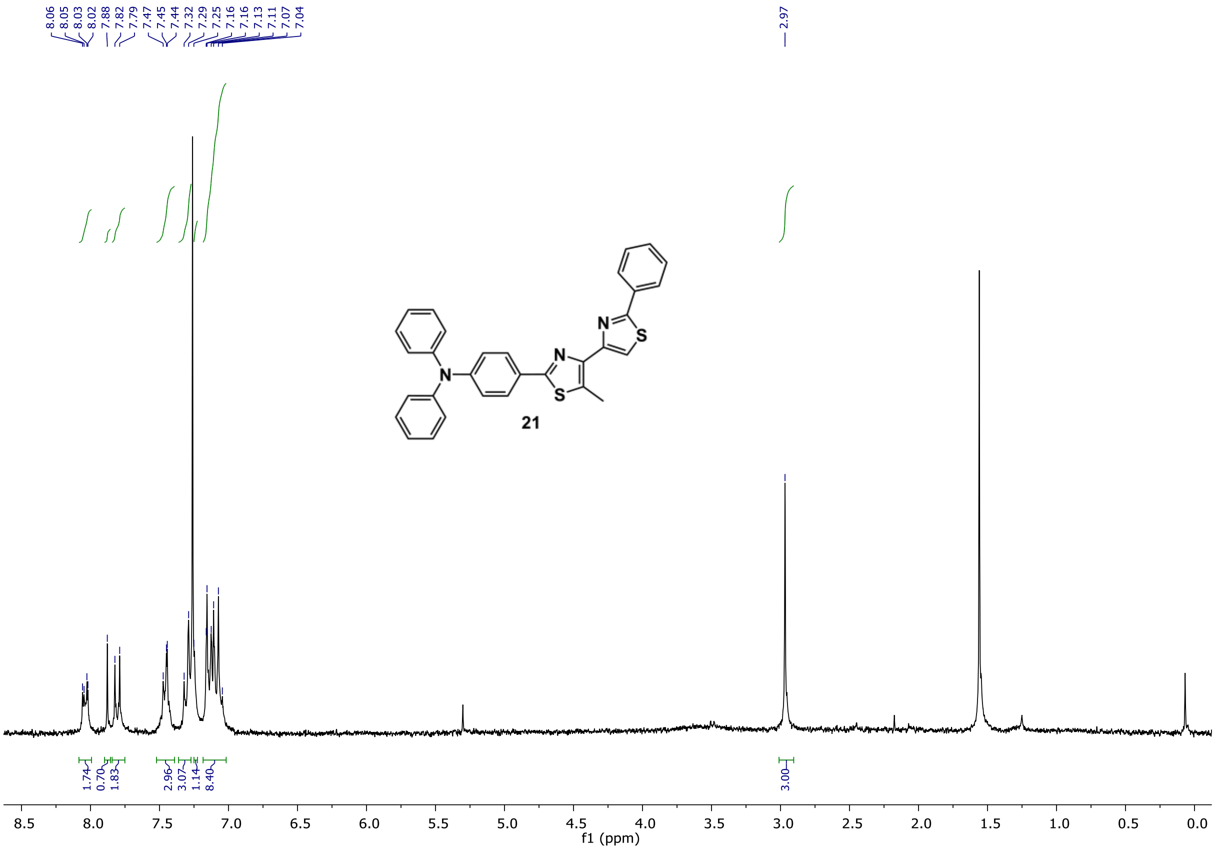
8.06
8.05
8.03
8.02
7.88
7.82
7.79
7.47
7.45
7.44
7.32
7.29
7.25
7.16
7.16
7.13
7.11
7.07
7.04
2.97
N
S
N
S
N
21
1.74
0.70
1.83
2.96
3.07
1.14
8.40
3.00
8.5 8.0 7.5 7.0 6.5 6.0 5.5 5.0 4.5 4.0 3.5 3.0 2.5 2.0 1.5 1.0 0.5 0.0
f1 (ppm)

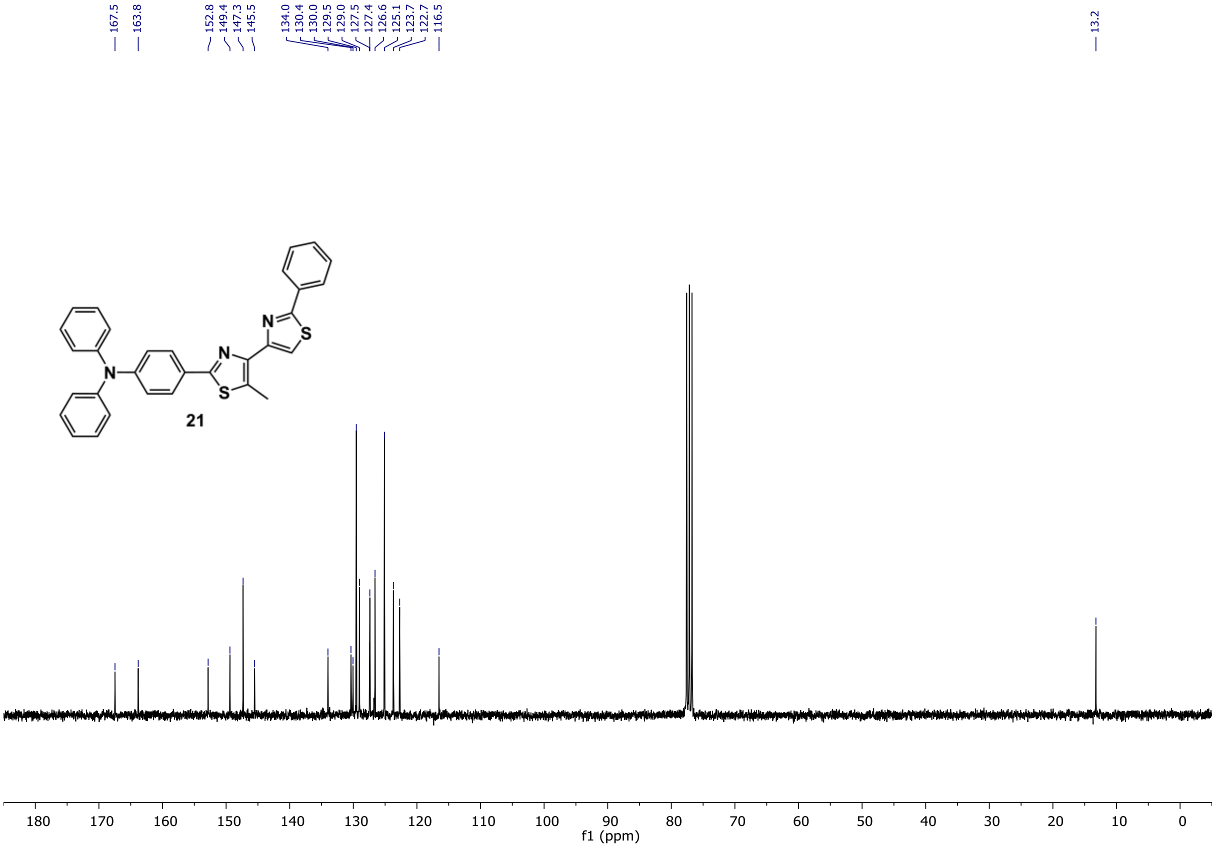
167.5
163.8
152.8
149.4
147.3
145.5
134.0
130.4
130.0
129.5
129.0
127.5
127.4
126.6
125.1
123.7
122.7
116.5
13.2
N
S
N
S
N
21
180 170 160 150 140 130 120 110 100 90 80 70 60 50 40 30 20 10 0
f1 (ppm)

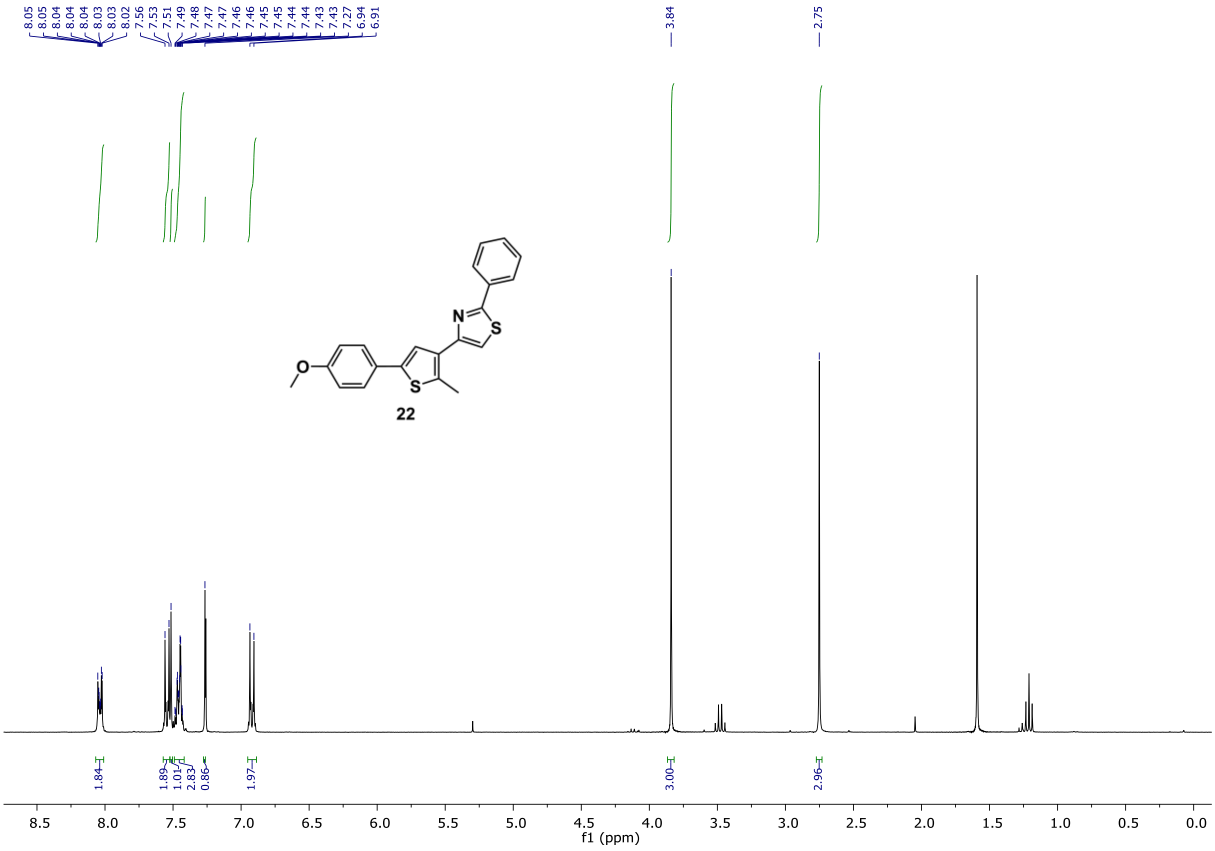
8.05
8.05
8.04
8.04
8.04
8.03
8.03
8.02
7.56
7.53
7.51
7.49
7.48
7.47
7.47
7.46
7.46
7.45
7.45
7.44
7.44
7.43
7.43
7.27
6.94
6.91
3.84
2.75
N
S
O
S
22
1.84
1.89
1.01
2.83
0.86
1.97
3.00
2.96
8.5
8.0
7.5
7.0
6.5
6.0
5.5
5.0
4.5
4.0
3.5
3.0
2.5
2.0
1.5
1.0
0.5
0.0
f1 (ppm)

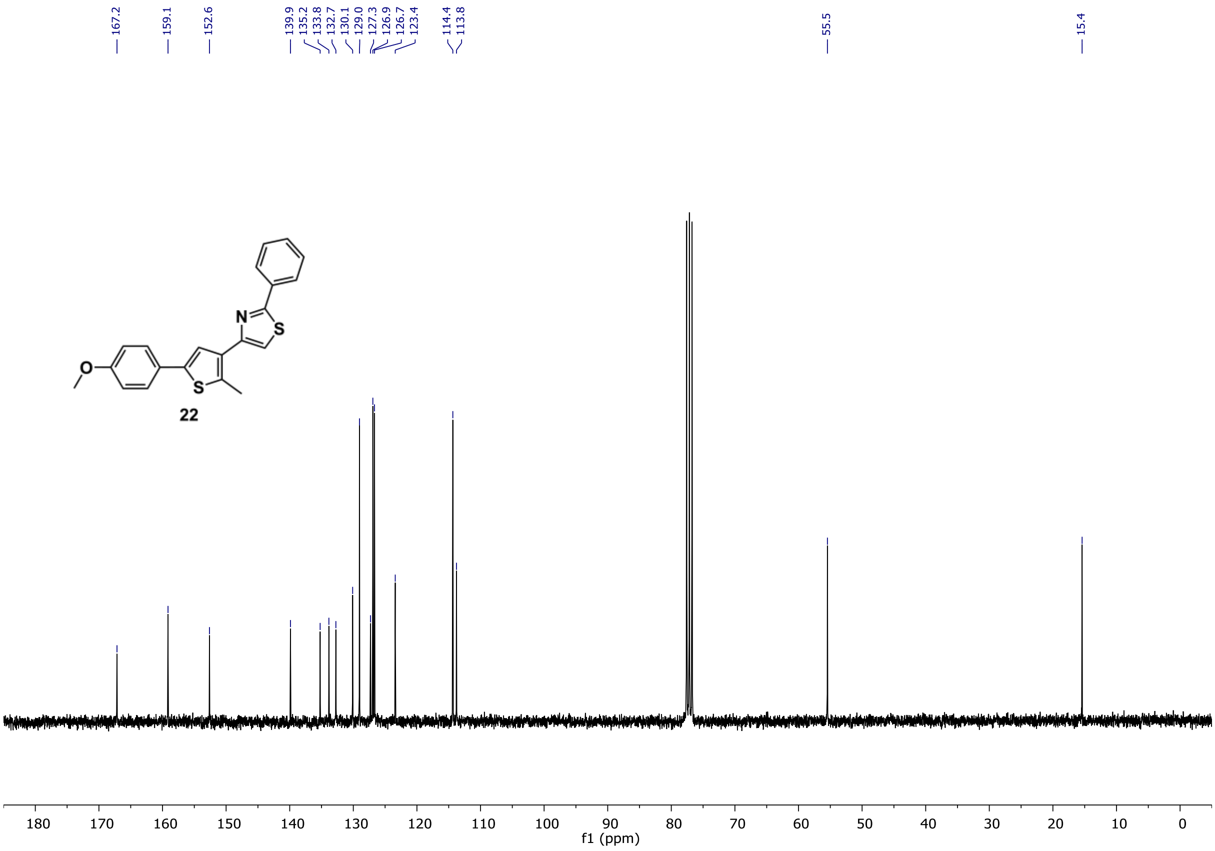
167.2
159.1
152.6
139.9
135.2
133.8
132.7
130.1
129.0
127.3
126.9
126.7
123.4
114.4
113.8
55.5
15.4
N
S
O
S
22
180
170
160
150
140
130
120
110
100
90
80
70
60
50
40
30
20
10
0
f1 (ppm)

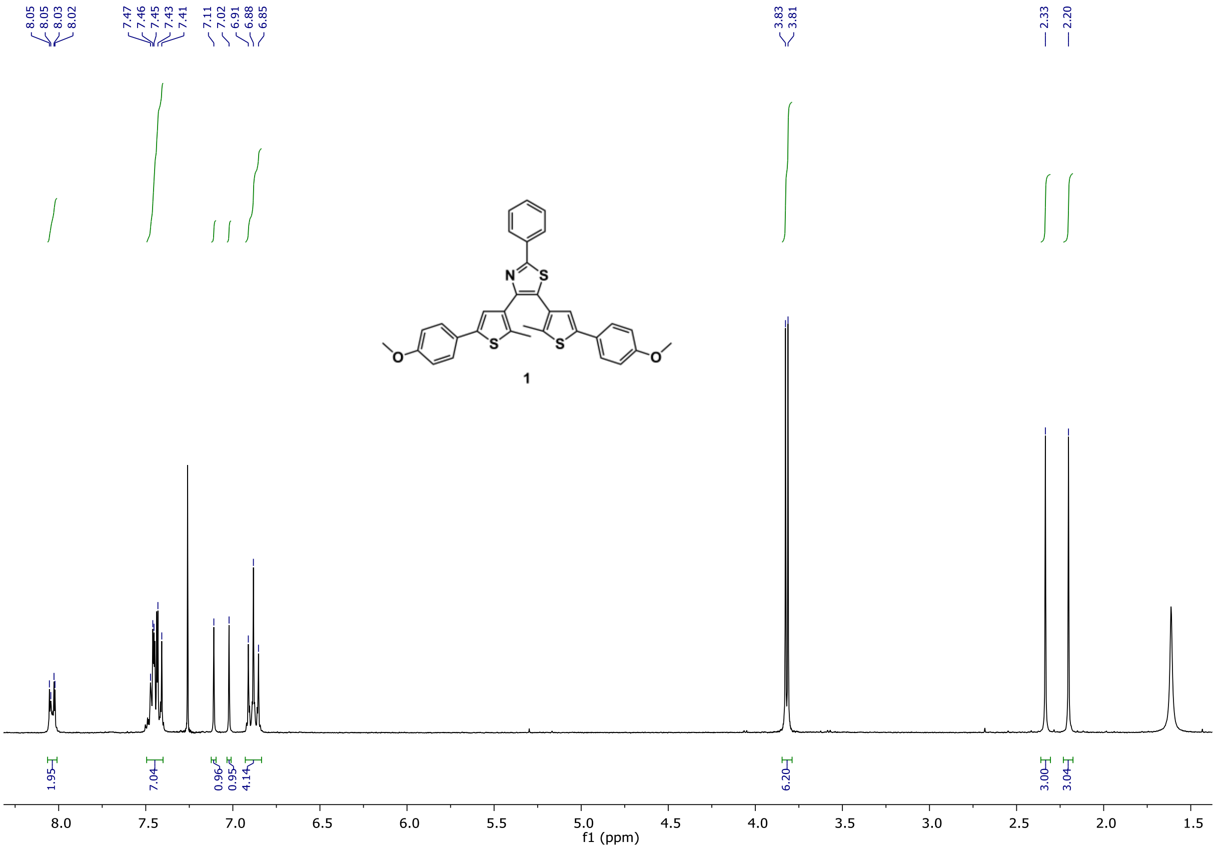

8.05
8.05
8.03
8.02
7.47
7.46
7.45
7.43
7.41
7.11
7.02
6.91
6.88
6.85
3.83
3.81
2.33
2.20
N
S
S
S
O
O
1
1.95
7.04
0.96
0.95
4.14
6.20
3.00
3.04
8.0 7.5 7.0 6.5 6.0 5.5 5.0 4.5 4.0 3.5 3.0 2.5 2.0 1.5
f1 (ppm)

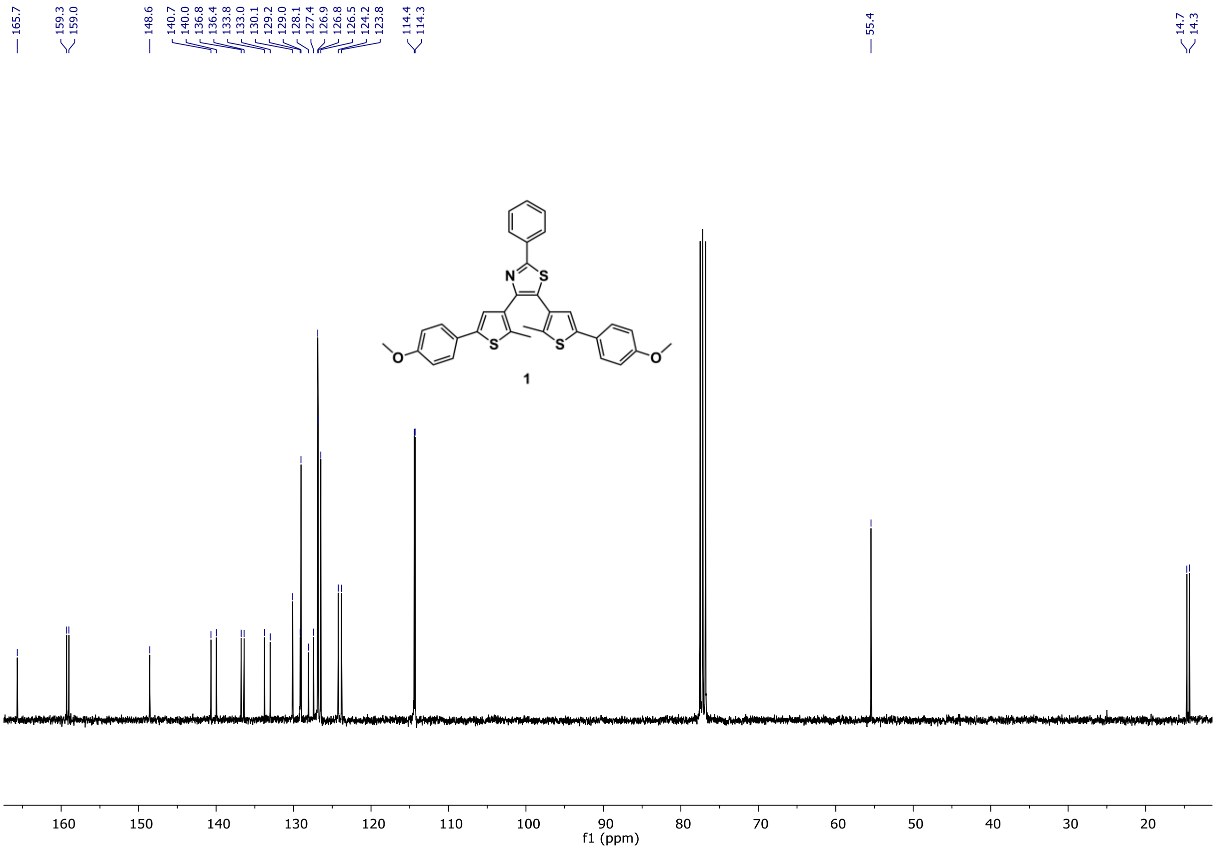

165.7
159.3
159.0
148.6
140.7
140.0
136.8
136.4
133.8
133.0
130.1
129.2
129.0
128.1
127.4
126.9
126.8
126.5
124.2
123.8
114.4
114.3
55.4
14.7
14.3
N
S
S
S
O
O
1
160 150 140 130 120 110 100 90 80 70 60 50 40 30 20
f1 (ppm)

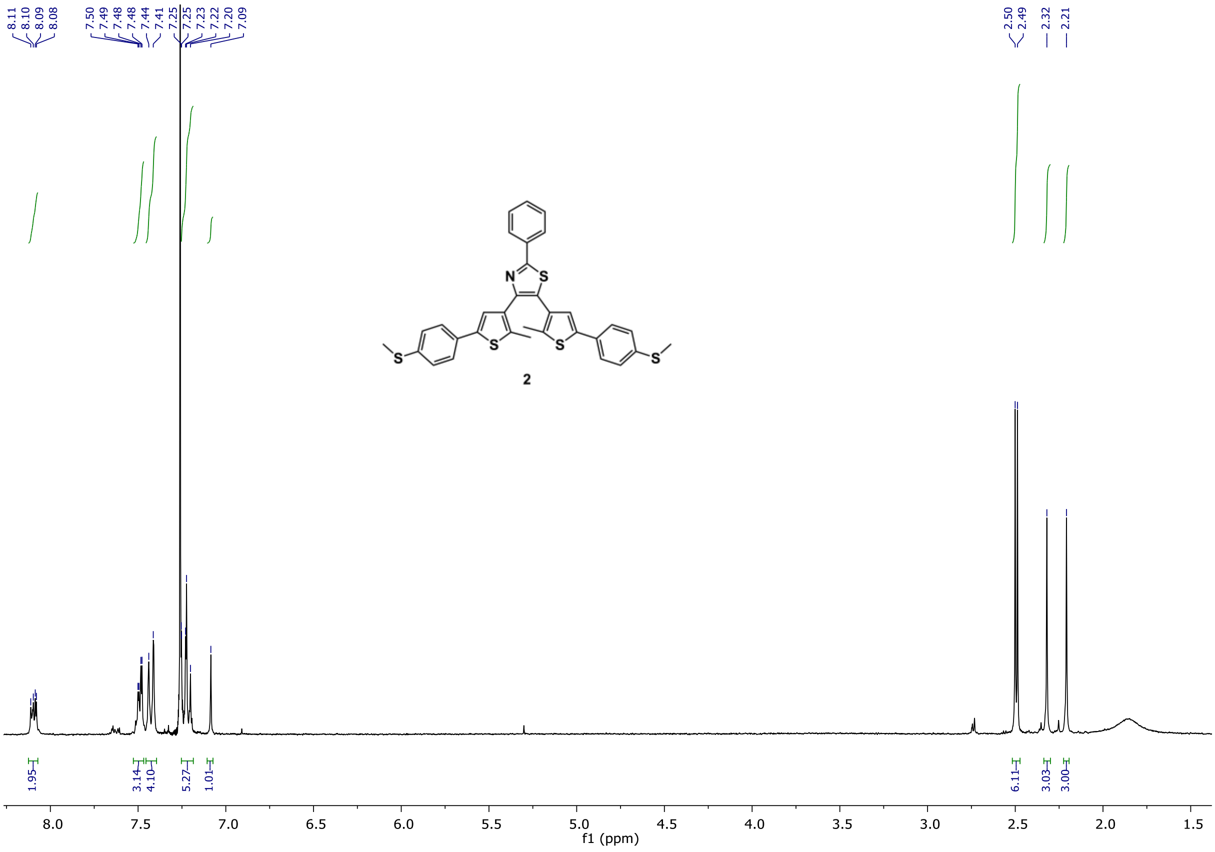
8.11
8.10
8.09
8.08
7.50
7.49
7.48
7.48
7.44
7.41
7.25
7.25
7.23
7.22
7.20
7.09
2.50
2.49
2.32
2.21
N
S
S
S
S
S
2
1.95
3.14
4.10
5.27
1.01
6.11
3.03
3.00
8.0
7.5
7.0
6.5
6.0
5.5
5.0
4.5
4.0
3.5
3.0
2.5
2.0
1.5
f1 (ppm)

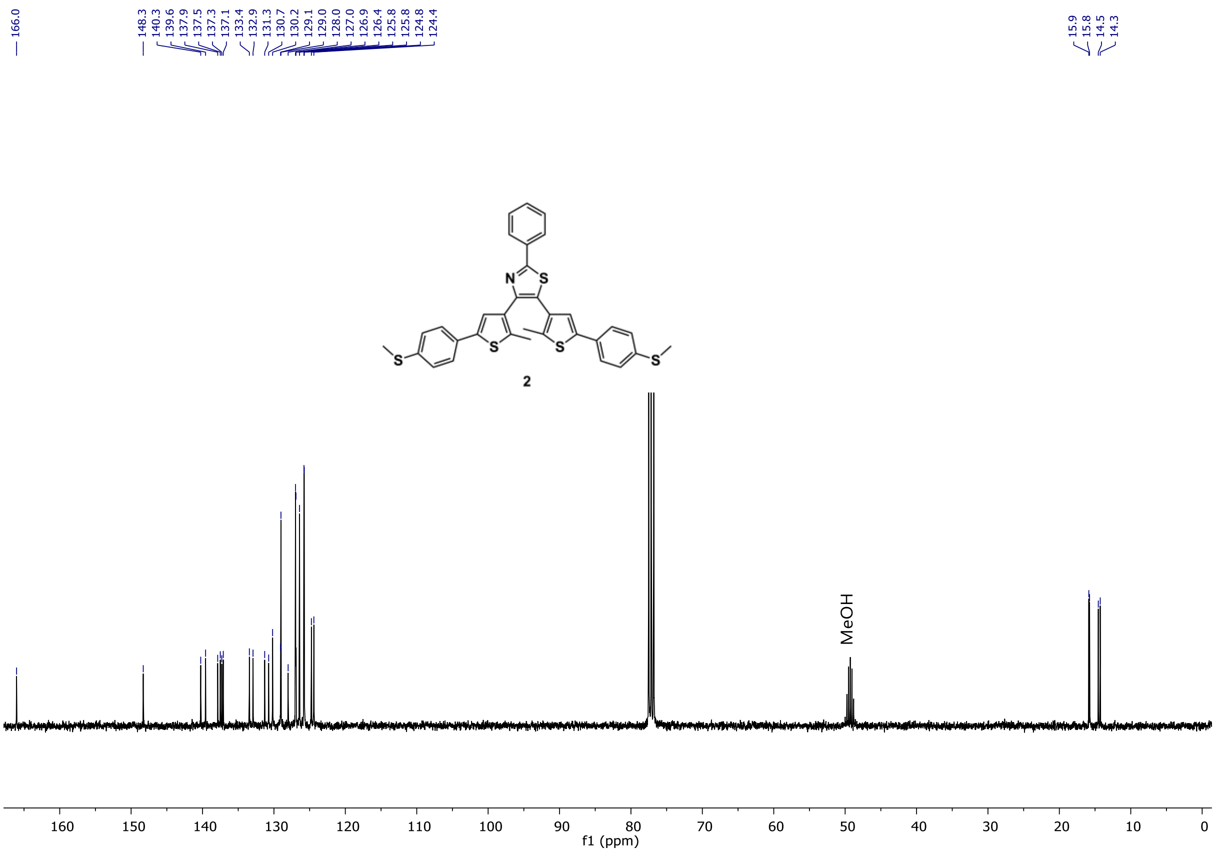
166.0
148.3
140.3
139.6
137.9
137.5
137.3
137.1
133.4
132.9
131.3
130.7
130.2
129.1
129.0
128.0
127.0
126.9
126.4
125.8
125.8
124.8
124.4
15.9
15.8
14.5
14.3
N
S
S
S
S
S
2
MeOH
160
150
140
130
120
110
100
90
80
70
60
50
40
30
20
10
0
f1 (ppm)

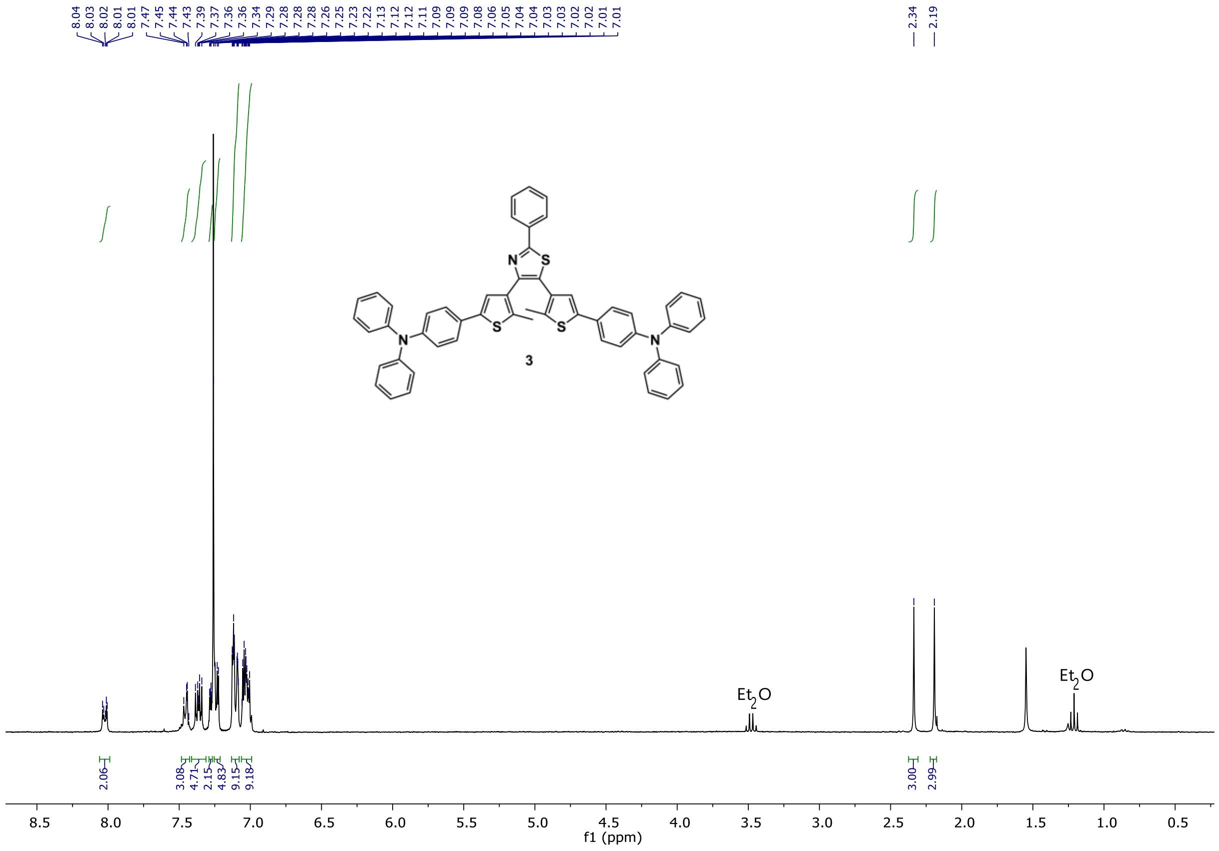
3
Et2O
Et2O
f1 (ppm)

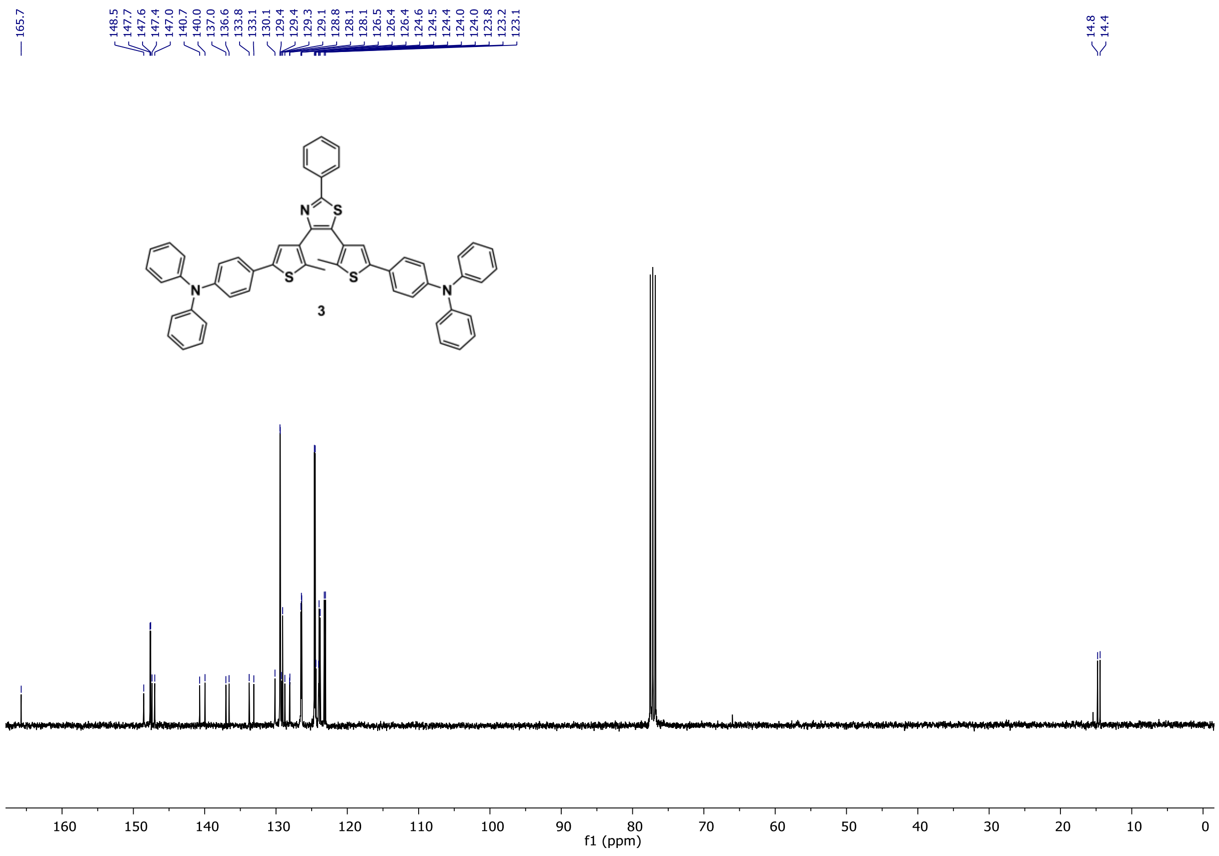
3
f1 (ppm)

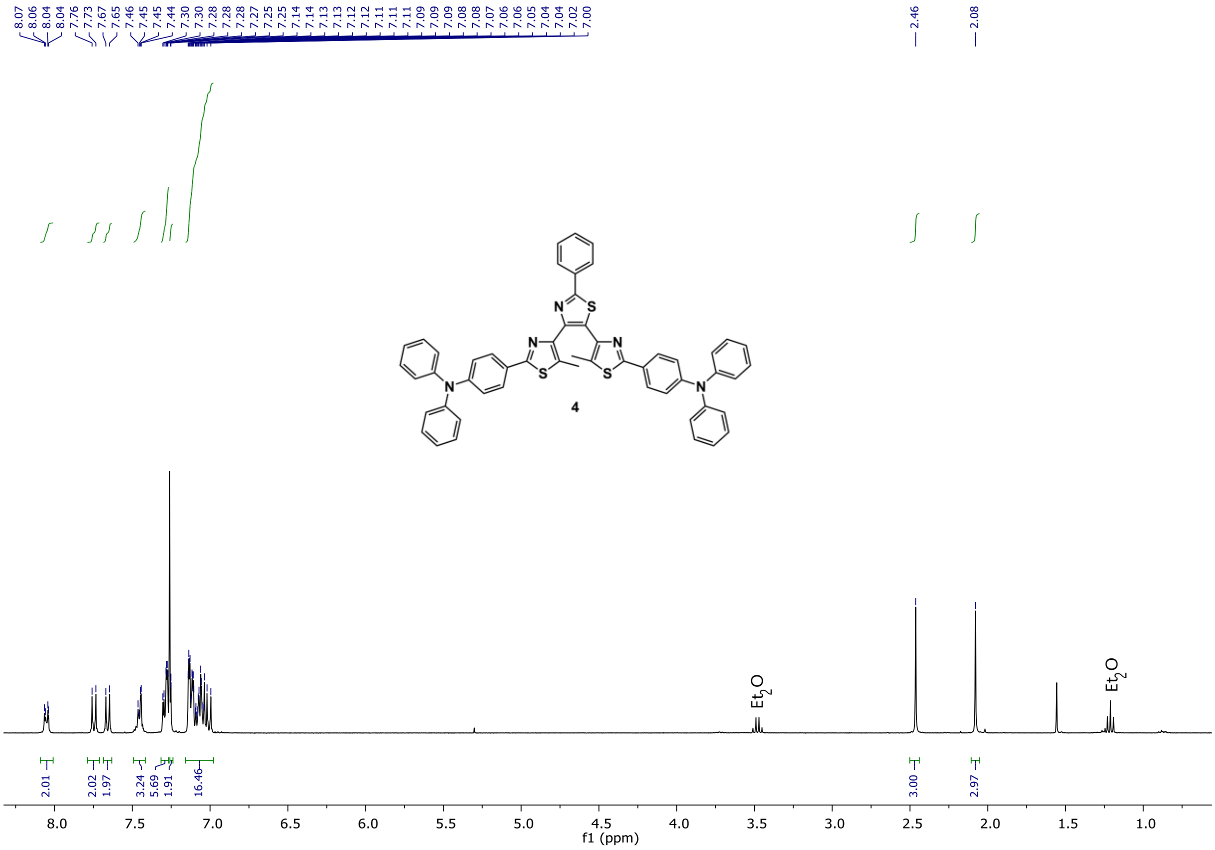
8.07 8.06 8.04 8.04 7.76 7.73 7.67 7.65 7.46 7.45 7.45 7.44 7.30 7.30 7.28 7.28 7.28 7.27 7.25 7.25 7.14 7.14 7.13 7.13 7.12 7.12 7.11 7.11 7.11 7.09 7.09 7.09 7.08 7.08 7.07 7.06 7.06 7.05 7.04 7.04 7.02 7.00
2.46
2.08
N
S
4
Et2O
Et2O
2.01 2.02 1.97 3.24 5.69 1.91 16.46
3.00
2.97
8.0 7.5 7.0 6.5 6.0 5.5 5.0 4.5 4.0 3.5 3.0 2.5 2.0 1.5 1.0
f1 (ppm)

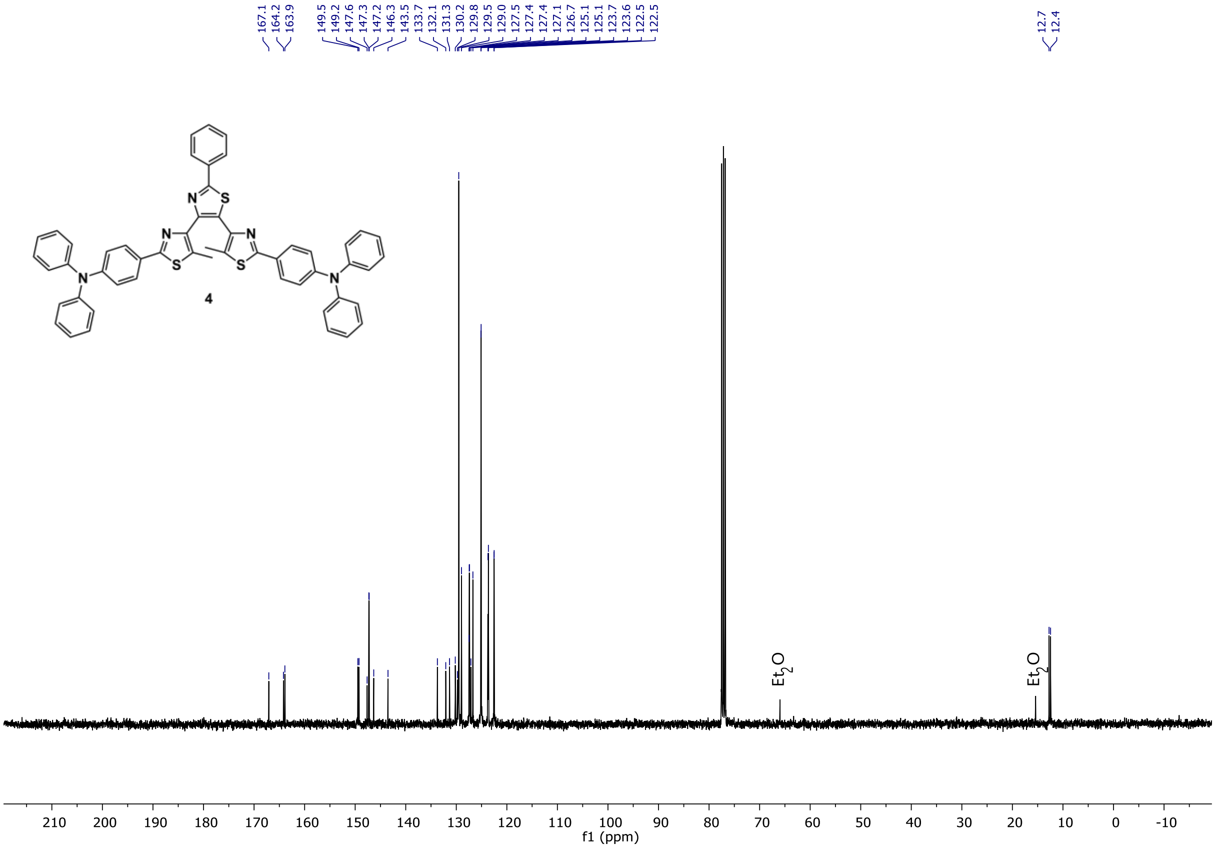
167.1 164.2 163.9
149.5 149.2 147.6 147.3 147.2 146.3 143.5 133.7 132.1 131.3 130.2 129.8 129.5 129.0 127.5 127.4 127.1 126.7 125.1 125.1 123.7 123.6 122.5 122.5
12.7 12.4
N
S
4
Et2O
Et2O
210 200 190 180 170 160 150 140 130 120 110 100 90 80 70 60 50 40 30 20 10 0 -10
f1 (ppm)

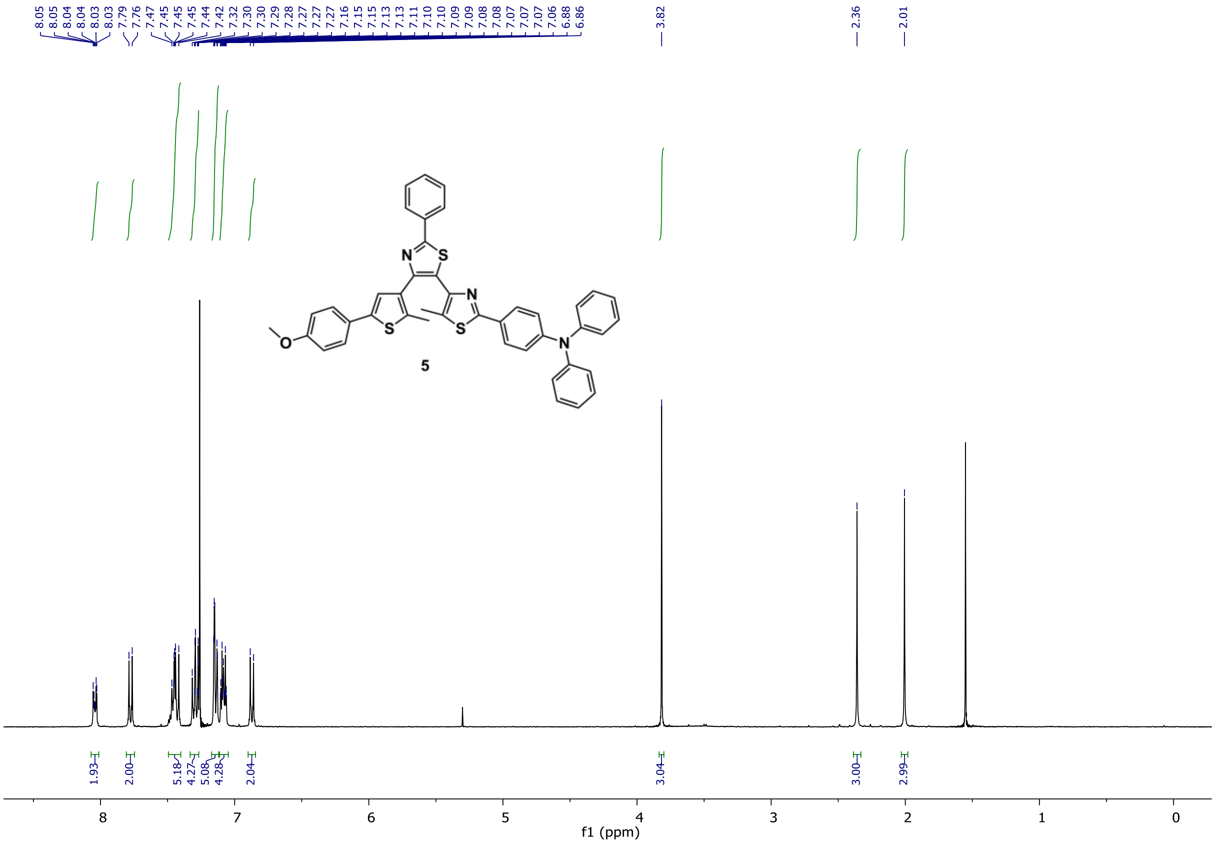
8.05 8.05 8.04 8.04 8.03 8.03 7.79 7.76 7.47 7.45 7.45 7.45 7.44 7.42 7.32 7.30 7.30 7.29 7.28 7.27 7.27 7.27 7.16 7.15 7.15 7.13 7.13 7.11 7.10 7.10 7.09 7.09 7.08 7.08 7.07 7.07 7.07 7.06 6.88 6.86
3.82
2.36
2.01
N
S
N
S
O
N
5
1.93
2.00
5.18
4.27
5.08
4.28
2.04
3.04
3.00
2.99
8 7 6 5 4 3 2 1 0
f1 (ppm)

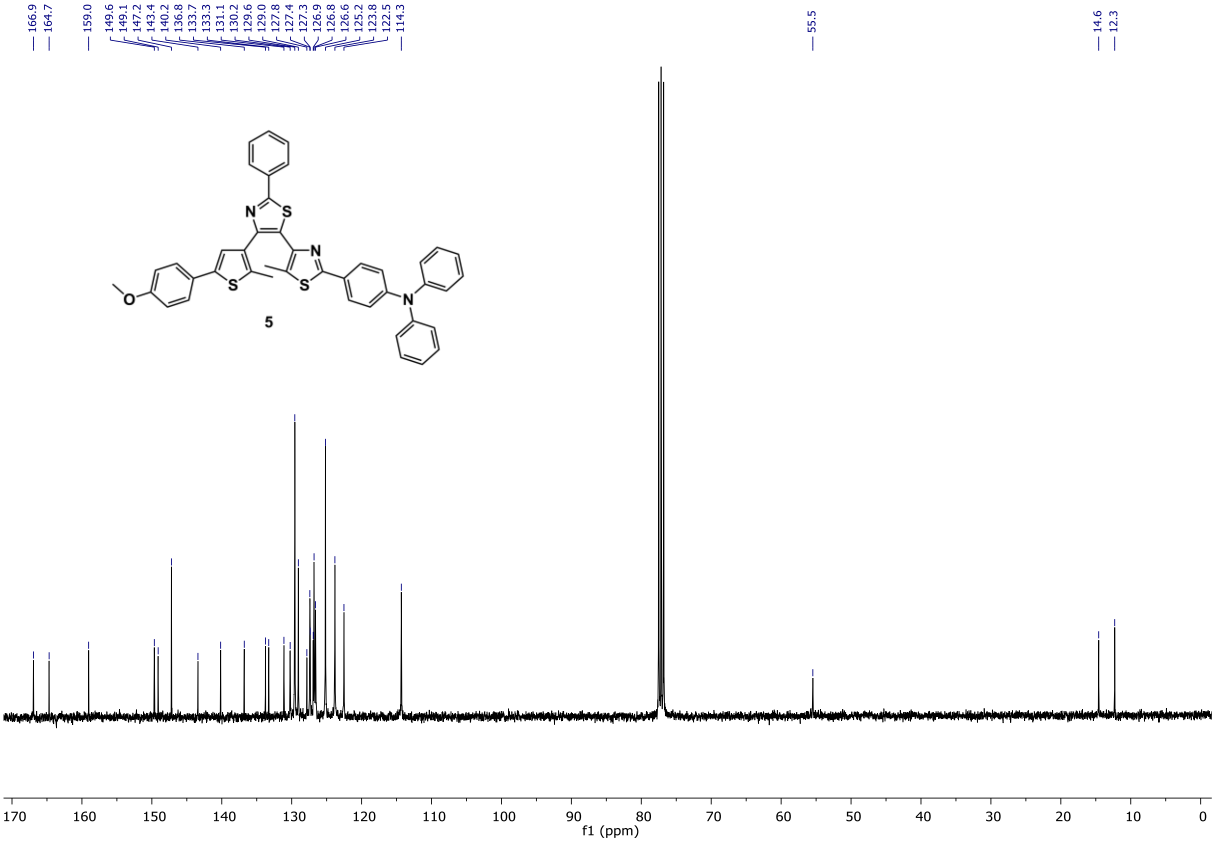
166.9 164.7
159.0
149.6 149.1 147.2 143.4 140.2 136.8 133.7 133.3 131.1 130.2 129.6 129.0 127.8 127.4 127.3 126.9 126.8 126.6 125.2 123.8 122.5
114.3
55.5
14.6 12.3
N
S
N
S
O
N
5
170 160 150 140 130 120 110 100 90 80 70 60 50 40 30 20 10 0
f1 (ppm)

References:

1 K. A. Johnson, Z. B. Simpson and T. Blom, *Anal. Biochem.*, 2009, **387**, 20–29.

2 K. A. Johnson, Z. B. Simpson and T. Blom, *Anal. Biochem.*, 2009, **387**, 30–41.

3 Y. Zhao and D. G. Truhlar, *Theor. Chem. Acc.*, 2008, **120**, 215–241.

4 C. Adamo and V. Barone, *J. Chem. Phys.*, 1999, **110**, 6158–6170.

5 H. S. Yu, X. He, S. L. Li and D. G. Truhlar, *Chem. Sci.*, 2016, **7**, 5032–5051.

6 T. Yanai, D. P. Tew and N. C. Handy, *Chem. Phys. Lett.*, 2004, **393**, 51–57.

7 C. Adamo and V. Barone, *J. Chem. Phys.*, 1998, **108**, 664–675.

8 J. Da Chai and M. Head-Gordon, *Phys. Chem. Chem. Phys.*, 2008, **10**, 6615–6620.

9 A. V. Marenich, C. J. Cramer and D. G. Truhlar, *J Phys Chem B*, 2009, **113**, 6378–6396.

10 M. J. Frisch, G. W. Trucks, H. B. Schlegel, G. E. Scuseria, M. A. Robb, J. R. Cheeseman, G. Scalmani, V. Barone, G. A. Petersson, H. Nakatsuji, X. Li, M. Caricato, A. V. Marenich, J. Bloino, B. G. Janesko, R. Gomperts, B. Mennucci, H. P. Hratchian, J. V. Ortiz, A. F. Izmaylov, J. L. Sonnenberg, D. Williams-Young, F. Ding, F. Lipparini, F. Egidi, J. Goings, B. Peng, A. Petrone, T. Henderson, D. Ranasinghe, V. G. Zakrzewski, J. Gao, N. Rega, G. Zheng, W. Liang, M. Hada, M. Ehara, K. Toyota, R. Fukuda, J. Hasegawa, M. Ishida, T. Nakajima, Y. Honda, O. Kitao, H. Nakai, T. Vreven, K. Throssell, J. A. Montgomery, Jr., J. E. Peralta, F. Ogliaro, M. J. Bearpark, J. J. Heyd, E. N. Brothers, K. N. Kudin, V. N. Staroverov, T. A. Keith, R. Kobayashi, J. Normand, K. Raghavachari, A. P. Rendell, J. C. Burant, S. S. Iyengar, J. Tomasi, M. Cossi, J. M. Millam, M. Klene, C. Adamo, R. Cammi, J. W. Ochterski, R. L. Martin, K. Morokuma, O. Farkas, J. B. Foresman, and D. J. Fox, Gaussian, Inc., Wallingford CT, 2016.

11 S. M. Büllmann, T. Kolmar, P. Slawetzky, S. Wald and A. Jäschke, *Chem. Commun.,* 2021, **57**, 6596-6599.

12 G. Sevez and J. L. Pozzo, *Dyes Pigm.*, 2011, **89**, 246–253.

13 T. Nakahama, D. Kitagawa and S. Kobatake, *J. Phys. Chem. C*, 2019, **123**, 31212–31218.

14 M. Schnürch, J. Hämmerle, M. D. Mihovilovic and P. Stanetty, *Synthesis*, 2010, **5**, 837–843.

15 M. Begtrup, L. B. L. Hansen, S. Grundvig, Y. Stenstrøm, A. Z.-Q. Khan, J. Sandström and P. Krogsgaard-Larsen, *Acta. Chem. Scand.*, 1992, **46**, 372–383.

16 N. Baggi, A. Léaustic, S. Groni, E. Anxolabéhère-Mallart, R. Guillot, R. Métivier, F. Maurel and P. Yu, *Chem. Eur. J.*, 2021, **27**, 12866–12876.

17 Y. Wang, E. I. Rogers and R. G. Compton, *J. Electroanal. Chem.*, 2010, **648**, 15–19.

18 W. R. Browne, J. J. D. De Jong, T. Kudernac, M. Walko, L. N. Lucas, K. Uchida, J. H. Van Esch and B. L. Feringa, *Chem. Eur. J.*, 2005, **11**, 6414–6429.

19 M. Herder, M. Utecht, N. Manicke, L. Grubert, M. Pätzel, P. Saalfrank and S. Hecht, *Chem. Sci.*, 2013, **4**, 1028–1040.
